# Structural and physical properties of 99 complex bulk chalcogenides crystals using first-principles calculations

**DOI:** 10.1038/s41598-021-89281-6

**Published:** 2021-05-10

**Authors:** Sahib Hasan, Khagendra Baral, Neng Li, Wai-Yim Ching

**Affiliations:** 1 Department of Physics and Astronomy, University of Missouri-Kansas City, Kansas City, Missouri 64110 USA; 2 School of Materials Science and Engineering, Wuhan University of Technology, No. 122, Luoshi Road, Wuhan, 430070 China

**Keywords:** Chemistry, Materials science

## Abstract

Chalcogenide semiconductors and glasses have many applications in the civil and military fields, especially in relation to their electronic, optical and mechanical properties for energy conversion and in enviormental materials. However, they are much less systemically studied and their fundamental physical properties for a large class chalcogenide semiconductors are rather scattered and incomplete. Here, we present a detailed study using well defined first-principles calculations on the electronic structure, interatomic bonding, optical, and mechanical properties for 99 bulk chalcogenides including thirteen of these crytals which have never been calculated. Due to their unique composition and structures, these 99 bulk chalcogenides are divided into two main groups. The first group contains 54 quaternary crystals with the structure composition (A_2_BCQ_4_) (A = Ag, Cu; B = Zn, Cd, Hg, Mg, Sr, Ba; C = Si, Ge, Sn; Q = S, Se, Te), while the second group contains scattered ternary and quaternary chalcogenide crystals with a more diverse composition (A_x_B_y_C_z_Q_n_) (A = Ag, Cu, Ba, Cs, Li, Tl, K, Lu, Sr; B = Zn, Cd, Hg, Al, Ga, In, P, As, La, Lu, Pb, Cu, Ag; C = Si, Ge, Sn, As, Sb, Bi, Zr, Hf, Ga, In; Q = S, Se, Te; $\hbox {x} = 1$, 2, 3; $\hbox {y} = 0$, 1, 2, 5; $\hbox {z} = 0$, 1, 2 and $\hbox {n} = 3$, 4, 5, 6, 9). Moreover, the total bond order density (TBOD) is used as a single quantum mechanical metric to characterize the internal cohesion of these crystals enabling us to correlate them with the calculated properties, especially their mechanical properties. This work provides a very large database for bulk chalcogenides crucial for the future theoretical and experimental studies, opening opportunities for study the properties and potential application of a wide variety of chalcogenides.

## Introduction

In materials research, a large amount of efforts were related to energy conversion, mostly focusing in converting solar energy to other forms of energy such as electric, thermal, and chemical^1–6.^ Of particular interest are materials that can convert energy in the mid and far infrared (IR) range of radiation. Such materials can be used in a variety of civilian and military applications such as infrared sensors, optical communications, imaging technology and remote sensing^7–10^, atmospheric monitoring, radar and laser guidance^11^, laser frequency modulators^12^, and medical visualization^13^. The materials with narrow energy gaps and containing heavy elements that can be more effective in thermoelectric conversion are actively investigated^14–16^. In this regard, the ternary and quaternary chalcogenides play an important role because of their excellent performance for the above applications. There are many binary and ternary chalcogenide crystals can be easily doped by n-type elements or p-type elements to increase the conductivity in the thermoelectric applications^17^. Chalcogenides contain at least one of the chalcogen elements (S, Se, Te) and one or more electropositive (or electronegative in a few cases) elements. The electropositive elements are generally from group IB (Cu, Ag), IIB (Zn, Cd, Hg), IVA (Si, Ge, Sn), IIIA (In, Tl), IVB (Zr, Hf), IIA (Mg, Ba), and IA (Li, K, Cs) in the Periodic Table. In some cases, lanthanide elements such as La, Lu and othercan also be involved. This extremely diverse compositional space makes chalcogenide compounds a unique class of materials rarely seen in other materials classes such as semiconductors, large gap insulators, superconductors, silicates glasses, metallic alloys, etc. It is the formation of various possible chemical bonds between these elements that makes chalcogenides a special class of materials with diverse structures and properties. As a matter of fact, the energy band gaps for the 99 chalcogenide crystals reported in this paper range from 0.004 eV - 2.50 eV which makes them suitable for many aforementioned applications. Within the last decade, many of quaternary chalcogenide crystals, such as the A^I^–B^III^–C^IV^–X^VI^ system (where A^I^ = Cu, Ag; B^III^ = Ga, In; C^IV^ = Ge, Si; X^VI^ = S, Se), or A^I^_2_B^II^C^IV^Q_4_ system (where A^I^ = Cu, Ag; B^II^ = Mg, Mn, Fe, Zn, Cd, and Hg; C^IV^ = Si, Ge, Sn; and Q = S, Se, Te), have attracted a great deal of attention due to their compositional flexibility and functional turning ability that makes them ideal for thermoelectric devices^18–22^, optical devices^23^, nonlinear optical devices in the visible-infrared region and photovoltaic cells^3,24–30^, solar energy converters^31–34^, and magnetic applications^35^. Another group of chalcogenide crystals in the form of Ag_2_XYSe_4_ (X = Ba, Sr; Y = Ge, Sn) also become attractive because of their ability in converting heat energy to electricity^36^. Most recent studies show that quaternary chalcogenide crystals with compositions A^I^ B^II^C^IV^Q_4_ and A^I^_2_ B^II^C^IV^Q_5_ such as in BaZnSiSe_4_, SrCdGeSe_4_, and Ba_2_AsGaSe_5_ are crucial in medical and military applications due to their ideal infrared nonlinear optical (NLO) properties and high visible-light-induced photocatalytic reactivity^37–39^. Only a small fraction of such NLO materials with second harmonic generation (SHG) effect are commercially available. Materials such as Ag_2_BaGeS_4_, Ag_2_BaGeSe_4_, Ag_2_BaSnS_4_, and Ag_2_BaSnSe_4_ could be SHG active for different applications and they are now at the center of the chalcogenide research^40^. Cu_2_SrSiS_4_ and Cu_2_SrSnS_4_ show high LDT (laser damage threshold) and excellent SHG performance and that makes them promising materials for IR NLO applications^41,42^ . Unfortunately, there is a lack of comprehensive study on their electronic structure and physical properties. In the present work, we provide a comprehensive library of structural, electronic, optical and elastic properties for 99 chalcogenide crystals using first-principles calculations. They are divided into two groups: (A_2_BCQ_4_) (with A = Ag, Cu; B = Zn, Cd, Hg, Mg, Sr, Ba; C = Si, Ge, Sn; Q = S, Se, Te) and (A_x_B_y_C_z_Q_n_) (A = Ag, Cu, Ba, Cs, Li, Tl, K, Lu, Sr; B = Zn, Cd, Hg, Al, Ga, In, P, As, La, Lu, Pb, Cu, Ag; C = Si, Ge, Sn, As, Sb, Bi, Zr, Hf, Ga, In; Q = S, Se, Te) and ($\hbox {x}= 1$, 2, 3; $\hbox {y} = 0$, 1, 2, 5; $\hbox {z} = 0$, 1, 2, $\hbox {n}= 3$, 4, 5, 6, 9). The fully optimized structures are listed in Table S1 with the corresponding experimental lattice parameters. Such a comprehensive study enables us to make important correlations among different properties in getting a broader view. In particular, we advocate the use of a single quantum mechanical metric that describes the strength and the internal cohesion of the crystal, the total bond order density (TBOD). In the following section, we briefly describe the computational methods which were used to do the calculations, followed by the results and discussions section. Focus will be on the optical, bonding, and mechanical properties, and their correlations with the TBOD. We end up with a brief conclusion and our vision for the future study of chalcogenide crystals and glasses.

## Methods

In this work, two well-defined density functional theory (DFT) based methods were used: the Vienna Ab initio Simulation Package (VASP)^43,44^ and the orthogonal linear combination of atomic orbitals (OLCAO) method^45^. VASP was used to optimize the crystal structures and to calculate the mechanical properties. The Perdew-Burke-Ernzerhof (PBE) generalized-gradient-approximation (GGA)^46^ was used for the exchange and correlation potential in solving the Kohn-Sham equation with an energy cut-off of 500 eV. This energy cut-off has been carefully tested for all crystals and it is found to be the best choice balancing the fast convergence and accurate ground state energy. For the VASP calculation, the Monkhorst scheme^47^ with different k-point mesh ranging from $9 \times 9 \times 5$ and $5 \times 9 \times 5$ was used for Ba_4_AgInS_6_ (48 atoms) and Ag_2_In_2_SiSe_6_ (44 atoms) respectively to $8 \times 9 \times 9$ for medium sized crystals such as Cu_2_ZnSiSe_4_ (16 atoms). The electronic and ionic force convergence criteria were set at $10^{-6}$ eV and $10^{-4}$ eV/Å respectively. The VASP optimized structures for all crystals were used as input to calculate the electronic structure, interatomic bonding, and optical properties using the OLCAO method with different choice of basis size for each atom in the database^45^. The use of localized atomic orbitals in the basis expansion in contrast to the plane-wave expansion is particularly effective for calculating the electronic structure and interatomic bonding for both crystalline^48–52^ and non-crystalline materials^53,54^ especially those with complex structures typical in the biomolecular systems^55,56^. In the OLCAO calculation, a sufficient number of k-points mesh were used for band structure and density of states (DOS) calculations based on the size of crystal. For interatomic bonding, a more localized minimal basis (MB) set is used based on Mulliken scheme^57^. Equations (1) and (2) show the formulae for effective charge($Q_\alpha ^*$) and bond order (BO) values, or the overlap population $\rho _{\alpha \beta }$ between any pair of atoms ($\alpha ,\beta$).

1 $$\begin{aligned} Q_\alpha ^*= & {} \sum _{i}\sum _{m,occ}\sum _{j,\beta } C_{i\alpha }^{*m} C_{j\beta }^m S_{i\alpha ,j\beta } \end{aligned}$$

2 $$\begin{aligned} \rho _{\alpha \beta }= & {} \sum _{m,occ}\sum _{j,\beta } C_{i\alpha }^{*m} C_{j\beta }^m S_{i\alpha ,j\beta } \end{aligned}$$

In the above equation, $S_{i\alpha ,j\beta }$ are the overlap integrals between the ith orbital in $\alpha$th atom and the *j*th orbital in $\beta$th atom. $C_{j\beta }^m$ is the eigenvector coefficients of the *m*th occupied band. The BO from Eq. (2) defines the relative strength of the bond. The summation of all BO values in the crystal gives the total bond order (TBO). When normalized by the cell volume, we obtain the total bond order density (TBOD). TBOD is a single quantum mechanical metric to describe the internal cohesion of the crystal^58^. It can be conveniently decomposed into partial components or the partial bond order density (PBOD) for any structural units or groups of bonded atoms. The details for the calculation of interatomic bonding, optical, and mechanical properties are described in the Sects. 1 and 2 in the Supplementary Information (SI). The orthogonalized linear combination of atomic orbitals (OLCAO) method was used to calculate the electronic structures and partial charge distributions of the chalcogenide crystals under study. The OLCAO method is an all-electron method based on the local density approximation of DFT. It uses the atomic orbitals expanded in Gaussian-type orbitals (GTO) in the basis expansion. This method is particularly efficient for calculating the electronic structure of different systems. In the present calculation, a full basis set, which consisted of the core orbitals, occupied valence orbitals, and the next empty shell of unoccupied orbitals for each atom, was used for the self-consistent potential and the density of states (DOS) calculations. The more localized minimal basis was used for partial charges calculation under the Mulliken scheme. The partial charge $\Delta Q$ on each atom is defined as the deviation of charge from the calculated effective charge $Q^*$ from the neutral atom charge ($Q_0$) or $\Delta Q = Q_0-Q^*$. So negative $\delta Q$ implies gain of electrons or electronegative and positive $\Delta Q$ means loss of electron or electropositive.

## Results and discussion

Table S1 in the SI lists the 99 quaternary crystals of the first group (A_2_BCQ_4_) (1–54) and the second group (A_x_B_y_C_z_Q_n_) (55–99). The data presented includes the name of the crystal, its space group, the VASP relaxed crystal parameters, and the available experimental parameters with appropriate references. In all subsequent discussions, the same specific order and the ID number labeled for the crystal are maintained to avoid any confusion. To make it easier for the readers to identify the specific crystal, we add the ID number in front of the crystal name most of the time. In both groups, we distinguish the chalcogen elements by referring them as Q, and the other elements by A, B, and C. Any exceptions will be specifically mentioned.

### Electronic structure

**Figure 1:**
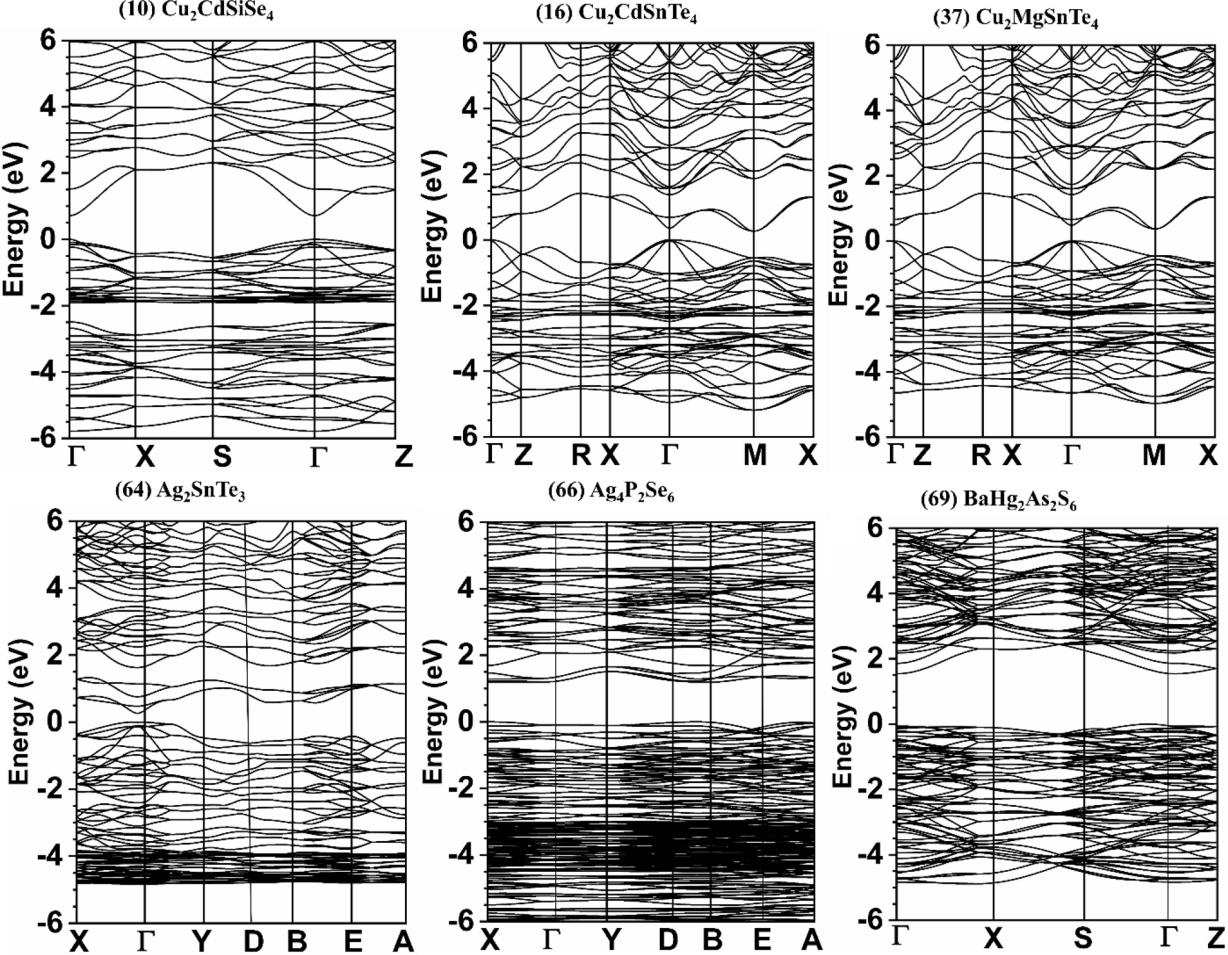
Calculated band structure of: Cu_2_CdSiSe_4_, Cu_2_CdSnTe_4_, Cu_2_MgSnTe_4_, Ag_2_SnTe_3_, Ag_4_P_2_Se_6_, BaHg_2_As_2_S_6_.

**Figure 2:**
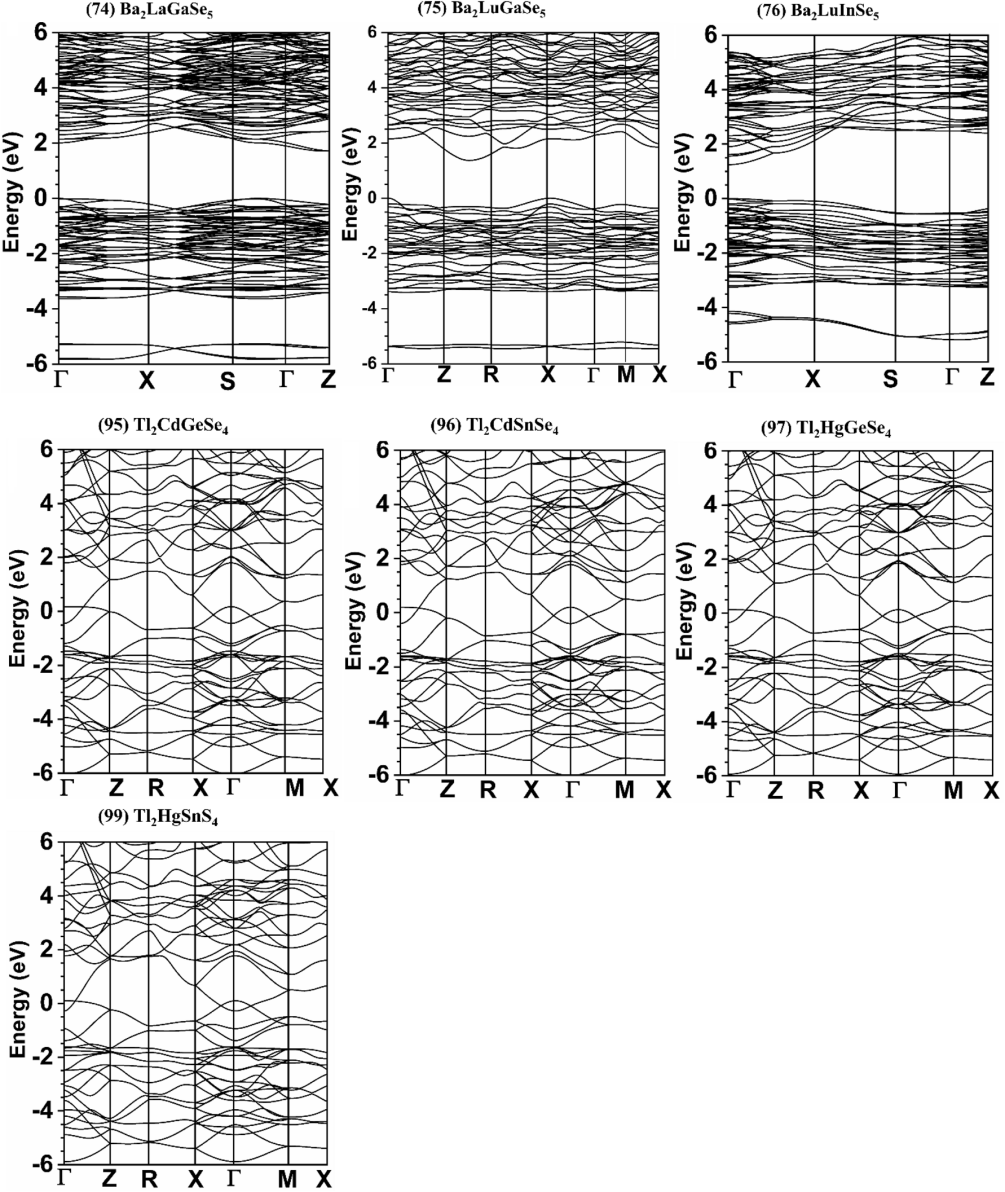
Calculated band structure of: Ba_2_LaGaSe_5_, Ba_2_LuGaSe_5_, Ba_2_LuInSe_5_, Tl_2_CdGeSe_4_, Tl_2_CdSnSe_4_, Tl_2_HgGeSe_4_, and Tl_2_HgSnS_4_.

**Figure 3:**
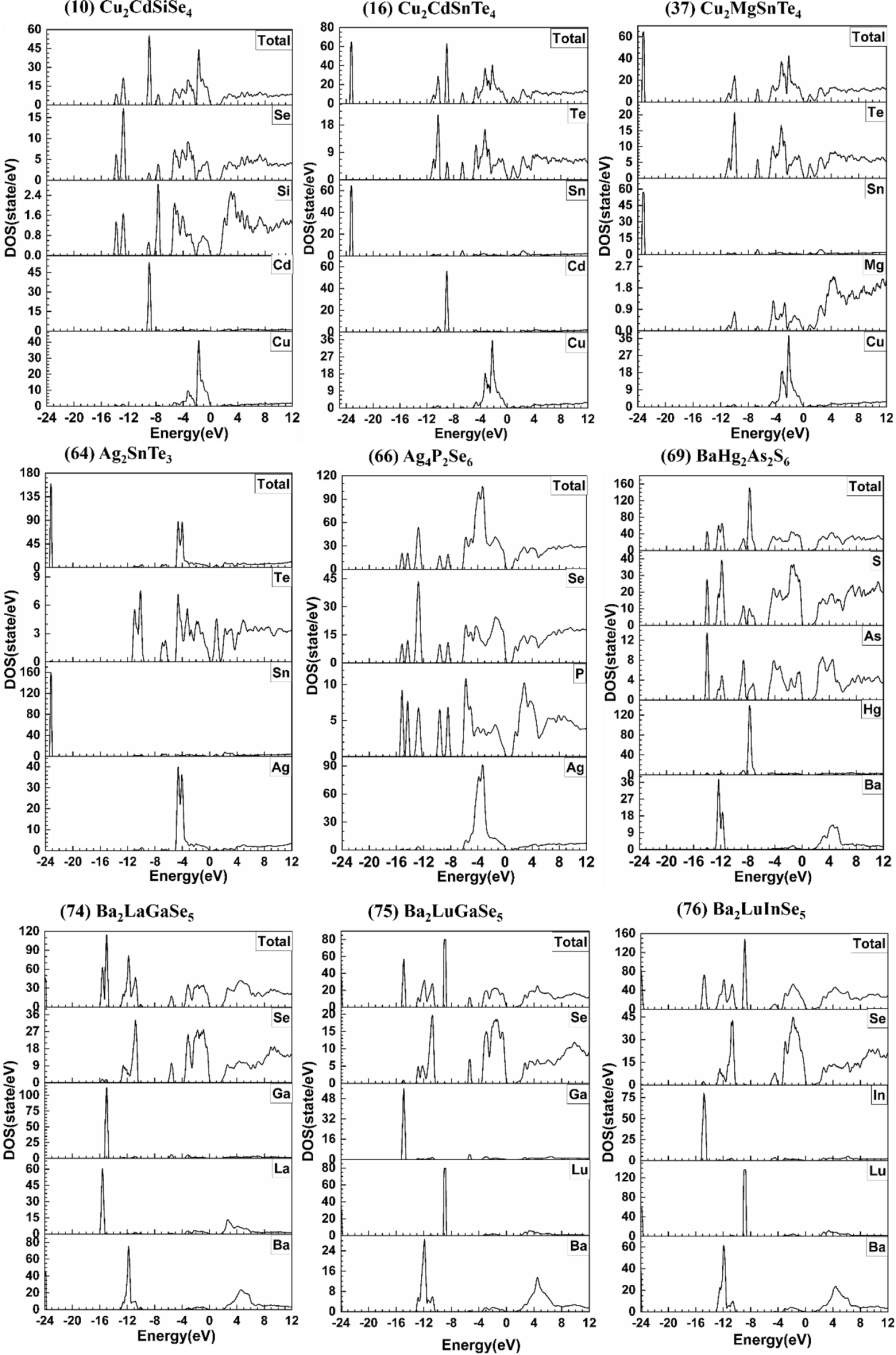
Calculated DOS and PDOS of: Cu_2_CdSiSe_4_, Cu_2_CdSnTe_4_, Cu_2_MgSnTe_4_, Ag_2_SnTe_3_, Ag_4_P_2_Se_6_, BaHg_2_As_2_S_6_, Ba_2_LaGaSe_5_, Ba_2_LuGaSe_5_, Ba_2_LuInSe_5_.

**Figure 4:**
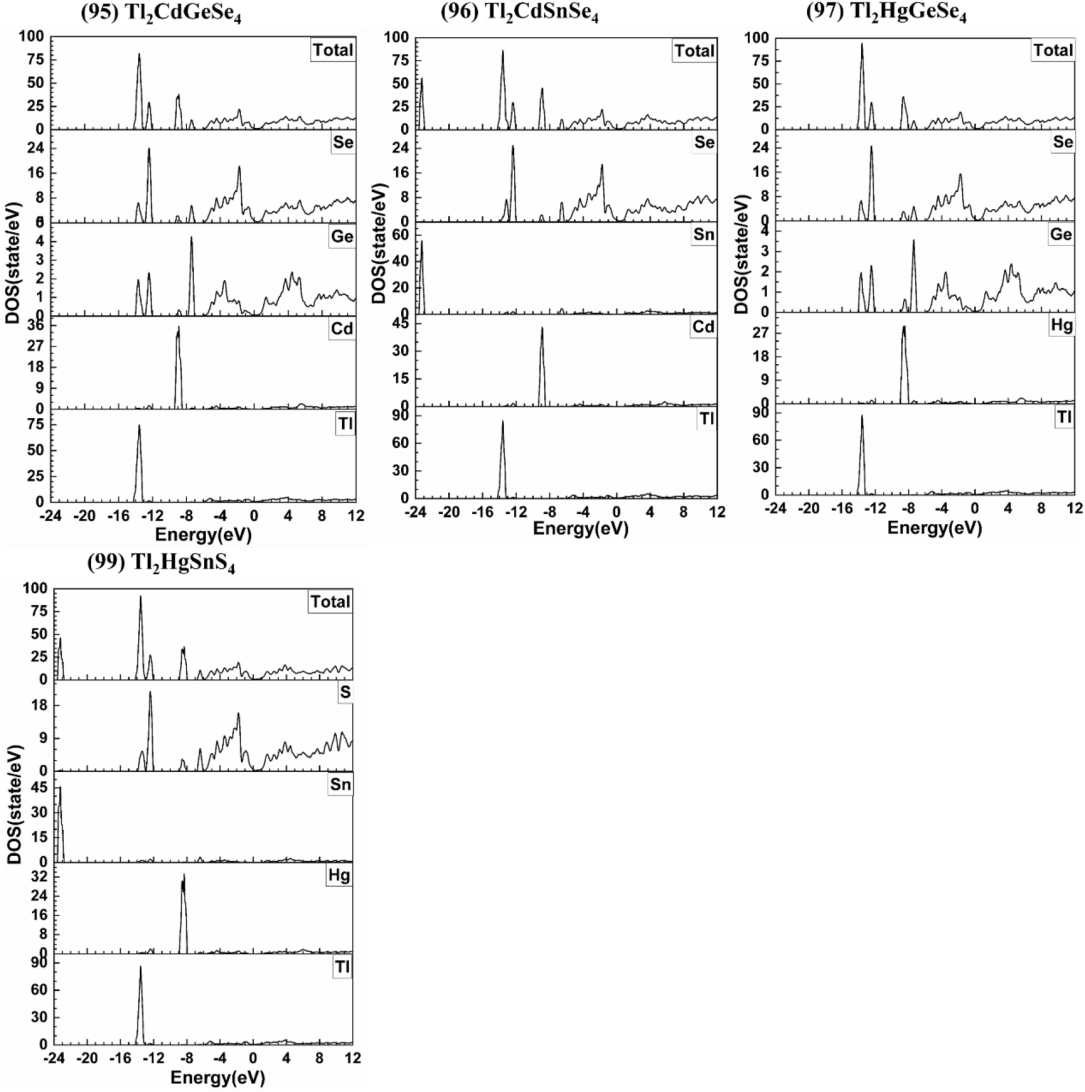
Calculated DOS and PDOS of Tl_2_CdGeSe_4_, Tl_2_CdSnSe_4_, Tl_2_HgGeSe_4_, and Tl_2_HgSnS_4_.

The most important quantity in a crystal to understand its physical and chemical properties is its electronic structure. The calculated results for the band structures and the density of states (DOS) for the 99 chalcogenide crystals are shown in Figs. S1 and S2 respectively. Table S2 shows the comparison between our calculated direct (D) and indirect (ID) band gaps (Eg), and the experimentally data or other theoretical calculations with references cited for these 99 crystals. Among them, 67-BaZnSiSe_4_ has the largest Eg of 2.494 eV, whereas 18-Cu_2_HgGeSe_4_ has the smallest Eg of 0.005 eV. The crystals 21-Cu_2_HgSnSe_4_, 62-Cu_2_GeSe_3_, 95-Tl_2_CdGeSe_4_, 96-Tl_2_CdSnSe_4_, 97-Tl_2_HgGeSe_4_, 98-Tl_2_HgSiSe_4_, and 99-Tl_2_HgSnS_4_ are metals or semi-metals with zero band gaps. We have identified the following 13 crystals for specific focused discussion: 10-Cu_2_CdSiSe_4_, 16-Cu_2_CdSnTe_4_, 37-Cu_2_MgSnTe_4_, 64-Ag_2_SnTe_3_, 66-Ag_4_P_2_Se_6_, 69-BaHg_2_As_2_S_6_, 74-Ba_2_LaGaSe_5_, 75-Ba_2_LuGaSe_5_, 76-Ba_2_LuInSe_5_, 95-Tl_2_CdGeSe_4_, 96-Tl_2_CdSnSe_4_, 97-Tl_2_HgGeSe_4_, and 99-Tl_2_HgSnS_4_. They are marked bold in Table S2. These crystals are the first-time theoretical studied for their electronic structure, optical, and mechanical properties and some of them have unique properties mentioned above. They are shown in Figs. 1, 2, 3, and 4, respectively. As can be seen, some are semiconductors or insulators (10-Cu_2_CdSiSe_4_, 16-Cu_2_CdSnTe_4_, 37-Cu_2_MgSnTe_4_, 64-Ag_2_SnTe_3_, 66-Ag_4_P_2_Se_6_, 69-BaHg_2_As_2_S_6_, 74-Ba_2_LaGaSe_5_, 75-Ba_2_LuGaSe_5_, and 76-Ba_2_LuInSe_5_) and the others are actually metals with no band gaps (95-Tl_2_CdGeSe_4_, 96-Tl_2_CdSnSe_4_, 97-Tl_2_HgGeSe_4_, and 99-Tl_2_HgSnS_4_).

### Partial charge

An important electronic structure property in a crystal is the distribution of the partial charge (PC) on each atom in the crystal. PC is the deviation of the effective charge $Q^*$ from the neutral atom charge $Q_0$ or simply the charge transfer. The calculated $Q^*$ on each atom in the 99 chalcogenide crystals is listed in column 3 of Table S3. The generally accepted concept is that chalcogen elements (S, Se, Te) receive electrons from the other atoms in the chalcogenide compounds or they are electronegative. The other atoms in a chalcogenide compound (except for Cu and Ag in most cases) lose charge and they are mostly electropositive. It turns out that this generally accepted notion is grossly over-simplified, and the real situation is far more complicated and subtle. With 99 chalcogenide crystals formed by 30 elements, we are in the unique position to analyze the PC distribution and charge transfer mechanism in much greater detail than for just one or a few crystals. In the first group with the structure (A_2_BCQ_4_), the PC values for most of A elements are negative similar to the chalcogen elements except for some crystals such as 36-Ag_2_ZnSiS_4_, 38-Ag_2_ZnSnSe_4_, and 39-Ag_2_CdGeS_4_ where the A element (Ag) loses charge. While the B and C elements have positive PC. To explain why Cu and Ag are electronegative in most of the crystals in group one, we must dig deeper. Cu and Ag are noble metals with full shell of 10 3d and 10 4d electrons respectively, and metals usually lose charge to the other elements in the chemical compound, specially to the nonmetals elements, but our calculations for the partial charge and effective charge indicate that these two elements (Cu, Ag) can gain or loss charge depending on chalcogen elements their interact. First, for crystals that contain Cu and Ag with the nonmetal chalcogen elements (S, Se) and later with Te (a metalloid element) which is very different than S and Se. The bonding properties indicate that Cu and Ag have two types of bonds. depending on the chalcogen elements (S or Se). They form bonds that are ionic bonds (metal-nonmetal bond), and between two metal elements (Cu, Ag). Cu and Ag lose charge to the nonmetallic chalcogen elements S and Se and these bonds occur at shorter bond lengths (1.5–2.0 Å). The other type of bonds is between Cu or Ag and with one of the metal elements Zn and Cd and with one of the alkaline earth elements (Mg, Sr, Ba). These bonds occur at long bond lengths ($> 3.8\, \AA$). In this type of complex bonding, Cu and Ag can attract or pull the charge from Zn, Cd, Mg, Sr, and Ba elements since Cu and Ag have much higher electronegativity. Our calculations show that under these scenario, Cu and Ag are electronegative. On the other hand, the Cu–Te and Ag–Te bonds are much more complicated because Te is metalloid element. It sometimes acts like metal element and lose charge and sometimes behaves like a nonmental element and gain charge. In the second group (A_x_B_y_C_z_Q_n_), almost all A, B, and C elements (Ag, Cu, Zn, Cd, Si, Sn, Ge, In, P, Al, Sb, Ga, Bi, La, Lu, Cs, K, Li, Sr) lose charges to the chalcogen elements, except for Ba, which gains charges. To the best of our knowledge, this new conspicuous property on complex chalcogenides has not been reported anywhere in either computational or experimental studies. The above results on PC distribution are summarized in Figs. S3, S4 and S5. Because of the large number on the non-chalcogen elements in the 99 crystals, the calculation of their PC for each element from each crystal is a monumental task but could be extremely revealing. For the benefit of easier presentation, we split the plot for the non-chalcogen elements in two figures Fig. S3 with 13 elements (Cu, Ag, Zn, Cd, Hg, Al, Ga, In, Tl, Si, Ge, Sn, Pb) and Fig. S4 with 14 elements (P, As, Sb, Bi, Li, K, Cs, Mg, Sr, Ba, Zr, Hf, La, Lu) while keep the display for the three chalcogen elements (S, Se, Te) in Fig. S5. The horizontal bar in each column indicate the averaged PC for that element. Figure S3 shows the PC distribution of the first 13 elements. Depending on the crystals they originate, they generally have a wide range of scatted values and only Cu has an average negative PC of − 0.104 e$^{-}$ and Ag has a slightly positive average charge of 0.026 e$^{-}$. From Zn to Pb, the average PC range from 0.310 e$^{-}$ in Zn to 0.811 e$^{-}$ in Cd with most of them having close average values. An important facet is a wide scattering of PC values for individual crystals indicating the averaged PC value for any element in chalcogenide crystals has no real meaning since it depends on the crystal components and the overall charge transfers between them. The situation is slightly different in Fig. S4 for the 14 elements from P to Lu mainly because there are much less crystals involving these group of elements. One of the spectacular outliers of the rare earth element La from a single crystal 74-Ba_2_LaGaSe_5_. Other outliers include Ba which consists of 11 crystal distinctively separated into 2 groups with average PC of 1.729 e$^{-}$ between them. The most important PC information is the distribution of the three chalcogen elements in Fig. S5.

The range from − 1.255 e$^{-}$ to − 0.216 e$^{-}$ in S, − 1.018 e$^{-}$ to − 0.125 e$^{-}$ in Se, and − 0.247 e$^{-}$ to + 0.083 e$^{-}$ in Te with average PC are − 0.475 e$^{-}$, − 0.437 e$^{-}$ and − 0.064 e$^{-}$ respectively. In the sulfides, 77-Ba_4_AgInS_6_ (PC = − 1.255 e$^{-}$) and 71-Ba_2_AlSbS_5_ (PC = − 1.033 e$^{-}$) are far more negative than the other crystals. The selenides have a slightly less negative PC than the sulfides with three of them more negative than the rest. They are (74-Ba_2_LaGaSe_5_, 72-Ba_2_GaBiSe_5_ and 73-Ba_2_AsGaSe_5_ with PC of − 1.018 e$^{-}$, − 0.919 e$^{-}$ and − 0.867 e$^{-}$ respectively. On the other hand, for the tellurides, four crystals (19-Cu_2_MgGeTe_4_, 3-Cu_2_ZnSiTe_4_, 22-Cu_2_HgSnTe_4_, 8-Cu_2_ZnSnTe_4_) actually have positive PCs of 0.083 e$^{-}$. 0.062 e$^{-}$, 0.020 e$^{-}$ and 0.007 e$^{-}$ respectively. The spectacular nature of the diverse distribution of PC in all the elements in the 99 complex bulk chalcogenide compounds is remarkable and each data point for the PC of each atom can be traced to the specific crystal among the 99 crystals.

### Interatomic bonding

**Figure 5:**
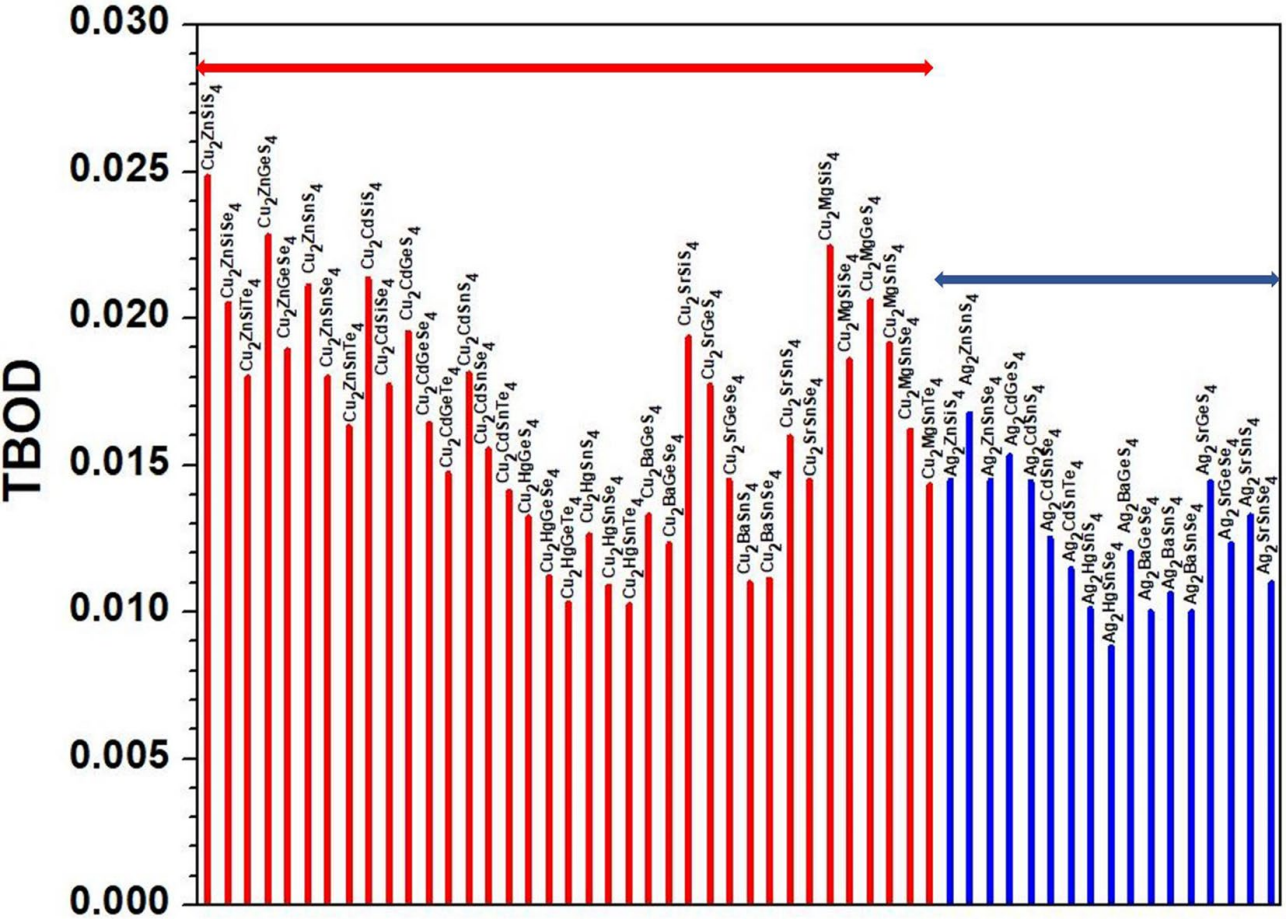
Distribution of the calculated TBOD for the 54 crystals (first group).

**Figure 6:**
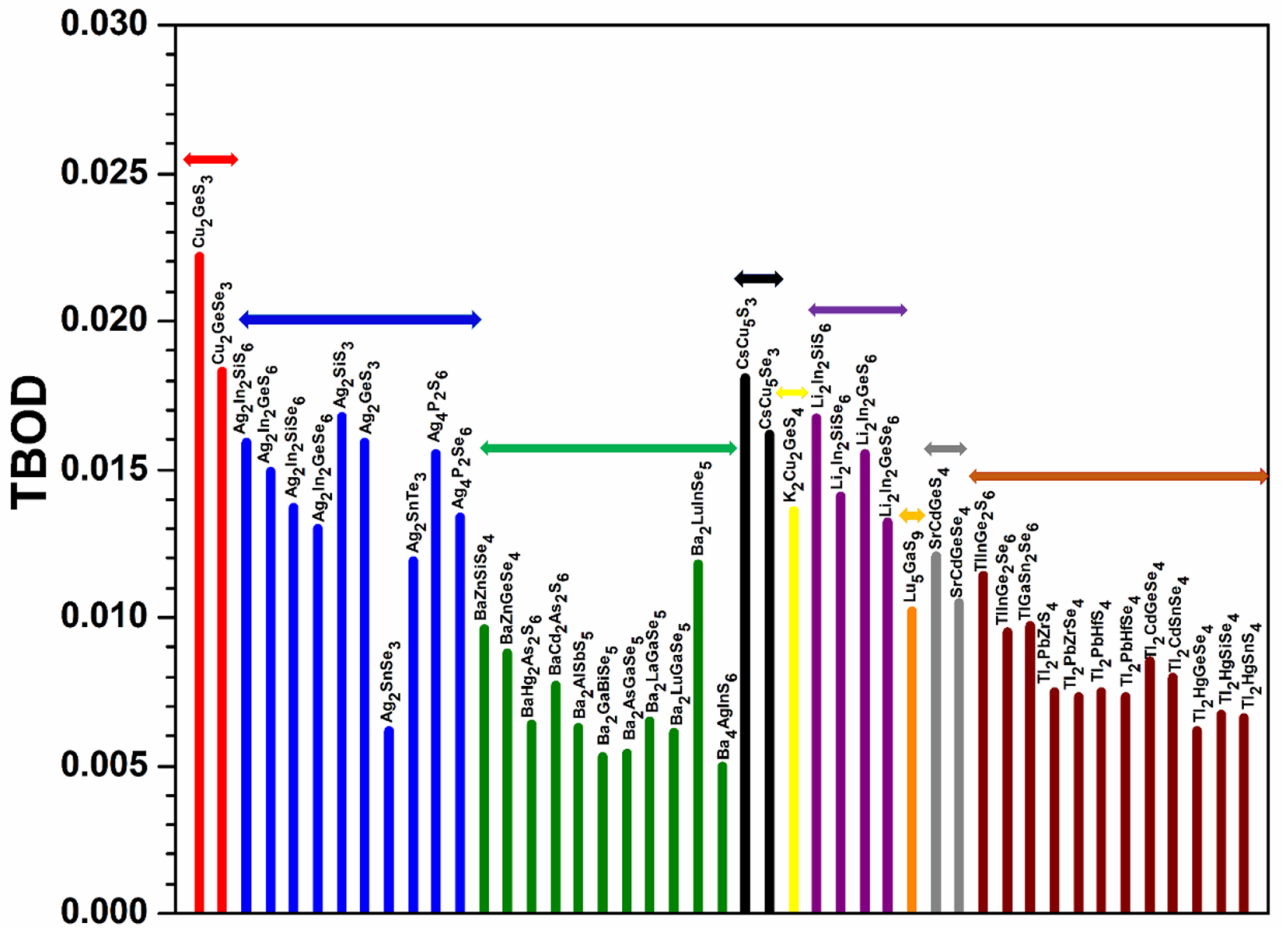
Distribution of the calculated TBOD for the 45 crystals (second group).

An important characteristics is to investigate the interatomic bonding between every pair of atoms in the crystal represented by the BO value that quantifies the strength of the bond^58^. These data can then be used to obtain the key parameter TBOD discussed in the method section. The highest TBOD among these 99 crystals is in 1-Cu_2_ZnSiS_4_ followed by 32-Cu_2_MgSiS_4_, while the lowest TBOD is from 77-Ba_4_AgInS_6_ closely followed by 72-Ba_2_GaBiSe_5_ (see Table S3). In Figs. 5 and 6, we display the TBOD for the 99 crystals in 2 main groups respectively. The first group with 54 crystals (A_2_BCQ_4_), is divided into two subgroups (Cu-related and Ag-related) indicated with horizontal bars in Fig. 5, while the second group (A_x_B_y_C_z_Q_n_) with 45 crystals in Fig. 6 is divided into 9 subgroups indicated by horizontal bars which are related to the elements A= (Cu, Ag, Ba, Cs, K, Li, Lu, Sr, Tl) respectively. It can be seen that the first main group A_2_BCQ_4_ have relatively higher TBOD values than the second main group (A_x_B_y_C_z_Q_n_). In Fig. 5 for the first main group, crystals with A = Cu, Ag; B = Hg; C = Sn; Q = Se, Te have the lowest TBOD. The data in Fig. 6 for the second group are more scattered. However, we can still observe that the Cu-related crystals have the highest TBOD whereas the Ba-related crystals have the lowest TBOD. The Ag-related, Cs-related, and Li-related crystals all have very close TBOD values. An important observation from the TBOD calculations is that in moving from S to Se to Te through these 99 crystals, the TBOD value decreases. The possible reason has to do with the size of the atoms which affects the volume of the crystal resulting in longer bond lengths hence smaller TBOD. That being said, we cannot say that the chalcogen elements completely control the TBOD value of the crystal because the elements A, B and to a lesser extend C also play a role in determining their total bond order and the volume of the crystal, hence the TBOD. We need to reiterate that the use of the novel concept of TBOD is extremely helpful to extract the internal cohesion of otherwise very complex crystals of chalcogenide compounds.

**Figure 7:**
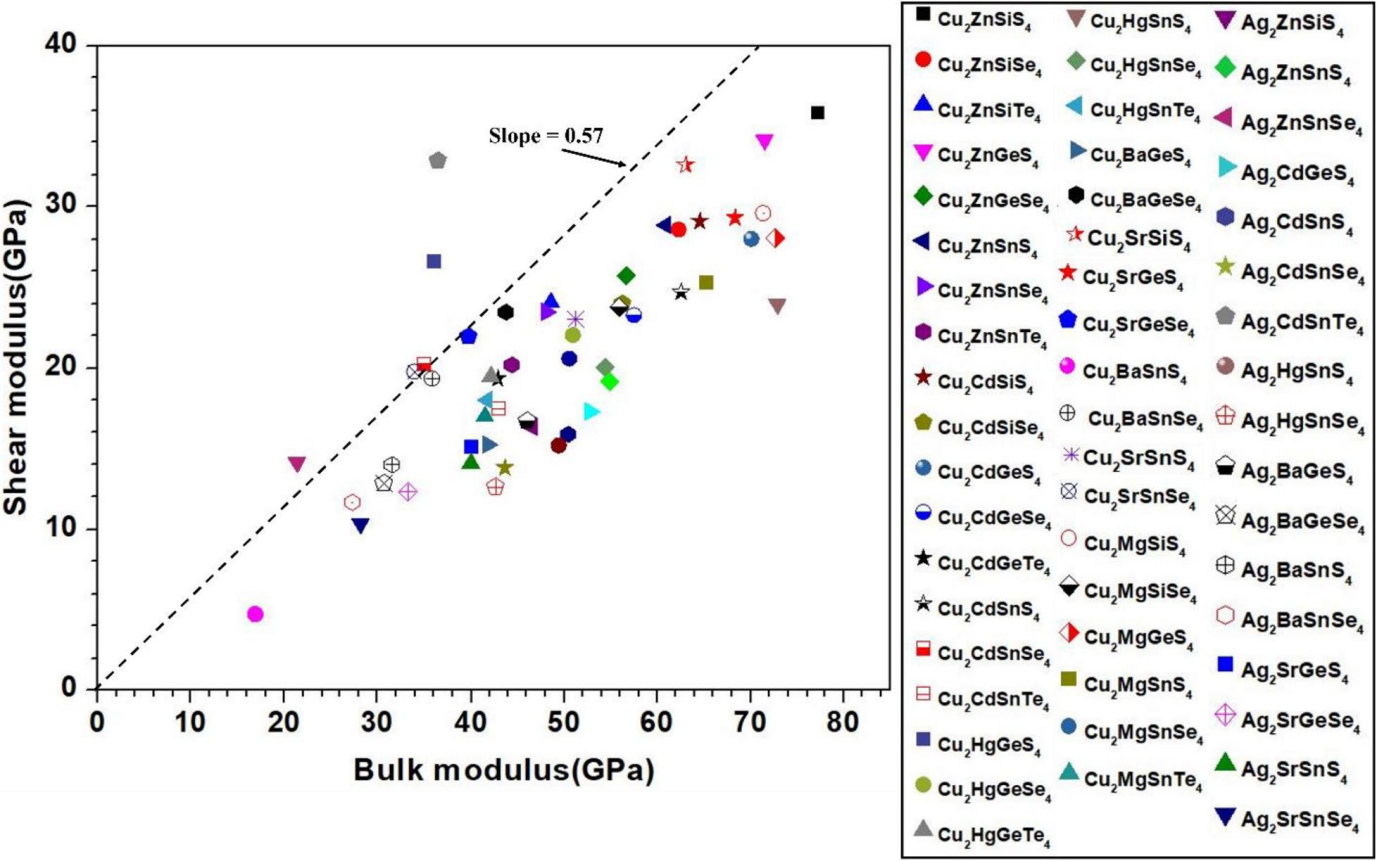
Bulk modulus versus shear modulus for the first group (54 crystals) of crystals.

### Optical properties

The optical properties of these 99 chalcogenide crystals are particularly important because of their many optical applications. They can be calculated relatively easily within the one-electron random phase approximation using the OLCAO method. It starts with the calculations of the imaginary part of the dielectric function $\epsilon _2(\omega )$ from the interband optical transition with explicit inclusion of the dipole matrix elements. The real part of the dielectric function $\epsilon _1(\omega )$ is obtained from $\epsilon _2(\omega )$ by applying Kramers-Kronig transformation. The refractive index *n* of the crystal is obtained as the square root of $\epsilon _1(\omega )$ in the zero-frequency limit, or $n=\sqrt{\epsilon _1(0)}$. More details can be found in Sect. 1 in SI including the calculation of the energy loss function (ELF) and the evaluation of the Plasmon frequency $\omega _p$. The calculated real and imaginary parts of the dielectric functions for these 99 crystals are shown in Fig. S6, and the refractive indices n are listed in Table S4. As can be seen, each crystal has its unique absorption features or interesting optical properties. We selectively discuss some of them in A_2_BCQ_4_ (with A = Ag, Cu; B = Sr, Ba; C = Ge, Sn; Q = S, Se) and also the following Ba-related and Sr related crystals (71-Ba_2_AlSbS_5_, 72-Ba_2_GaBiSe_5_, 73-Ba_2_AsGaSe_5_, 74-Ba_2_LaGaSe_5_, 75-Ba_2_LuGaSe_5_, 76-Ba_2_LuInSe_5_, 77-Ba_4_AgInS_6_, 86-SrCdGeS_4_, 87-SrCdGeSe_4_). They are part of the results shown in Fig. S6. The general feature for these selected crystals is that $\epsilon _1(\omega )$ reaches a peak at certain energy and then starts decreasing, eventually reaching a negative value. It then increases again before finally leveling off to zero value again. Since the crossing at zero value is generally associated with Plasma excitation, this implies that in these group of selected crystal, they may have two plasma excitations and two Plasma frequencies, such as that reported recently in Ref.^59^. This unique feature is not specifically related to the structure of the crystals (A_2_BCQ_4_ or A_x_B_y_C_z_Q_n_), but strongly related to the presence of Ba and Sr in these crystals. This is an important observation revealed in the present study which clearly originate from the electronic structure. From the dielectric function, we can obtain the ELF for all the 99 chalcogenides which are shown in Fig. S7. The ELF is an important part of the optical properties of crystals and represents the collective excitation of excited electrons at high frequency. The position of its main peak is defined as the Plasmon frequency $\omega _p$ which usually occurs at the frequency when the real part of dielectric function vanishes. Below $\omega _p$, the incident waves will be mostly reflected. The $\omega _p$ values for the 99 crystals are listed in Table S3. The highest $\omega _p$ is identified to be in 85-Lu_5_GaS_9_ at 31.10 eV, while the lowest $\omega _p$ is in 63-Ag_2_SnSe_3_ at 15.50 eV.

### Mechanical properties

**Figure 8:**
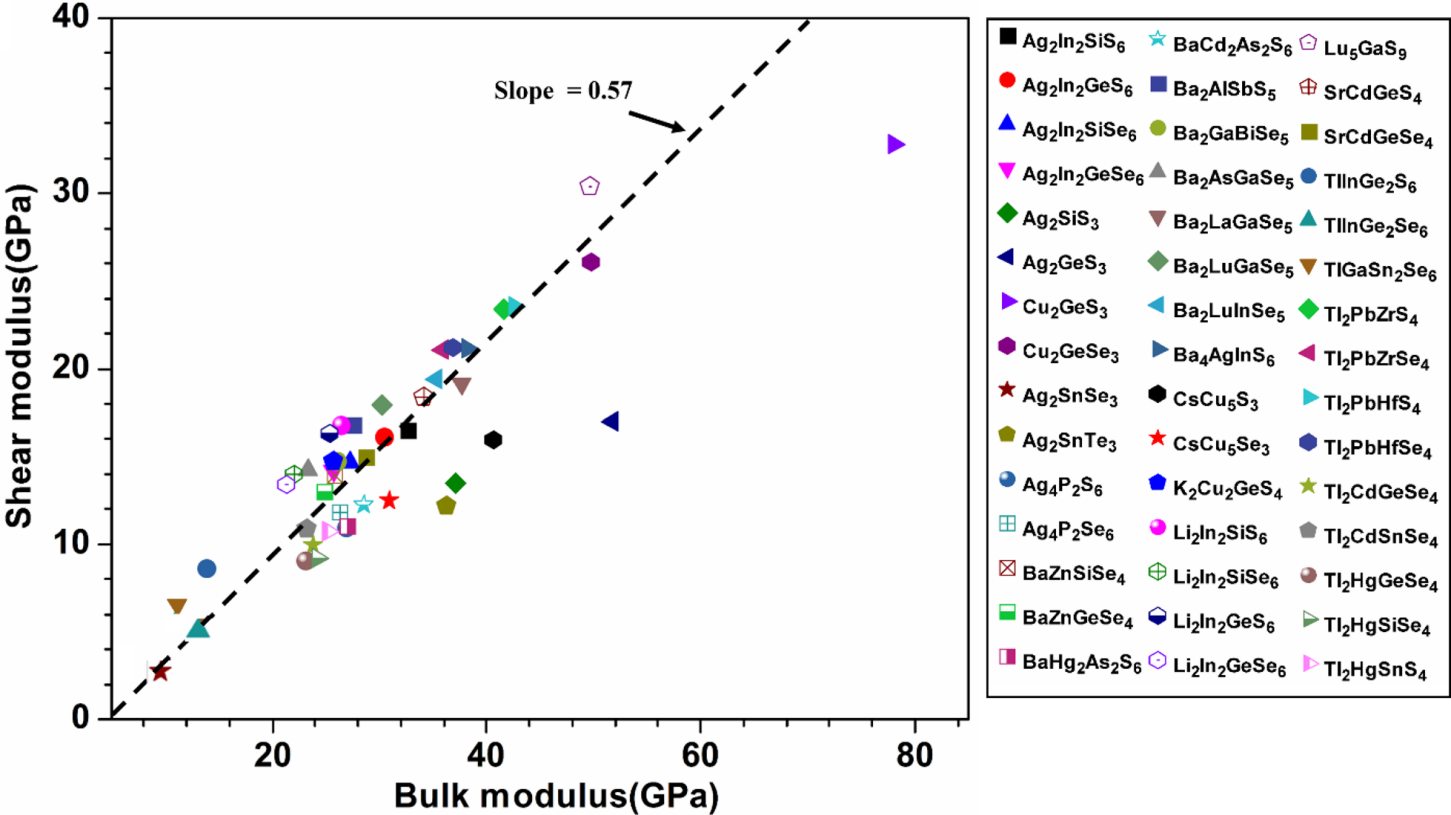
Bulk modulus versus shear modulus for the second group (45 crystals) of crystals.

Mechanical properties of chalcogenide materials are much less studied compared to optical properties even though they are critical in many practical applications^60,61^. In this work, we fill this void by calculating the mechanical properties of the 99 chalcogenides using the method described in Sect. 2 in SI. The calculated elastic constants are listed in Table S5 and the mechanical parameters derived from them are listed in Table S6. The principal coefficients C_11_, C_22_, and C_33_, related to the unidirectional compression along the principal x, y and z directions, reflect the isotropic elasticity of the crystals and they are usually close in cubic crystals with high symmetry^62^. Equivalently, we say C_11_, C_22_, and C_33_ reflect the resistance to the deformation of the crystal along x, y, and z directions. The elastic constants depend on the crystals structure (lattice parameters) and the strength of the bonds between the elements in the crystal. Table S5 shows that some of the crystals have C_22_ higher than C_11_ and C_33_ which simply suggest that they are more compressible along x- and z-axes than along y-axis. We note that the two crystals 38-Ag_2_ZnSiS_4_ and 63-Ag_2_SnSe_3_ have negative elastic constants which implies a negative stiffness. This behavior could be related to a material instability^63^. To the best of our knowledge, this is the first time that this observation is reported for these two crystals either experimentally or computationally. From the elastic coefficient Cij and the compliance tensor Sij (Sij = 1/Cij), the mechanical parameters bulk modulus (K), shear modulus (G), Young’s modulus (E), and Poisson’s ratio ($\eta$) can be obtained using Voigt- Reuss-Hill (VRH) approximation for polycrystals as explained in SI^64–66^. They are listed in Table S6. Another useful parameter is G/K or Pugh’s modulus ratio^67,68^. According to Pugh’s criterion, crystals with G/K larger than 0.57 are brittle, and those with less than 0.57 tend to be more ductile^68–71^. Figure 7 shows the scattered plot of G versus K for the 54 crystals in the first main group, while Fig. 8 for the 45 crystals in the second main group. In both plots, we draw a dashed line with slope of 0.57. For the first group, most crystals tend to be ductile except 44-Ag_2_CdSnTe_4_, 38-Ag_2_ZnSiS_4_, and 17-Cu_2_HgGeS_4_. In the second group, the data can be divided roughly into three subgroups. The first subgroup have G/K values higher than 0.57 (71-Ba_2_AlSbS_5_, 73-Ba_2_AsGaSe_5_, 75-Ba_2_LuGaSe_5_, 80-K_2_Cu_2_GeS_4_, 81-Li_2_In_2_SiS_6_, 82-Li_2_In_2_SiSe_6_, 83-Li_2_In_2_GeS_6_, 84-Li_2_In_2_GeSe_6_, 85-Lu_5_GaS_9_, 88-TlInGe_2_S_6_, 90-TlGaSn_2_Se_6_, 92-Tl_2_PbZrSe_4_, and 94-Tl_2_PbHfSe_4_) so they tend to be brittle. The second subgroup has data lie very close or on the dashed line (56-Ag_2_In_2_GeS_6_, 57-Ag_2_In_2_SiSe_6_, 58-Ag_2_In_2_GeSe_6_, 67-BaZnSiSe_4_, 68-BaZnGeSe_4_, 72-Ba_2_GaBiSe_5_, 74-Ba_2_LaGaSe_5_, 76-Ba_2_LuInSe_5_, 77-Ba_4_AgInS_6_, 86-SrCdGeS_4_, 87-SrCdGeSe_4_, 89-TlInGe_2_Se_6_, 91-Tl_2_PbZrS_4_, and 93-Tl_2_PbHfS_4_). In the third subgroup, the remaining 18 data points are located under the dashed line, so these crystals tend to be more ductile.

**Figure 9:**
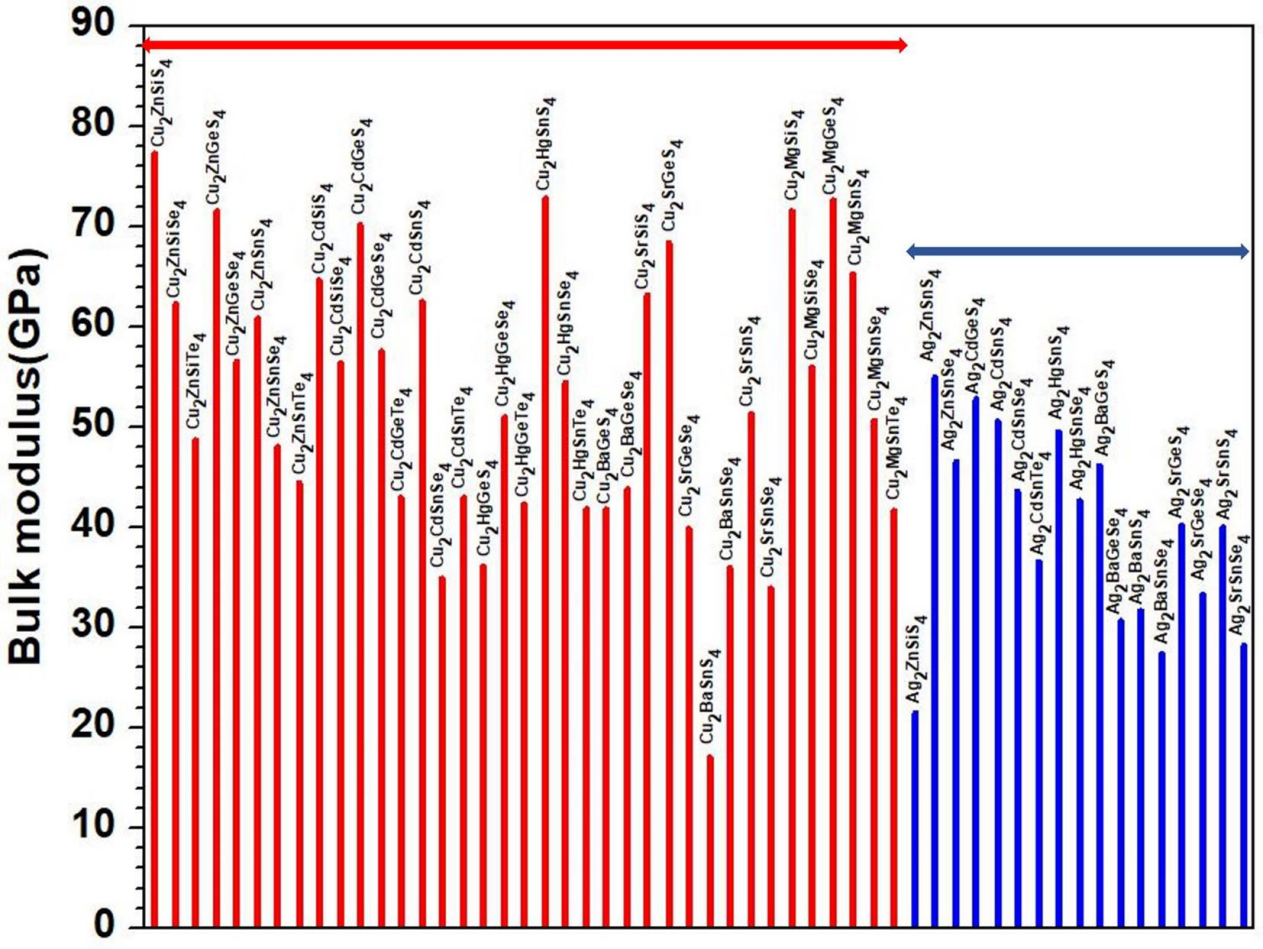
Distribution of bulk modulus for the first group (54 crystals).

**Figure 10:**
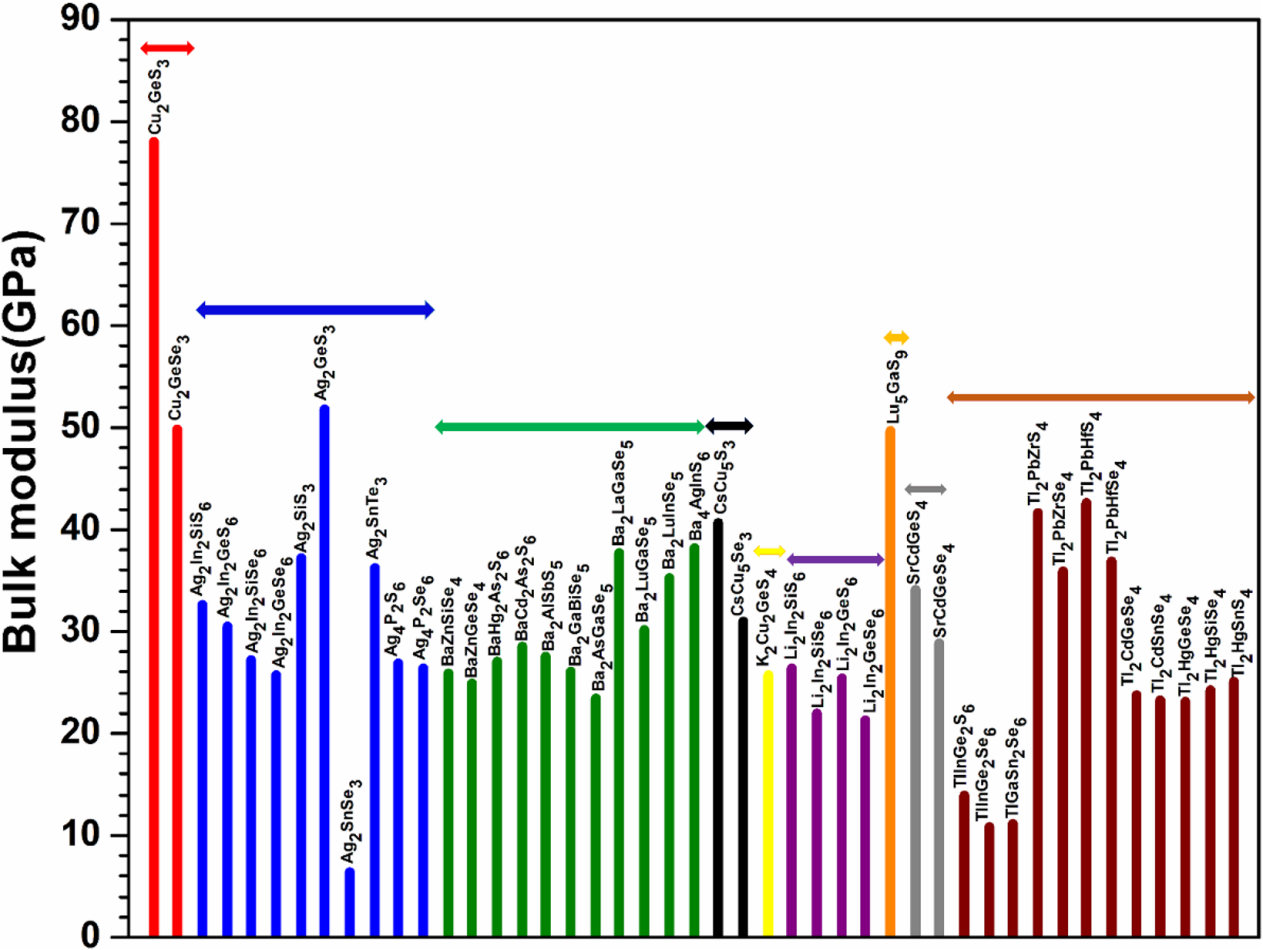
Distribution of bulk modulus for the second group (45 crystals).

**Figure 11:**
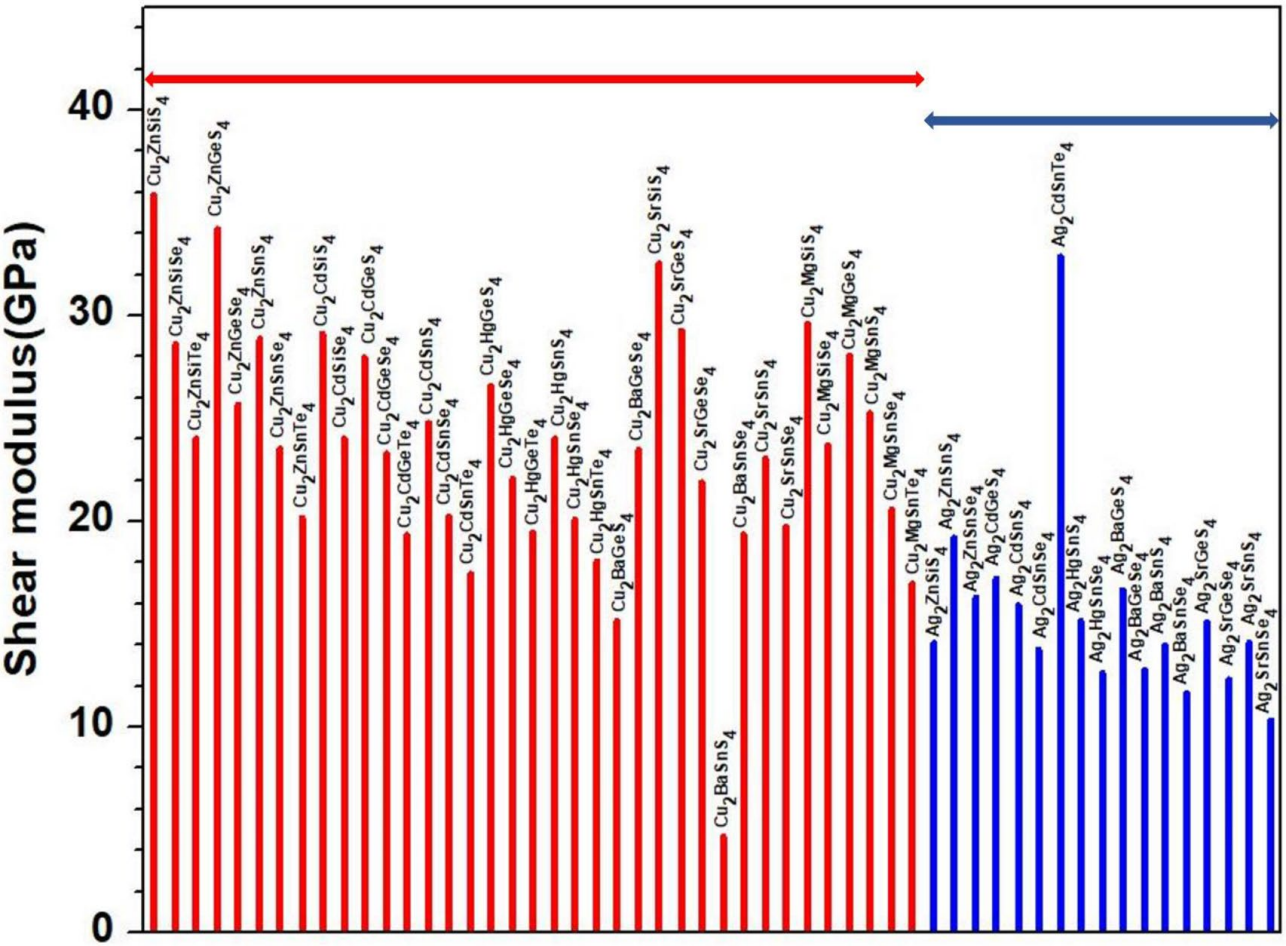
Distribution of shear modulus for the first group (54 crystals).

**Figure 12:**
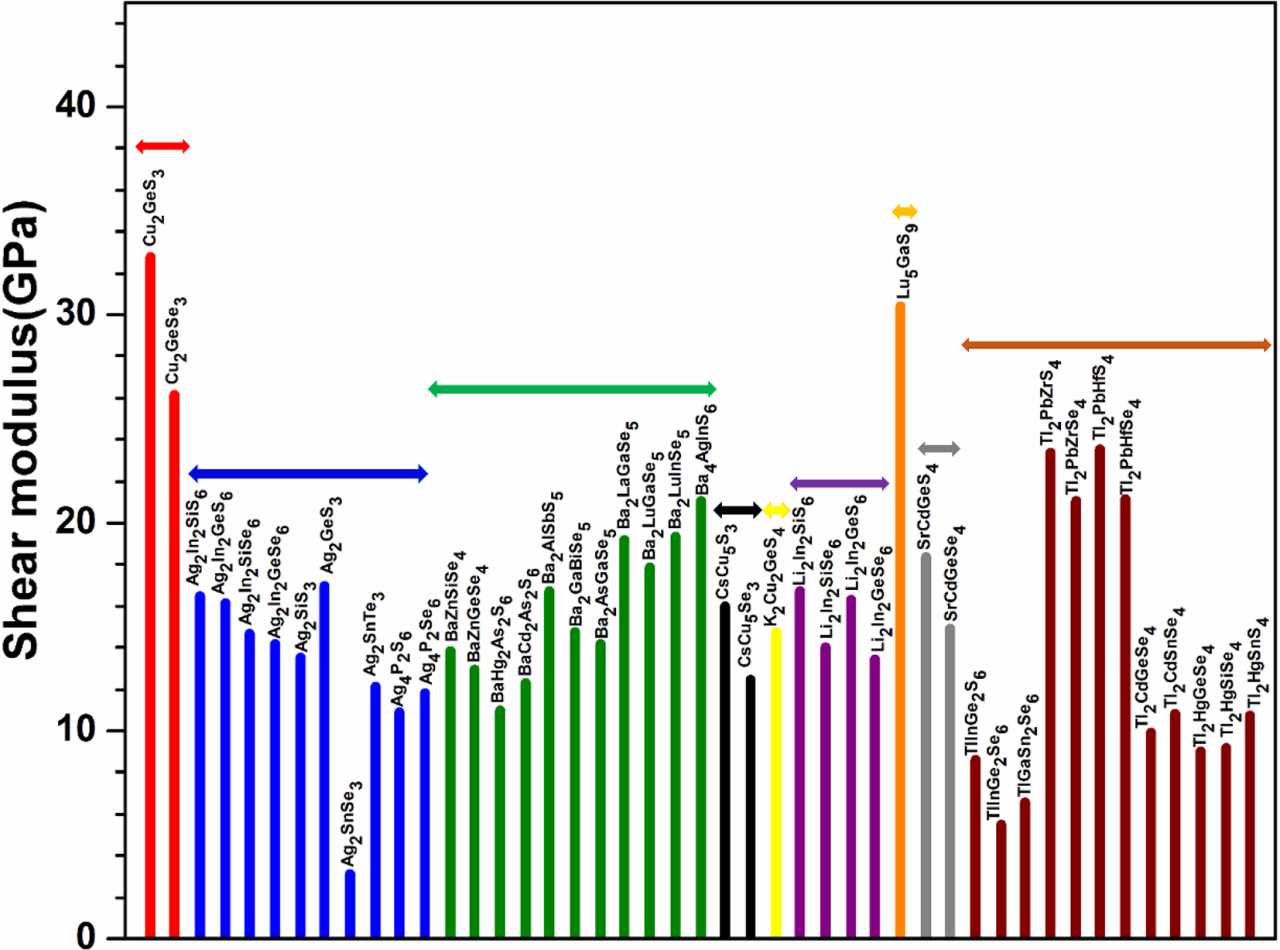
Distribution of shear modulus for the second group (45 crystals).

**Figure 13:**
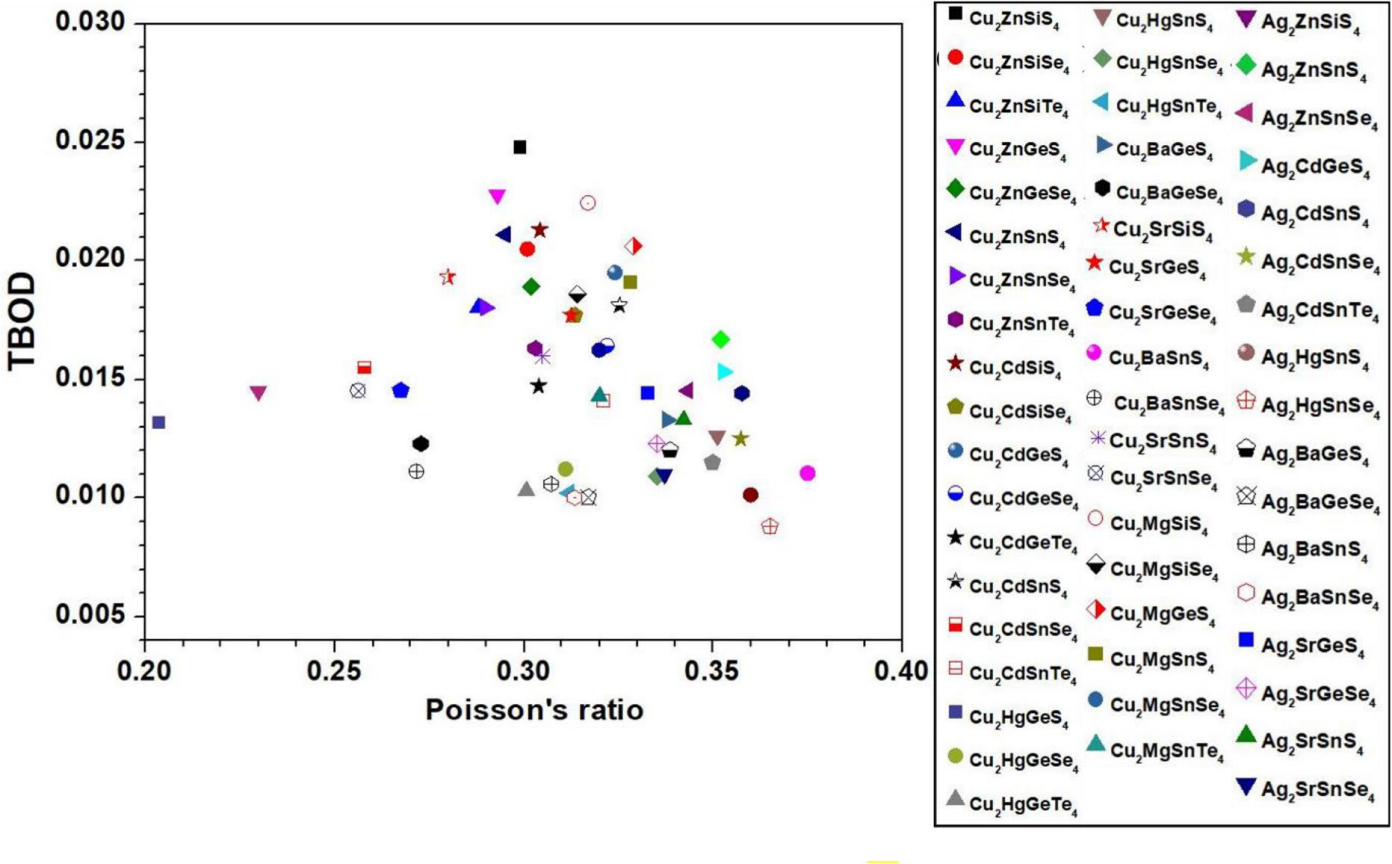
TBOD versus Poisson’s ratio for the first group (54 crystals) of crystals.

**Figure 14:**
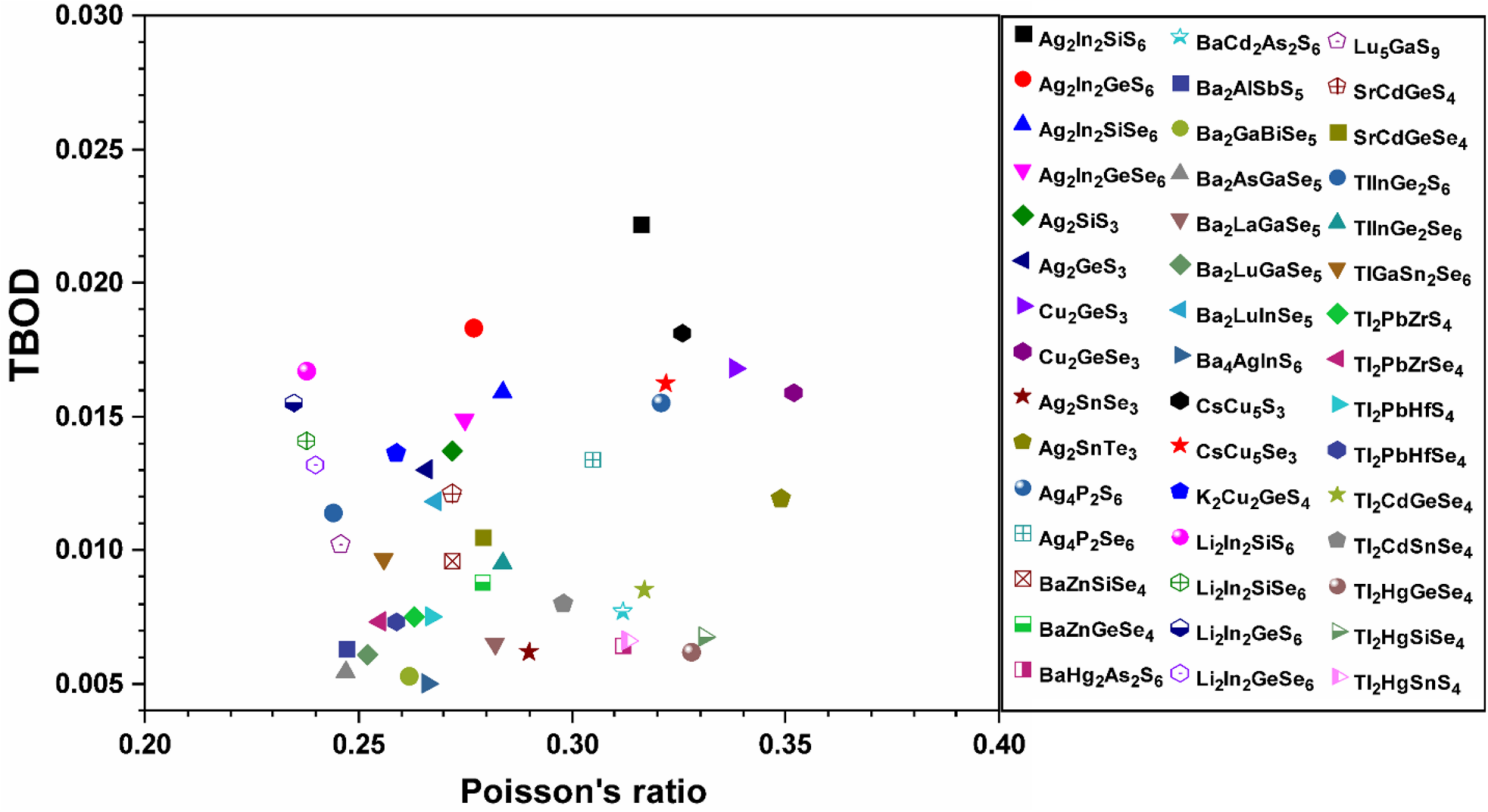
TBOD versus Poisson’s ratio for the second group (45 crystals) of crystals.

To explore the correlation in the mechanical properties of these 99 chalcogenides in more detail, the histogram plots of K in Figs. 9 and 10 divided into 2 main groups similar to Fig. 5 for the TBOD. Similar plots for the G in the 2 main groups are displayed in Figs. 11 and 12. The Cu-related crystals have higher bulk and shear modulus than the Ag-related crystals on average, while the data in the nine subgroups of the second main group crystals are again rather scattered. Still, we can note that Cu and Lu related crystals have high G and K, and some of the Tl related crystals have lowest values, with Ag, Ba, Li, K related crystals have intermediate and comparable values. Also, the first main group have higher K and G values than the second main group crystals. We also notice that K and G values decrease in moving from S to Se to Te. This feature can again be attributed to the larger size of Te atom resulting in weaker bond strength and more brittle crystals. The same feature has been observed for the TBOD and this implies a strong correlation between the mechanical properties and TBOD. Figures 13 and 14 show the scattered plots of TBOD versus Poisson’s ratio $\eta$ to explore any trend for these 99 crystals in two groups. It appears that the relationship between TBOD and Poisson’s ratio is not that much easy to explain and the data are scattered. In a broader scale, we can still observe a general trend that a larger TBOD implies a larger Poisson’s ratio.

**Figure 15:**
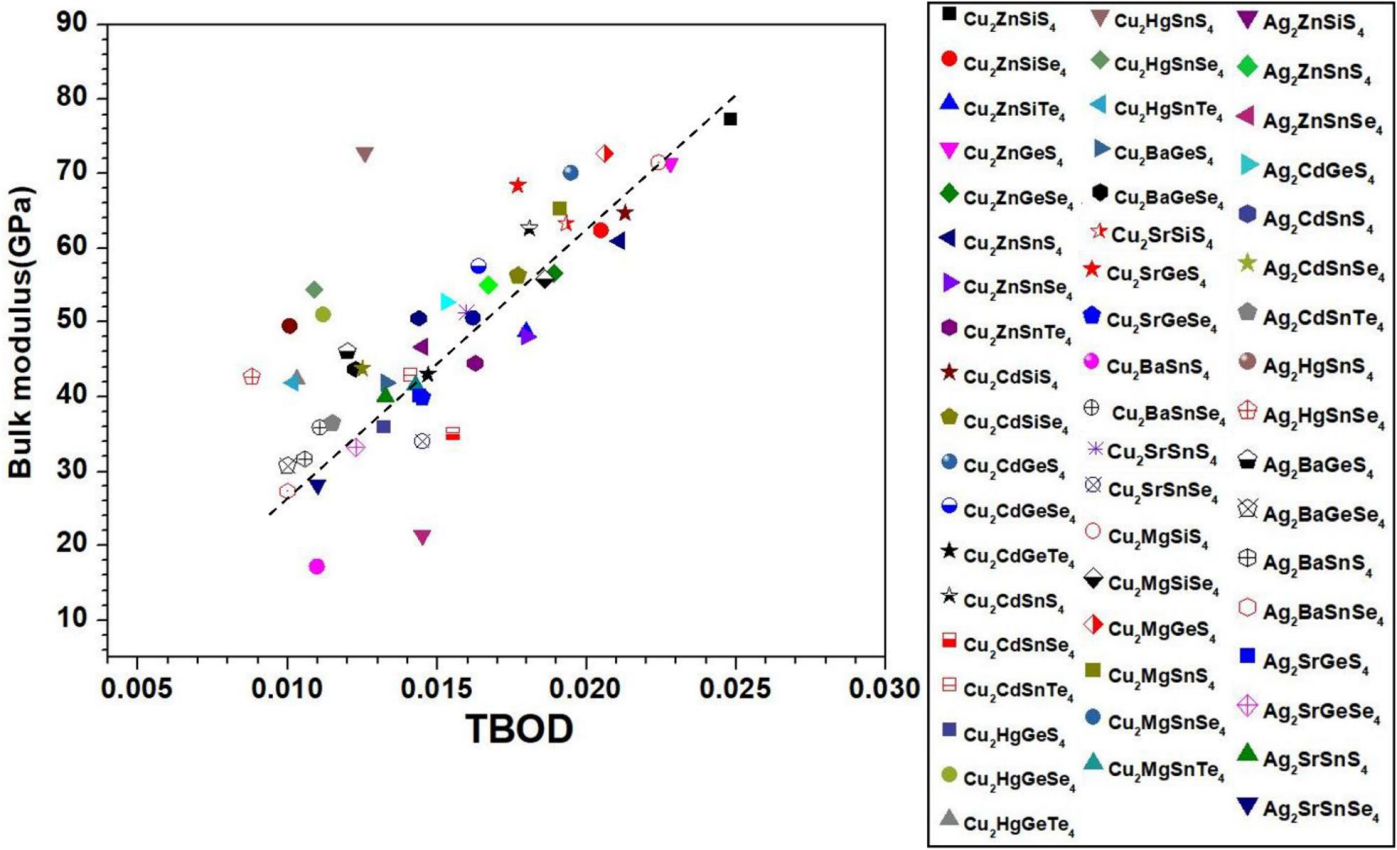
Bulk modulus versus TBOD for the first group (the 54 chalcogenide crystals).

**Figure 16:**
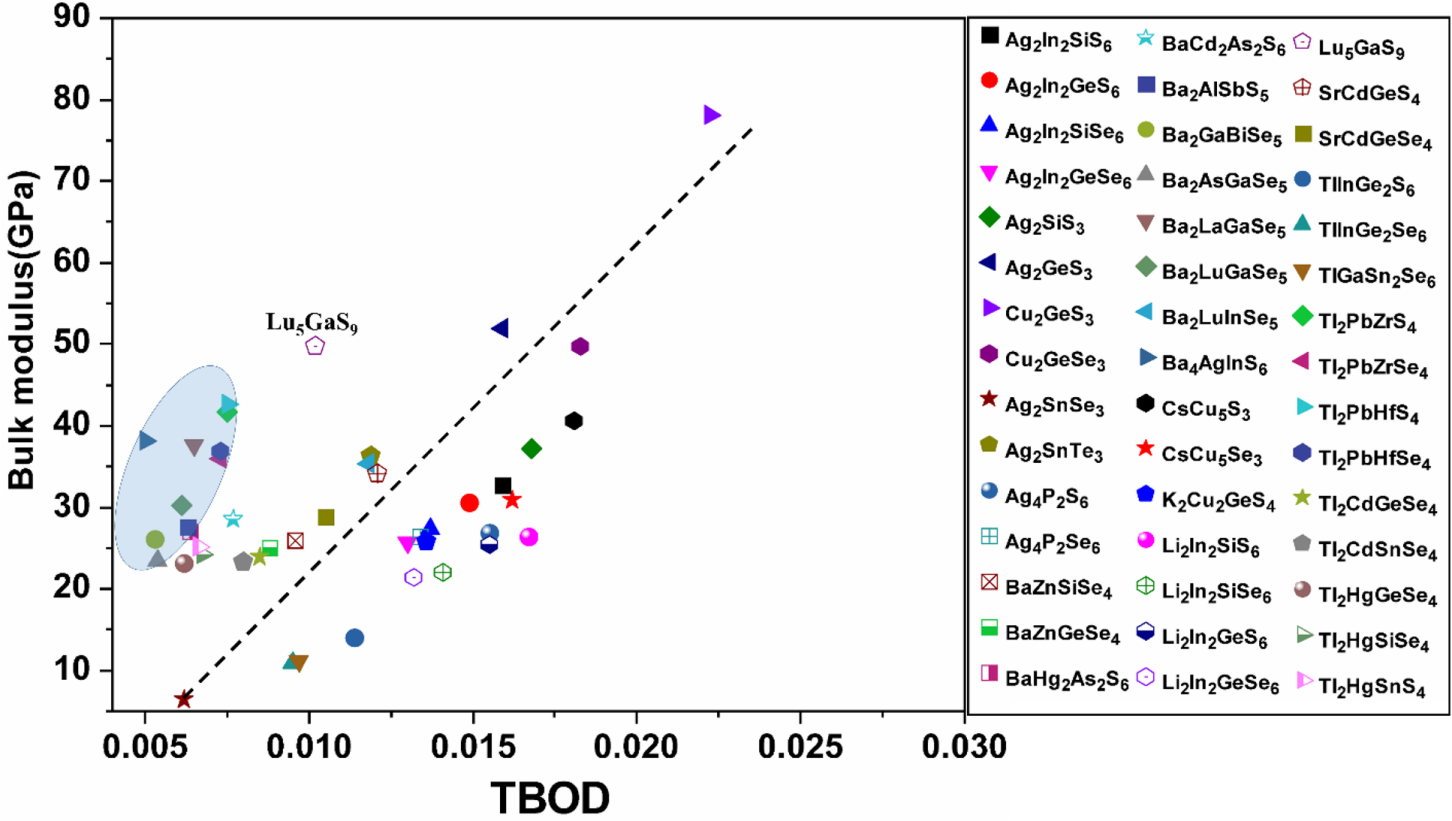
Bulk modulus versus TBOD for the second group (the 45 chalcogenide crystals).

**Figure 17:**
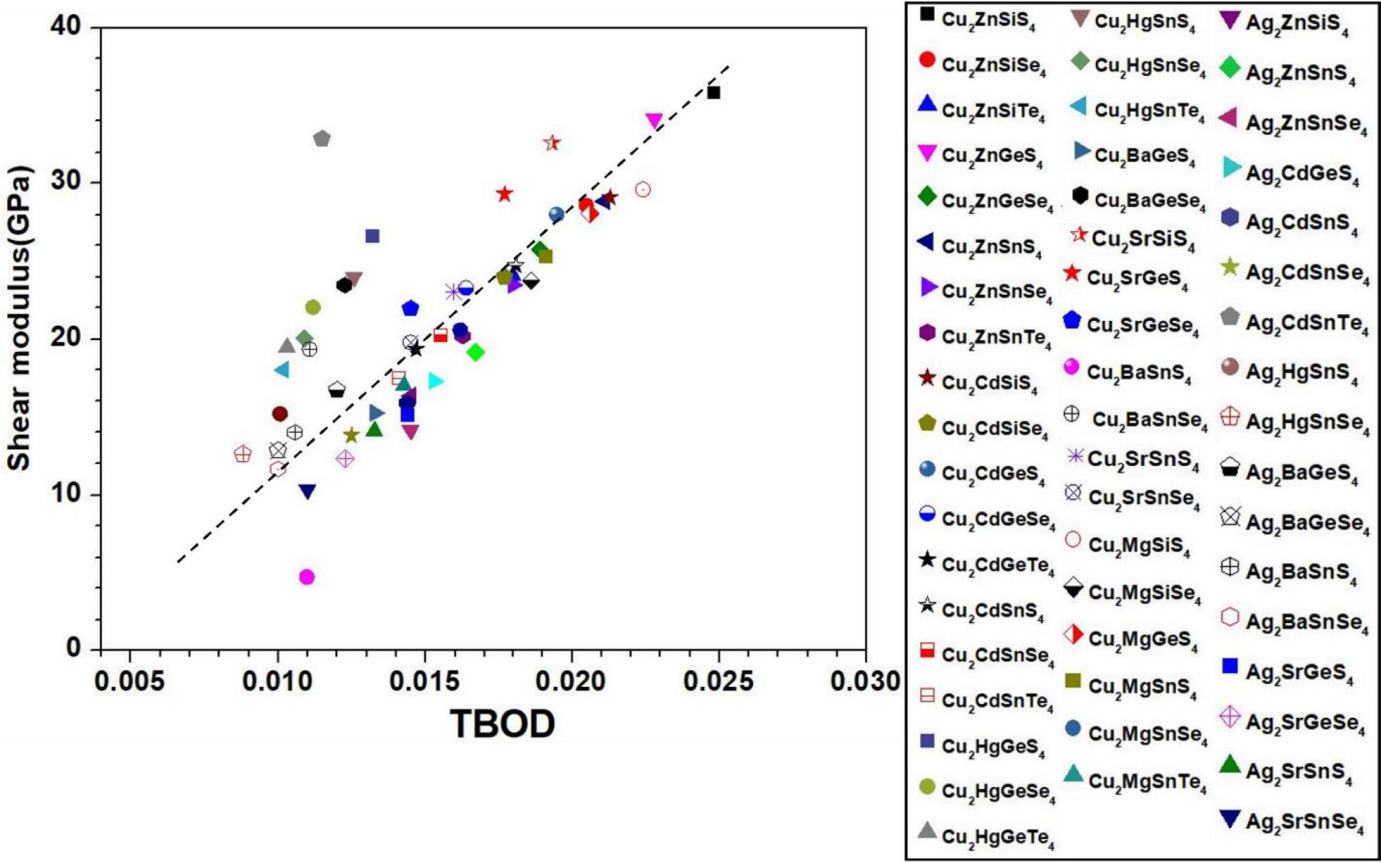
Shear modulus versus TBOD for the first group (the 54 chalcogenide crystals).

**Figure 18:**
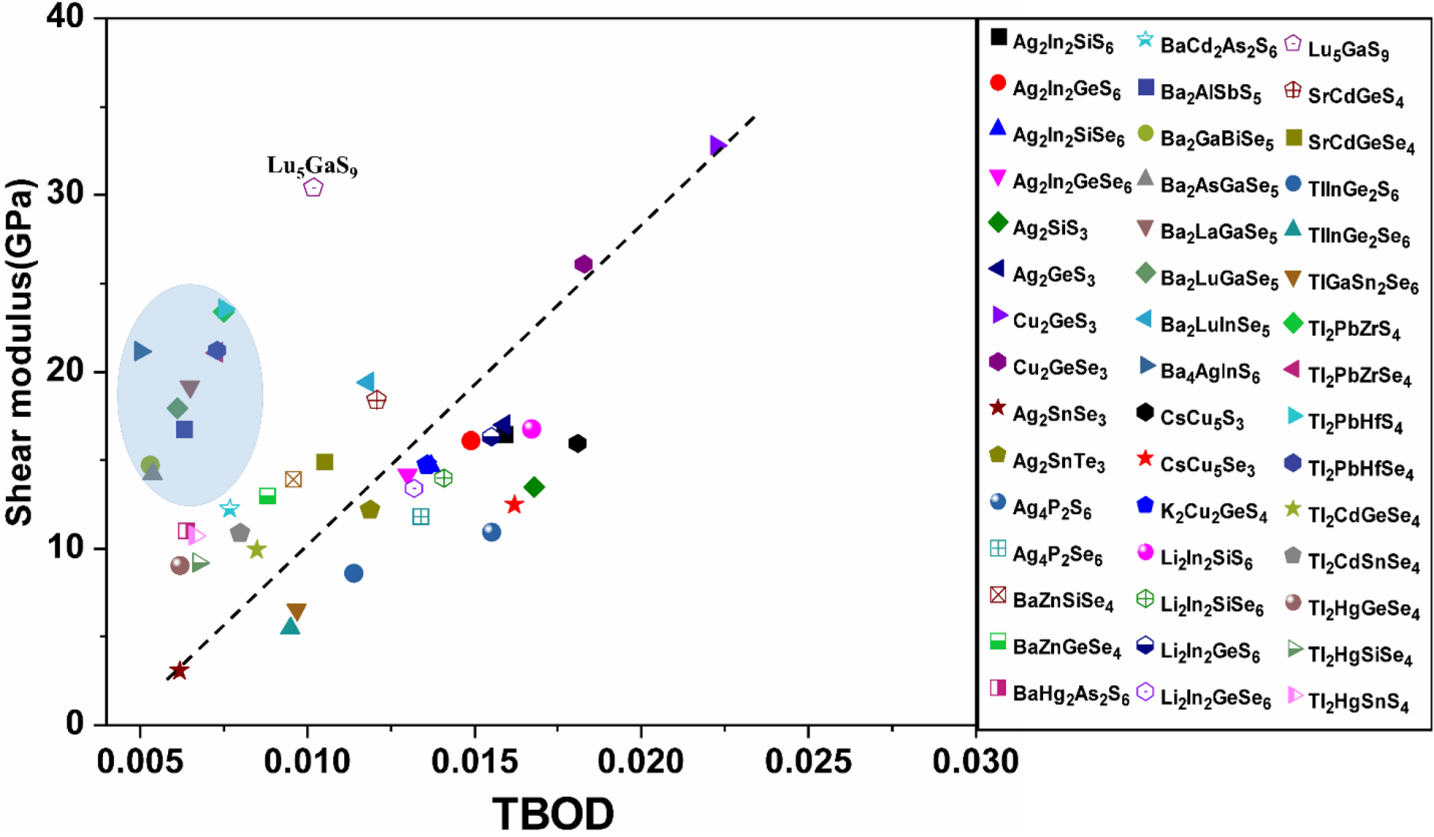
Shear modulus versus TBOD for the second group (the 45 chalcogenide crystals).

Identification of the underlying correlation between the electronic structure and the bonding characteristics of the chalcogenide crystals is one of the main objectives in this work. In this regard, exploring the connection between mechanical properties of these 99 crystals and the TBOD could be revealing. In Figs. 15, 16, 17, and 18, we plot the bulk modulus and shear modulus versus TBOD for the 99 crystals in the two separate groups. For the first main group of 54 crystals, there is a very clear correlation between the two moduli (K, G) and the TBOD. K and G increase linearly with the TBOD with only a few outliers. For the second main group of 45 crystals, the situation is slightly different. Moreover, we divided the crystals in this main group into two subgroups. In the first subgroup (71-Ba_2_AlSbS_5_, 72-Ba_2_GaBiSe_5_, 73-Ba_2_AsGaSe_5_, 74-Ba_2_LaGaSe_5_, 75-Ba_2_LuGaSe_5_, 77-Ba_4_AgInS_6_, 91-Tl_2_PbZrS_4_, 92-Tl_2_PbZrSe_4_, 93-Tl_2_PbHfS_4_, 94-Tl_2_PbHfSe_4_- mostly contains the Ba-related and some of the Tl-related crystals inside the blue circle), they are quite scattered with no clear correlation for K and G with TBOD. While in the second subgroup (the remaining crystals), there is a reasonable correlation with K and G steadily increasing linearly with TBOD. The crystal 85-Lu_5_GaS_9_ deviates from this behavior. It exhibits a sharp increase of G and K with TBOD. We conclude that the mechanical properties of these 99 chalcogenides are strongly related to the strength of the interatomic bonds which are collectively represented by the single matric TBOD.

## Conclusion

In conclusion, a comprehensive library of the electronic structure, interatomic bonding, optical, and mechanical properties of the 99 chalcogenides was assembled on the basis of extensive first-principles calculations. We summarize below the new insights obtained and the conclusions reached in this study by using the same approach and resulting in a pronounced consistency of the result.

1. Most of these chalcogenide crystals are semiconductors with small band gaps with few of them are semimetals. The reported results for 10-Cu_2_CdSiSe_4_, 16-Cu_2_CdSnTe_4_, 37-Cu_2_MgSnTe_4_, 64-Ag_2_SnTe_3_, 66-Ag_4_P_2_Se_6_, 69-BaHg_2_As_2_S_6_, 74-Ba_2_LaGaSe_5_, 75-Ba_2_LuGaSe_5_, 76-Ba_2_LuInSe_5_, 95-Tl_2_CdGeSe_4_, 96-Tl_2_CdSnSe_4_, 97-Tl_2_HgGeSe_4_, and 99-Tl_2_HgSnS_4_ crystals are new theoretical prediction.
2. Several of these crystals have very small energy gaps of about 0.005–0.235 eV including 18-Cu_2_HgGeSe_4_, 5-Cu_2_ZnGeSe_4_, 12-Cu_2_CdGeSe_4_, 13-Cu_2_CdGeTe_4_, 15-Cu_2_CdSnSe_4_, 19-Cu_2_HgGeTe_4_, 20-Cu_2_HgSnS_4_, 61-Cu_2_GeS_3_, 79-CsCu_5_Se_3_.
3. Analysis of the atomic partial charges shows Cu and Ag atoms are electronegative in some crystals, especially in the ones that contains Se and Te. This new finding is consistent with our recent work on other 32 simpler chalcogenide crystals.
4. In the first main group of crystals, the crystals A_2_BCQ_4_ with A = Cu; B = Zn, Mg; C = Si, Ge; Q = S) have the highest TBOD, while the crystals A_2_BCQ_4_ (with A = Cu, Ag; B = Hg; C = Sn; Q = Se, Te) have the lowest TBOD. In the second main group, the Cu, Cs-related crystals have the highest TBOD and the Ba-related crystals have the lowest TBOD. The Ag-related, Cs-related and Li-related crystals have very similar TBOD.
5. The optical spectra of the 99 crystals are calculated within the random phase approximation. They have medium values for the refractive index n in the range of 4.424–4.524.
6. The mechanical properties for these 99 crystals show large variations between the two main groups (A_2_BCQ_4_) and (A_x_B_y_C_z_Q_n_). The first group crystals tend to be more ductile than those from the second group. They are both reasonably correlated with the TBOD. The results and correlations summarized above can facilitate the design of new chalcogenide crystals and glasses for potential novel applications. They constitute a formidable database for complex chalcogenide crystals that was not available before. We believe that the present work can facilitate the discovery and production of good-quality chalcogenides with a variety of energy or environmental applications.

## Supplementary Information

Supplementary Information

## Data Availability

All the data in this paper including those in the supplementary materials are freely available by contacting the one of the corresponding authors (chingw@umkc.edu)
