# Supplementary material for: Structural and physical properties of 99 complex bulk chalcogenides crystals using first-principles calculations
Source: Sci Rep. 2021 May 10;11:9921. doi: 10.1038/s41598-021-89281-6 (PMC8110807; doi:10.1038/s41598-021-89281-6)
Supplement: Supplementary file 1 — Supplementary Information [file 41598_2021_89281_MOESM1_ESM.pdf]

# A comprehensive library of bulk chalcogenides: structural, electronic, optical and elastic properties using first-principles calculations

Sahib Hasan[1], Khagendra Baral[1], Neng Li[2,] and Wai-Yim Ching[1]*

1. Department of Physics and Astronomy, University of Missouri-Kansas City, Kansas City, Missouri, 64110 USA
2. School of Materials Science and Engineering, Wuhan University of Technology, No. 122, Luoshi Road, Wuhan, 430070, China

* Corresponding Author e-mail: Chingw@umkc.edu; lineng@whut.edu.cn

## 1. Optical properties

We can use OLCAO method to calculate the optical properties of crystalline chalcogenides based on the theory of inter-band optical absorptions within the random phase approximation. According to this approach, the optical conductivity is given by:

$$\sigma_1(\hbar w) = \frac{2\Pi e \hbar^2}{3m^2\omega\Omega}\sum_{n,l} |\langle n|\vec{p}|l\rangle^2 f_l[1-f_n]\delta(E_n - E_l - \hbar\omega) \quad (1)$$

$f_l$ is the Fermi-Dirac function of the occupied band state ι and δ function makes sure that the energy is conserved in the transition process between occupied state and unoccupied state with energy Eι and En respectively. Ω is the volume of the unit cell.

There is no intra-band transitions for the materials with an energy gap in their band structure, so that the optical properties can be described by the complex dielectric function which is related to the $\sigma_1(\hbar w)$ by:

$$\varepsilon_2(w) = 4\pi\frac{\sigma_1(w)}{w} \quad (2)$$

By transforming the summation over the unit cell in the equation (3) to an integral over the BZ in **k** space, $\varepsilon_2(w)$ can be given by:

$$\varepsilon_2(\hbar\omega) = \frac{e^2}{\pi m\omega^2}\int dk^3 \sum_{n,l}\left|\langle\Psi_n(\vec{k},\vec{r})\middle|-i\hbar\vec{\nabla}\middle|\Psi_l(\vec{k},\vec{r})\rangle\right|^2 f_l(\vec{k})[1 - f_n(\vec{k})]\delta(E_n(\vec{k}) - E_l(\vec{k}) - \hbar\omega) \quad (3)$$

The real part of the dielectric function can be derived from $\varepsilon_2$ by using Kramers-Kronig relation:

$$\varepsilon_1(\hbar\omega) = 1 + \frac{2}{\pi}P\int_0^\infty \frac{s\varepsilon_2(\hbar\omega)}{s^2-\omega^2}\,ds \quad (4)$$

In OLCAO method, the momentum matrices in the Eqs (2) and (4) for the optical transitions can be easily calculated as sums of two –center integrals between GTOs[1].

## 2. Mechanical properties

Ab initio calculation method within DFT for the mechanical properties is very powerful method these days. In this section, and to calculate the mechanical properties for the seven crystals, we followed the method of the Hongzhi Yao. et al. work[2]. In this work, a scheme has been applied to calculate the elastic constants of crystals. Starting with the simple Hook's famous law that can relate the stress components $\sigma_i$ with the strain components $\varepsilon_j$ by relation:

$$\sigma_i = \sum_{j=1}^{6} C_{ij}\, \varepsilon_j \quad (5)$$

$C_{ij}$ is the elastic constants of the crystals. From knowing the elastic constants, we could calculate other mechanical properties like compliance tensor $S_{ij}$, Young's modulus (E), Bulk modulus ( K), Shear modulus ( G), and Poisson's ratio (η ). Here, Voigt – Reuss –Hill ( VRH)[3, 4] approximation has been used to derive the above mechanical properties. According to this approximation, the upper and lower bounds for the structural parameters, like bulk and shear modulus are respectively given by:

$$K_V = \frac{(C_{11}+C_{22}+C_{33})}{9} + \frac{2(C_{12}+C_{13}+C_{23})}{9} \quad (6)$$

$$K_R = \frac{1}{(S_{11}+S_{22}+S_{33})+2(S_{12}+S_{13}+S_{23})} \quad (7)$$

$$G_V = \frac{(C_{11}+C_{22}+C_{33}-C_{12}-C_{13}-C_{23})}{15} + \frac{(C_{44}+C_{55}+C_{66})}{5} \quad (8)$$

$$G_R = \frac{15}{4(S_{11}+S_{22}+S_{33})-4(S_{12}+S_{13}+S_{23})+3(S_{44}+S_{55}+S_{66})} \quad (9)$$

So, the average values of the mechanical properties are given by:

$$K = \frac{(K_V+K_R)}{2} \quad (10)$$

$$G = \frac{(G_V+G_R)}{2} \quad (11)$$

$$E = \frac{9KG}{(3K+G)} \quad (12)$$

$$\eta = \frac{(3K-2G)}{2(3K+G)} \quad (13)$$

## 3. The Supplementary Tables

**Table S1** The symmetry, lattice parameters after full VASP relaxation, and the experimental lattice parameters data of the 99 crystals used in our calculations, where GGA has been used as an exchange and correlation potential. (M for monoclinic, T for triclinic, Tr for Trigonal, Tet for tetragonal, O for orthorhombic. The full relaxed crystal structure data are available upon request with a corresponding author. They can also be found on the web site of our research group: **https://info.umkc.edu/ching-esg/?page_id=152**

| # | Crystal | Space group | Lattice parameters(our calculations) a, b, c(Å) in 4 decimal points, α, β, γ(deg.) in 2 decimal points | Lattice parameters(experimental) a, b, c(Å) in 4 decimal points, α, β, γ(deg.) in 2 decimal points |
|---|---|---|---|---|
| 1 | $Cu_2ZnSiS_4$ | O, Pmn$2_1$(31) | 7.3849, 6.4683, 6.1669, 90.00, 90.00, 90.00 | 7.4374, 6.4001, 6.1394, 90.00, 90.00, 90.00[5] |
| 2 | $Cu_2ZnSiSe_4$ | O, Pmn$2_1$(31) | 7.8439, 6.8452, 6.5190, 90.00, 90.00, 90.00 | 7.8287, 6.7342, 6.4568, 90.00, 90.00, 90.00[6] |
| 3 | $Cu_2ZnSiTe_4$ | Tet, I -4 2 m(121) | 6.0501, 6.0501, 11.982, 90.00, 90.00, 90.00 | 5.9612, 5.9612, 11.7887, 90.00, 90.00, 90.00[7] |
| 4 | $Cu_2ZnGeS_4$ | O, Pmn$2_1$(31) | 7.5152, 6.5574, 6.2375, 90.00, 90.00, 90.00 | 7.5090, 6.4790, 6.1920, 90.00, 90.00, 90.00[8] |
| 5 | $Cu_2ZnGeSe_4$ | Tet, I -4(82) | 5.6726, 5.6726, 11.2758, 90.00, 90.00, 90.00 | 5.6112, 5.6112, 11.0473, 90.00, 90.00, 90.00[9] |
| 6 | $Cu_2ZnSnS_4$ | Tet, I -4(82) | 5.4704, 5.4704, 10.9424, 90.00, 90.00, 90.00 | 5.4280, 5.4280, 10.8640, 90.00, 90.00, 90.00[10] |
| 7 | $Cu_2ZnSnSe_4$ | Tet, I -4(82) | 5.7674, 5.7674, 11.5315, 90.00, 90.00, 90.00 | 5.6920, 5.6920, 11.3400, 90.00, 90.00, 90.00[11] |
| 8 | $Cu_2ZnSnTe_4$ | Tet, I -4(82) | 6.1880, 6.1880, 12.3655, 90.00, 90.00, 90.00 | 6.1300, 6.1300, 12.2071, 90.00, 90.00, 90.00[12] |
| 9 | $Cu_2CdSiS_4$ | O, Pmn$2_1$(31) | 7.6707, 6.5086, 6.2901, 90.00, 90.00, 90.00 | 7.5980, 6.4860, 6.2580, 90.00, 90.00, 90.00[13] |
| 10 | $Cu_2CdSiSe_4$ | O, Pmn$2_1$(31) | 8.1096, 6.8922, 6.6344, 90.00, 90.00, 90.00 | 7.9900, 6.8240, 6.2640, 90.00, 90.00, 90.00[14] |
| 11 | $Cu_2CdGeS_4$ | O, Pmn$2_1$(31) | 7.7804, 6.6253, 6.3608, 90.00, 90.00, 90.00 | 7.6920, 6.5550, 6.2990, 90.00, 90.00, 90.00[15] |
| 12 | $Cu_2CdGeSe_4$ | Tet, I -4 2 m(121) | 5.8449, 5.8449, 11.2495, 90.00, 90.00, 90.00 | 5.7482, 5.7482, 11.0533, 90.00, 90.00, 90.00[16] |
| 13 | $Cu_2CdGeTe_4$ | Tet, I -4 2 m(121) | 6.2531, 6.2531, 12.1218, 90.00, 90.00, 90.00 | 6.1270, 6.1270, 11.9190, 90.00, 90.00, 90.00[17] |
| 14 | $Cu_2CdSnS_4$ | Tet, I -4(82) | 5.5763, 5.5763, 11.2449, 90.00, 90.00, 90.00 | 5.4870, 5.4870, 10.8450, 90.00, 90.00, 90.00[18] |
| 15 | $Cu_2CdSnSe_4$ | Tet, I -4(82) | 5.8715, 5.8715, 11.8107, 90.00, 90.00, 90.00 | 5.8320, 5.8320, 11.3900, 90.00, 90.00, 90.00[18] |
| 16 | $Cu_2CdSnTe_4$ | Tet, I -4(82) | 6.2917, 6.2917, 12.5987, 90.00, 90.00, 90.00 | 6.1980, 6.1980, 12.2560, 90.00, 90.00, 90.00[18] |
| 17 | $Cu_2HgGeS_4$ | Tet, I -4 2 m(121) | 5.5658, 5.5658, 10.6581, 90.00, 90.00, 90.00 | 5.4930, 5.4930, 10.5510, 90.00, 90.00, 90.00[19] |
| 18 | $Cu_2HgGeSe_4$ | Tet, I -4 2 m(121) | 5.8695, 5.8695, 11.2322, 90.00, 90.00, 90.00 | 5.7445, 5.7445, 11.0824, 90.00, 90.00, 90.00[20] |
| 19 | $Cu_2HgGeTe_4$ | Tet, I -4 2 m(121) | 6.2629, 6.2629, 12.1144, 90.00, 90.00, 90.00 | 6.12940, 6.12940, 11.9374, 90.00, 90.00, 90.00[21] |
| 20 | $Cu_2HgSnS_4$ | Tet, I -4(82) | 5.6439, 5.6439, 11.0670, 90.00, 90.00, 90.00 | 5.5749, 5.5749, 10.8820, 90.00, 90.00, 90.00[22] |
| 21 | $Cu_2HgSnSe_4$ | Tet, I -4 2 m(121) | 5.9393, 5.9393, 11.6174, 90.00, 90.00, 90.00 | 5.8288, 5.8288, 11.4179, 90.00, 90.00, 90.00[23] |
| 22 | $Cu_2HgSnTe_4$ | Tet, I -4 2 m(121) | 6.3310, 6.3310, 12.4851, 90.00, 90.00, 90.00 | 6.1957, 6.1957, 12.2874, 90.00, 90.00, 90.00[24] |
| 23 | $Cu_2BaGeS_4$ | Tr, P $3_1$(144) | 6.5328, 6.5328, 15.2783, 90.00, 90.00, 119.99 | 6.2150, 6.2150, 15.5340, 90.00, 90.00, 120.00[25] |
| 24 | $Cu_2BaGeSe_4$ | Tr, P $3_1$(144) | 6.6141, 6.6141, 16.4332, 90.00, 90.00, 119.99 | 6.4900, 6.4900, 16.3550, 90.00, 90.00, 120.00[26] |
| 25 | $Cu_2SrSiS_4$ | Tr, P $3_1$(144) | 6.1049, 6.1049, 15.1617, 90.00, 90.00, 120.00 | 6.0687, 6.0687, 15.1373, 90.00, 90.00, 120.00[27] |
| 26 | $Cu_2SrGeS_4$ | Tr, P $3_2$(145) | 6.2025, 6.2025, 15.3469, 90.00, 90.00, 120.00 | 6.1430, 6.1430, 15.2820, 90.00, 90.00, 120.00[25] |
| 27 | $Cu_2SrGeSe_4$ | O, Ama2(40) | 10.9140, 10.8817, 6.6361, 90.00, 90.00, 90.00 | 10.8070, 10.7350, 6.5410, 90.00, 90.00, 90.00[26] |
| 28 | $Cu_2BaSnS_4$ | Tr, P $3_1$(144) | 6.8386, 6.8386, 16.0392, 90.00, 90.00, 120.00 | 6.3660, 6.3660, 15.8280, 90.00, 90.00, 120.00[25] |
| 29 | $Cu_2BaSnSe_4$ | O, Ama2(40) | 11.2245, 11.4509, 6.8533, 90.00, 90.00, 90.00 | 11.1110, 11.2270, 6.7440, 90.00, 90.00, 90.00[25] |
| 30 | $Cu_2SrSnS_4$ | Tr, P $3_1$(144) | 6.3616, 6.3616, 15.6245, 90.00, 90.00, 119.99 | 6.367, 6.367, 15.833, 90.00, 90.00, 120.00[28] |
| 31 | $Cu_2SrSnSe_4$ | O, Ama2(40) | 11.1162, 10.9248, 6.7886, 90.00, 90.00, 90.00 | 10.9670, 10.7540, 6.6950, 90.00, 90.00, 90.00[25] |
| 32 | $Cu_2MgSiS_4$ | O, Pmn$2_1$(31) | 7.5952, 6.4612, 6.2147, 90.00, 90.00, 90.00 | 7.5630, 6.4480, 6.1790, 90.00, 90.00, 90.00[29] |
| 33 | $Cu_2MgSiSe_4$ | O, Pmn$2_1$(31) | 8.0300, 6.8564, 6.5743, 90.00, 90.00, 90.00 | 7.9530, 6.7970, 6.5070, 90.00, 90.00, 90.00[30] |
| 34 | $Cu_2MgGeS_4$ | O, Pmn$2_1$(31) | 7.6930, 6.5724, 6.2865, 90.00, 90.00, 90.00 | 7.6380, 6.5150, 6.2250, 90.00, 90.00, 90.00[29] |
| 35 | $Cu_2MgSnS_4$ | Tet, I -4(82) | 5.5091, 5.5091, 11.1542, 90.00, 90.00, 90.00 | 5.4360, 5.4360, 11.0180, 90.00, 90.00, 90.00[31] |
| 36 | $Cu_2MgSnSe_4$ | Tet, I -4(82) | 5.8184, 5.8184, 11.7249, 90.00, 90.00, 90.00 | 5.7260, 5.7260, 11.5600, 90.00, 90.00, 90.00[32] |
| 37 | $Cu_2MgSnTe_4$ | Tet, I -4(82) | 6.2692, 6.2692, 12.5476, 90.00, 90.00, 90.00 | 6.1730, 6.1730, 12.3398, 90.00, 90.00, 90.00[32] |
| 38 | $Ag_2ZnSiS_4$ | M, Pn(7) | 6.6957, 7.7129, 8.1202, 90.00, 90.52, 90.00 | 6.4052, 6.5484, 7.9340, 90.00, 90.45, 90.00[33] |
| 39 | $Ag_2ZnSnS_4$ | Tet, I -4(82) | 5.8408, 5.8408, 11.0654, 90.00, 90.00, 90.00 | 5.8126, 5.8126, 10.7788, 90.00, 90.00, 90.00[34] |
| 40 | $Ag_2ZnSnSe_4$ | Tet, I -4(82) | 6.1106, 6.1106, 11.6683, 90.00, 90.00, 90.00 | 6.0442, 6.0442, 11.3065, 90.00, 90.00, 90.00[34] |
| 41 | $Ag_2CdGeS_4$ | O, Pna$2_1$(33) | 13.9844, 8.1157, 6.6928, 90.00, 90.00, 90.00 | 13.7415, 8.0367, 6.5907, 90.00, 90.00, 90.00[35] |
| 42 | $Ag_2CdSnS_4$ | Tet, I -4(82) | 5.9150, 5.9150, 11.5511, 90.00, 90.00, 90.00 | 5.9180, 5.9180, 11.5520, 90.00, 90.00, 90.00[36] |
| 43 | $Ag_2CdSnSe_4$ | Tet, I -4(82) | 6.1844, 6.1844, 12.1072, 90.00, 90.00, 90.00 | 6.1780, 6.1780, 12.1080, 90.00, 90.00, 90.00[37] |
| 44 | $Ag_2CdSnTe_4$ | Tet, I -4(82) | 6.5545, 6.5545, 12.9953, 90.00, 90.00, 90.00 | 6.5630, 6.5630, 12.9940, 90.00, 90.00, 90.00[37] |
| 45 | $Ag_2HgSnS_4$ | O, Pmn$2_1$(31) | 8.1896, 7.2620, 6.8447, 90.00, 90.00, 90.00 | 8.2074, 7.0330, 6.7180, 90.00, 90.00, 90.00[38] |
| 46 | $Ag_2HgSnSe_4$ | O, Pmn$2_1$(31) | 8.6148, 7.5571, 7.1617, 90.00, 90.00, 90.00 | 8.4610, 7.3400, 6.9901, 90.00, 90.00, 90.00[39] |

| | | | | |
|---|---|---|---|---|
| 47 | $Ag_2BaGeS_4$ | Tet, I -4 2 m(121) | 6.8499, 6.8499, 8.3308, 90.00, 90.00, 90.00 | 6.8280, 6.8280, 8.0170, 90.00, 90.00, 90.00[25] |
| 48 | $Ag_2BaGeSe_4$ | O, I 222(23) | 7.0516, 7.5208, 8.4835, 90.00, 90.00, 90.00 | 7.0580, 7.2630, 8.2630, 90.00, 90.00, 90.00[25] |
| 49 | $Ag_2BaSnS_4$ | O, I 222(23) | 7.3760, 8.3261, 6.8488, 90.00, 90.00, 90.00 | 7.1270, 8.1170, 6.8540, 90.00, 90.00, 90.00[25] |
| 50 | $Ag_2BaSnSe_4$ | O, I 222(23) | 7.1503, 7.7114, 8.5929, 90.00, 90.00, 90.00 | 7.1160, 7.4990, 8.3370, 90.00, 90.00, 90.00[25] |
| 51 | $Ag_2SrGeS_4$ | O, I 222(23) | 7.0432, 7.7739, 6.8062, 90.00, 90.00, 90.00 | 6.8200, 6.9730, 7.6900, 90.00, 90.00, 90.00[25] |
| 52 | $Ag_2SrGeSe_4$ | O, I 222(23) | 7.1171, 7.4129, 8.0634, 90.00, 90.00, 90.00 | 7.1150, 7.3890, 7.9510, 90.00, 90.00, 90.00[25] |
| 53 | $Ag_2SrSnS_4$ | O, I 222(23) | 7.2909, 7.8802, 6.8971, 90.00, 90.00, 90.00 | 6.9100, 7.2100, 7.8310, 90.00, 90.00, 90.00[25] |
| 54 | $Ag_2SrSnSe_4$ | O, I 222(23) | 7.1924, 7.6542, 8.1501, 90.00, 90.00, 90.00 | 7.1930, 7.6570, 8.0340, 90.00, 90.00, 90.00[25] |
| 55 | $Ag_2In_2SiS_6$ | M, C1c1(9) | 12.4013, 7.2787, 12.3814, 90.00, 109.43, 90.00 | 12.1481, 7.1726, 12.1263, 90.00, 109.25, 90.00[40] |
| 56 | $Ag_2In_2GeS_6$ | M, C1c1(9) | 12.4988, 7.3491, 12.4713, 90.00, 109.50, 90.00 | 12.2316, 7.2190, 12.2124, 90.00, 109.50, 90.00[40] |
| 57 | $Ag_2In_2SiSe_6$ | M, C1c1(9) | 12.9619, 7.6205, 12.9006, 90.00, 109.39, 90.00 | 12.6683, 7.4565, 12.6133, 90.00, 109.29, 90.00[41] |
| 58 | $Ag_2In_2GeSe_6$ | M, C1c1(9) | 13.0354, 7.6909, 12.9695, 90.00, 109.47, 90.00 | 12.6920, 7.4920, 12.6440, 90.00, 109.50, 90.00[41] |
| 59 | $Ag_2SiS_3$ | M, P12$_1$ /c1(14) | 6.6871, 6.6825, 13.4925, 90.00, 118.21, 90.00 | 6.6709, 6.6567, 13.1748, 90.00, 118.66, 90.00[42] |
| 60 | $Ag_2GeS_3$ | O, Cmc21(36) | 11.9886, 7.0452, 6.5496, 90.00, 90.00, 90.00 | 11.7895, 7.0751, 6.3420, 90.00, 90.00, 90.00[43] |
| 61 | $Cu_2GeS_3$ | M, C1c1(9) | 6.5056, 11.3409, 6.5456, 90.00, 109.20, 90.00 | 6.4490, 11.3190, 6.4280, 90.00, 108.37, 90.00[44] |
| 62 | $Cu_2GeSe_3$ | O, I mm2(44) | 11.8886, 4.0560, 5.5967, 90.00, 90.00, 90.00 | 11.8540, 3.9540, 5.4890, 90.00, 90.00, 90.00[45] |
| 63 | $Ag_2SnSe_3$ | M, P12 /m1(10) | 8.3969, 9.2348, 12.9899, 90.00, 105.61, 90.00 | 7.9770, 7.9120, 13.0230, 90.00, 101.73, 90.00[46] |
| 64 | $Ag_2SnTe_3$ | M, C1c1(9) | 7.9259, 13.8274, 7.9704, 90.00, 109.03, 90.00 | 7.4420, 12.8377, 7.4025, 90.00, 109.54, 90.00[47] |
| 65 | $Ag_4P_2S_6$ | O, P2$_1$2$_1$2$_1$(19) | 14.3859, 11.2296, 6.3246, 90.00, 90.00, 90.00 | 13.9010, 11.0730, 6.3030, 90.00, 90.00, 90.00[48] |
| 66 | $Ag_4P_2Se_6$ | M, C1c1(9) | 14.6566, 11.7643, 6.6156, 90.00, 90.00, 90.00 | 14.1690, 11.5980, 6.5850, 90.00, 90.00, 90.00[49] |
| 67 | $BaZnSiSe_4$ | O, Ama2(40) | 11.5432, 11.4761, 6.2595, 90.00, 90.00, 90.00 | 11.3055, 11.2344, 6.1994, 90.00, 90.00, 90.00[50] |
| 68 | $BaZnGeSe_4$ | O, Ama2(40) | 11.5889, 11.5015, 6.3670, 90.00, 90.00, 90.00 | 11.3255, 11.2527, 6.2917, 90.00, 90.00, 90.00[50] |
| 69 | $BaHg_2As_2S_6$ | O, Cmca(64) | 8.3779, 8.4205, 16.5401, 90.00, 90.00, 90.00 | 8.1304, 7.9417, 16.3280, 90.00, 90.00, 90.00[51] |
| 70 | $BaCd_2As_2S_6$ | O, Cmca(64) | 8.1111, 8.1509, 17.1184, 90.00, 90.00, 90.00 | 7.9544, 7.8649, 16.9790, 90.00, 90.00, 90.00[51] |
| 71 | $Ba_2AlSbS_5$ | O, Pnma(62) | 12.3352, 8.9488, 9.1673, 90.00, 90.00, 90.00 | 12.1400, 8.8911, 8.9552, 90.00, 90.00, 90.00[52] |
| 72 | $Ba_2GaBiSe_5$ | O, Pnma(62) | 12.9159, 9.2489, 9.5312, 90.00, 90.00, 90.00 | 12.6910, 9.1900, 9.2450, 90.00, 90.00, 90.00[52] |
| 73 | $Ba_2AsGaSe_5$ | O, Pnma(62) | 12.9076, 9.1005, 9.4202, 90.00, 90.00, 90.00 | 12.6320, 8.9726, 9.2029, 90.00, 90.00, 90.00[53] |
| 74 | $Ba_2LaGaSe_5$ | O, Pnma(62) | 12.7079, 9.5926, 8.9275, 90.00, 90.00, 90.00 | 12.5049, 9.6288, 8.7355, 90.00, 90.00, 90.00[54] |
| 75 | $Ba_2LuGaSe_5$ | T, P-1(2) | 7.3981, 8.7027, 9.5441, 103.29, 102.87, 107.25 | 7.2829, 8.6120, 9.3681, 103.36, 103.05, 107.31[54] |
| 76 | $Ba_2LuInSe_5$ | O, Cmc21(36) | 4.2872, 19.1369, 13.4878, 90.00, 90.00, 90.00 | 4.2345, 18.7530, 13.2854, 90.00, 90.00, 90.00[54] |
| 77 | $Ba_4AgInS_6$ | M, P12$_1$ /c1(14) | 8.8287, 9.0183, 18.0285, 90.00, 104.12, 90.00 | 8.7241, 8.8799, 17.7930, 90.00, 104.14, 90.00[55] |
| 78 | $CsCu_5S_3$ | O, Pmma(51) | 9.6935, 3.9345, 9.1146, 90.00, 90.00, 90.00 | 9.6365, 3.9547, 8.9490, 90.00, 90.00, 90.00[56] |
| 79 | $CsCu_5Se_3$ | O, Pmma(51) | 10.1783, 4.0949, 9.1844, 90.00, 90.00, 90.00 | 9.9909, 4.0978, 9.0071, 90.00, 90.00, 90.00[56] |
| 80 | $K_2Cu_2GeS_4$ | M, P 1 2 / c 1(13) | 7.1652, 5.3974, 11.2903, 90.00, 113.07, 90.00 | 7.0630, 5.4350, 11.0370, 90.00, 112.83, 90.00[57] |
| 81 | $Li_2In_2SiS_6$ | M, C1c1(9) | 12.2913, 7.1458, 12.2786, 90.00, 110.12, 90.00 | 12.0727, 7.0213, 12.0788, 90.00, 110.06, 90.00[58] |
| 82 | $Li_2In_2SiSe_6$ | M, C1c1(9) | 12.8785, 7.5352, 12.8327, 90.00, 109.86, 90.00 | 12.6210, 7.3788, 12.5800, 90.00, 109.70, 90.00[58] |
| 83 | $Li_2In_2GeS_6$ | M, C1c1(9) | 12.4239, 7.2505, 12.3802, 90.00, 110.10, 90.00 | 12.1650, 7.0840, 12.1310, 90.00, 110.26, 90.00[58] |
| 84 | $Li_2In_2GeSe_6$ | M, C1c1(9) | 12.9920, 7.6221, 12.9263, 90.00, 109.92, 90.00 | 12.7226, 7.4527, 12.6698, 90.00, 109.83, 90.00[58] |
| 85 | $Lu_5GaS_9$ | T, P-1(2) | 7.7911, 10.1081, 10.2244, 106.30, 100.29, 101.89 | 7.7350, 10.0330, 10.1200, 106.29, 100.18, 101.95[59] |
| 86 | $SrCdGeS_4$ | O, Ama2(40) | 10.5509, 10.4132, 6.5080, 90.00, 90.00, 90.00 | 10.3352, 10.2335, 6.4408, 90.00, 90.00, 90.00[60] |
| 87 | $SrCdGeSe_4$ | O, Ama2(40) | 11.0751, 10.9174, 6.7548, 90.00, 90.00, 90.00 | 10.8245, 10.6912, 6.6792, 90.00, 90.00, 90.00[60] |
| 88 | $TlInGe_2S_6$ | Tr, P3(143) | 10.1605, 10.1605, 9.2651, 90.00, 90.00, 120.00 | 9.7710, 9.7710, 8.9195, 90.00, 90.00, 120.00[61] |
| 89 | $TlInGe_2Se_6$ | Tr, P3(143) | 10.6304, 10.6304, 9.6646, 90.00, 90.00, 120.00 | 10.1798, 10.1798, 9.2872, 90.00, 90.00, 120.00[62] |
| 90 | $TlGaSn_2Se_6$ | Tr, R 3(146) | 10.6768, 10.6768, 9.7721, 90.00, 90.00, 119.99 | 10.3289, 10.3289, 9.4340, 90.00, 90.00, 120.00[63] |
| 91 | $Tl_2PbZrS_4$ | M, C12 /c1(15) | 15.5773, 8.2886, 6.8722, 90.00, 108.79, 90.00 | 15.4550, 8.2140, 6.7510, 90.00, 109.09, 90.00[64] |
| 92 | $Tl_2PbZrSe_4$ | M, C12 /c1(15) | 16.0778, 8.5776, 7.1514, 90.00, 108.75, 90.00 | 15.9060, 8.4720, 7.0160, 90.00, 109.01, 90.00[64] |
| 93 | $Tl_2PbHfS_4$ | M, C12 /c1(15) | 15.6171, 8.2820, 6.8136, 90.00, 108.90, 90.00 | 15.4700, 8.2260, 6.7103, 90.00, 109.26, 90.00[64] |
| 94 | $Tl_2PbHfSe_4$ | M, C12 /c1(15) | 16.1215, 8.5728, 7.0959, 90.00, 108.89, 90.00 | 15.9480, 8.4720, 6.9733, 90.00, 109.12, 90.00[64] |
| 95 | $Tl_2CdGeSe_4$ | Tet, I -4 2 m(121) | 8.1791, 8.1791, 6.8532, 90.00, 90.00, 90.00 | 8.0145, 8.0145, 6.7234, 90.00, 90.00, 90.00[65] |
| 96 | $Tl_2CdSnSe_4$ | Tet, I -4 2 m(121) | 8.2291, 8.2291, 6.9925, 90.00, 90.00, 90.00 | 8.0490, 8.0490, 6.8573, 90.00, 90.00, 90.00[65] |
| 97 | $Tl_2HgGeSe_4$ | Tet, I -4 2 m(121) | 8.1699, 8.1699, 6.9259, 90.00, 90.00, 90.00 | 7.9947, 7.9947, 6.7617, 90.00, 90.00, 90.00[66] |
| 98 | $Tl_2HgSiSe_4$ | Tet, I -4 2 m(121) | 8.1599, 8.1599, 6.8546, 90.00, 90.00, 90.00 | 8.0032, 8.0032, 6.6879, 90.00, 90.00, 90.00[67] |
| 99 | $Tl_2HgSnS_4$ | Tet, I -4 2 m(121) | 7.9589, 7.9589, 6.8578, 90.00, 90.00, 90.00 | 7.8571, 7.85716.6989, 90.00, 90.00, 90.00[68] |

**Table S2** The results for the energy gap ($E_g$). The third column is our results, while the fourth column is the other's results. (T) is for calculations and (E) for experimental results. (D) Stands for direct band gap, while (ID) stands for indirect band gap. The reference **74** represents the result from "materials project" website: https://materialsproject.org/
.

| # | Crystal | $E_g$(eV), our calculations | $E_g$(eV), other experimental and calculated data |
|---|---|---|---|
| 1 | $Cu_2ZnSiS_4$ | 1.380(D) | 1.370(D-T)[69] |
| 2 | $Cu_2ZnSiSe_4$ | 0.653(D) | 0.560(D-T)[69] |
| 3 | $Cu_2ZnSiTe_4$ | 0.483(ID) | 0.150(T)[70] |
| 4 | $Cu_2ZnGeS_4$ | 0.518(D) | 0.500(T)[71] |
| 5 | $Cu_2ZnGeSe_4$ | 0.125(D) | 0.060(T)[72], 0.640(T)[73] |
| 6 | $Cu_2ZnSnS_4$ | 0.390(D) | 0.030(T)[74] |
| 7 | $Cu_2ZnSnSe_4$ | 0.136(D) | 0.820(T)[75] |
| 8 | $Cu_2ZnSnTe_4$ | 0.324(ID) | 0.960(T)[12] |
| 9 | $Cu_2CdSiS_4$ | 1.233(D) | 0.980(T)[76] |
| 10 | **$Cu_2CdSiSe_4$** | 0.710(D) | —* |
| 11 | $Cu_2CdGeS_4$ | 0.541(D) | 0.640[77] |
| 12 | $Cu_2CdGeSe_4$ | 0.120(D) | 0.020(T)[16] |
| 13 | $Cu_2CdGeTe_4$ | 0.163(ID) | 0.000(T)[76] |
| 14 | $Cu_2CdSnS_4$ | 0.300(D) | 0.000(T)[78] |
| 15 | $Cu_2CdSnSe_4$ | 0.120(D) | 0.800(T)[79] |
| 16 | **$Cu_2CdSnTe_4$** | 0.267(ID) | —* |
| 17 | $Cu_2HgGeS_4$ | 0.400(D) | 0.307(T)[19] |
| 18 | $Cu_2HgGeSe_4$ | 0.005(D) | 0.006(T)[20] |
| 19 | $Cu_2HgGeTe_4$ | 0.151(ID) | 0.380(T)[80] |
| 20 | $Cu_2HgSnS_4$ | 0.103(D) | 0.191(T)[22] |
| 21 | $Cu_2HgSnSe_4$ | 0.000 | 0.127(T)[23], 0.0(T)[81] |
| 22 | $Cu_2HgSnTe_4$ | 0.432(D) | 0.390(T)[80] |
| 23 | $Cu_2BaGeS_4$ | 0.778(D) | 1.800(D-T)[82] |
| 24 | $Cu_2BaGeSe_4$ | 0.654(D) | 1.000(D-T)[82] |
| 25 | $Cu_2SrSiS_4$ | 2.200(D) | 3.30(ID-E)[27], 3.04(E)[83], 2.32(T)[83] |
| 26 | $Cu_2SrGeS_4$ | 1.184(D) | 1.050(T)[76] |
| 27 | $Cu_2SrGeSe_4$ | 0.880(ID) | 0.592(T)[76] |
| 28 | $Cu_2BaSnS_4$ | 0.632(D) | 1.460(T)[25] |
| 29 | $Cu_2BaSnSe_4$ | 0.930(D) | 1.500(D-T)[25] |
| 30 | $Cu_2SrSnS_4$ | 1.03(D) | 0.46(T)[28], 0.38(T)[84], 1.62(T)[84] |
| 31 | $Cu_2SrSnSe_4$ | 0.785(D) | 1.460(D-E)[25] |
| 32 | $Cu_2MgSiS_4$ | 1.689(D) | 2.010(T)[30] |
| 33 | $Cu_2MgSiSe_4$ | 1.047(D) | 1.017(T)[30] |
| 34 | $Cu_2MgGeS_4$ | 0.855(D) | 1.249(T)[30] |
| 35 | $Cu_2MgSnS_4$ | 0.626(D) | 0.781(T)[31], 0.35(T)[85] |
| 36 | $Cu_2MgSnSe_4$ | 0.381(D) | 0.000-0.580(T)[85] |
| 37 | **$Cu_2MgSnTe_4$** | 0.389(ID) | —* |
| 38 | $Ag_2ZnSiS_4$ | 0.463(D) | 1.800(D-T)[33] |
| 39 | $Ag_2ZnSnS_4$ | 0.750(D) | 1.150(D-T)[86] |
| 40 | $Ag_2ZnSnSe_4$ | 0.500(D) | 0.940(D-T)[87] |
| 41 | $Ag_2CdGeS_4$ | 0.998(D) | 1.060(T)[88] |
| 42 | $Ag_2CdSnS_4$ | 0.801(D) | 0.974(T)[36] |
| 43 | $Ag_2CdSnSe_4$ | 0.600(D) | 0.577(T)[37] |
| 44 | $Ag_2CdSnTe_4$ | 0.680(D) | 0.586(T)[37] |
| 45 | $Ag_2HgSnS_4$ | 0.528(D) | 0.170(T)[89] |
| 46 | $Ag_2HgSnSe_4$ | 0.310(D) | 0.484(T)[39], 0.23(T)[89] |
| 47 | $Ag_2BaGeS_4$ | 1.100(D) | 1.380(T)[25] |
| 48 | $Ag_2BaGeSe_4$ | 0.700(D) | 0.850(T)[25] |
| 49 | $Ag_2BaSnS_4$ | 1.010(D) | 1.260(T)[25] |
| 50 | $Ag_2BaSnSe_4$ | 0.810(D) | 0.770(T)[25] |

| | | | |
|---|---|---|---|
| 51 | $Ag_2SrGeS_4$ | 0.630(D) | 1.330(T)[25] |
| 52 | $Ag_2SrGeSe_4$ | 0.310(D) | 0.680(T)[25] |
| 53 | $Ag_2SrSnS_4$ | 0.550(D) | 1.080(T)[25] |
| 54 | $Ag_2SrSnSe_4$ | 0.410(D) | 0.660(T)[25] |
| 55 | $Ag_2In_2SiS_6$ | 1.404(D) | 1.570(T)[40] |
| 56 | $Ag_2In_2GeS_6$ | 1.333(D) | 1.880(T)[40] |
| 57 | $Ag_2In_2SiSe_6$ | 1.403(D) | 0.472(T)[76] |
| 58 | $Ag_2In_2GeSe_6$ | 1.040(D) | 0.432 (T)[76] |
| 59 | $Ag_2SiS_3$ | 1.600(ID) | 1.420(T)[42] |
| 60 | $Ag_2GeS_3$ | 0.663(ID) | 0.469(T)[76] |
| 61 | $Cu_2GeS_3$ | 0.025(ID) | 0.110(T)[76] |
| 62 | $Cu_2GeSe_3$ | 0.000 | 0.000(T)[90] |
| 63 | $Ag_2SnSe_3$ | 0.906(ID) | 0.059(T)[76] |
| 64 | **$Ag_2SnTe_3$** | 0.390(D) | **—*** |
| 65 | $Ag_4P_2S_6$ | 1.620(D) | 1.437(T)[76] |
| 66 | **$Ag_4P_2Se_6$** | 1.200(D) | **—*** |
| 67 | $BaZnSiSe_4$ | 2.494(D) | 2.710(T)[50] |
| 68 | $BaZnGeSe_4$ | 1.708(D) | 2.460(T)[50] |
| 69 | **$BaHg_2As_2S_6$** | 1.541(D) | **—*** |
| 70 | $BaCd_2As_2S_6$ | 1.377(D) | 1.770(D-T) |
| 71 | $Ba_2AlSbS_5$ | 2.069(D) | 2.400(ID-T)[52] |
| 72 | $Ba_2GaBiSe_5$ | 1.475(D) | 1.890(ID-T)[52] |
| 73 | $Ba_2AsGaSe_5$ | 2.031(D) | 1.862(T)[76] |
| 74 | **$Ba_2LaGaSe_5$** | 1.799(ID) | **—*** |
| 75 | **$Ba_2LuGaSe_5$** | 1.375(ID) | **—*** |
| 76 | **$Ba_2LuInSe_5$** | 1.244(D) | **—*** |
| 77 | $Ba_4AgInS_6$ | 1.956(D) | 1.686(T)[76] |
| 78 | $CsCu_5S_3$ | 0.390(D) | 0.325(T)[76] |
| 79 | $CsCu_5Se_3$ | 0.235(D) | 1.010(T)[56] |
| 80 | $K_2Cu_2GeS_4$ | 1.150(ID) | 1.457[91] |
| 81 | $Li_2In_2SiS_6$ | 2.062(D) | 3.240[58], 2.116(T)[76] |
| 82 | $Li_2In_2SiSe_6$ | 1.600(D) | 2.350[58], 1.497(T)[76] |
| 83 | $Li_2In_2GeS_6$ | 1.949(D) | 2.990[58], 2.033 (T)[76] |
| 84 | $Li_2In_2GeSe_6$ | 1.146(D) | 1.920[58], 1.199 (T)[76] |
| 85 | $Lu_5GaS_9$ | 1.759(D) | 1.890(ID-T)[59] |
| 86 | $SrCdGeS_4$ | 2.423(D) | 2.600(E)[60] |
| 87 | $SrCdGeSe_4$ | 1.777(D) | 1.900(E)[60] |
| 88 | $TlInGe_2S_6$ | 1.526(D) | 2.001(T)[61] |
| 89 | $TlInGe_2Se_6$ | 1.130(D) | 1.299(T)[62] |
| 90 | $TlGaSn_2Se_6$ | 1.100(D) | 1.143(T)[63] |
| 91 | $Tl_2PbZrS_4$ | 0.590(D) | 0.983(ID-T)[92], 1.240(T)[76] |
| 92 | $Tl_2PbZrSe_4$ | 0.470(D) | 0.529(ID-T)[92], 1.0190 (T)[76] |
| 93 | $Tl_2PbHfS_4$ | 0.580(D) | 0.911(ID-T)[92] |
| 94 | $Tl_2PbHfSe_4$ | 0.690(D) | 0.659(ID-T)[92], 0.962 (T)[76] |
| 95 | **$Tl_2CdGeSe_4$** | 0.000 | **—*** |
| 96 | **$Tl_2CdSnSe_4$** | 0.000 | **—*** |
| 97 | **$Tl_2HgGeSe_4$** | 0.000 | **—*** |
| 98 | $Tl_2HgSiSe_4$ | 0.000 | 0.959(T)[76] |
| 99 | **$Tl_2HgSnS_4$** | 0.000 | **—*** |

**Table S3** Our results for the 99 crystals: the average effective charge on each atoms (Q*), total bond order density (TBOD), and the plasma frequency ($\omega_p$). All numbers here are with three decimal point, except for TBOD, which have four significant figures.

| # | Crystal | $Q^*$ (in $e^-$) | TBOD ($e^-/Å^3$) | $\omega_p$(eV) |
|---|---|---|---|---|
| 1 | $Cu_2ZnSiS_4$ | 11.017(Cu), 11.611(Zn), 3.098(Si), 6.314(S) | 0.0248 | 19.500 |
| 2 | $Cu_2ZnSiSe_4$ | 11.039 (Cu), 11.703 (Zn), 3.368 (Si), 6.212 (Se) | 0.0205 | 18.100 |
| 3 | $Cu_2ZnSiTe_4$ | 11.262 (Cu), 11.932 (Zn), 3.788 (Si), 5.938 (Te) | 0.0180 | 17.250 |
| 4 | $Cu_2ZnGeS_4$ | 10.992 (Cu), 11.605 (Zn), 3.372 (Ge), 6.259 (S) | 0.0228 | 19.100 |
| 5 | $Cu_2ZnGeSe_4$ | 11.023 (Cu), 11.702 (Zn), 3.573 (Ge), 6.169 (Se) | 0.0189 | 18.100 |
| 6 | $Cu_2ZnSnS_4$ | 11.029 (Cu), 11.631 (Zn), 3.030 (Sn), 6.319 (S) | 0.0211 | 18.900 |
| 7 | $Cu_2ZnSnSe_4$ | 11.046 (Cu), 11.714 (Zn), 3.239 (Sn), 6.238 (Se) | 0.0180 | 18.100 |
| 8 | $Cu_2ZnSnTe_4$ | 11.258 (Cu), 11.954 (Zn), 3.555(Sn), 5.993 (Te) | 0.0163 | 16.800 |
| 9 | $Cu_2CdSiS_4$ | 11.078 (Cu), 11.138 (Cd), 3.092 (Si), 6.403 (S) | 0.0213 | 19.800 |
| 10 | $Cu_2CdSiSe_4$ | 11.077 (Cu), 11.194 (Cd), 3.368 (Si), 6.320 (Se) | 0.0177 | 18.100 |
| 11 | $Cu_2CdGeS_4$ | 11.046(Cu), 11.145 (Cd), 3.371 (Ge), 6.348 (S) | 0.0195 | 19.600 |
| 12 | $Cu_2CdGeSe_4$ | 11.061(Cu), 11.188 (Cd), 3.572(Ge), 6.279 (Se) | 0.0164 | 19.100 |
| 13 | $Cu_2CdGeTe_4$ | 11.264 (Cu), 11.289 (Cd), 3.925 (Ge), 6.064(Te) | 0.0147 | 17.100 |
| 14 | $Cu_2CdSnS_4$ | 11.065 (Cu), 11.161 (Cd), 3.070 (Sn), 6.409 (S) | 0.0181 | 19.100 |
| 15 | $Cu_2CdSnSe_4$ | 11.066 (Cu), 11.201 (Cd), 3.268 (Sn), 6.349 (Se) | 0.0155 | 17.400 |
| 16 | $Cu_2CdSnTe_4$ | 11.263 (Cu), 11.296 (Cd), 3.573 (Sn), 6.151(Te) | 0.0141 | 16.300 |
| 17 | $Cu_2HgGeS_4$ | 11.027 (Cu), 11.456(Hg), 3.316 (Ge), 6.293 (S) | 0.0132 | 20.100 |
| 18 | $Cu_2HgGeSe_4$ | 11.049(Cu), 11.551(Hg), 3.522(Ge), 6.207 (Se) | 0.0112 | 19.400 |
| 19 | $Cu_2HgGeTe_4$ | 11.316 (Cu), 11.816 (Hg), 3.883(Ge), 5.917 (Te) | 0.0103 | 17.100 |
| 20 | $Cu_2HgSnS_4$ | 11.045(Cu), 11.496(Hg), 3.056 (Sn), 6.339 (S) | 0.0126 | 19.100 |
| 21 | $Cu_2HgSnSe_4$ | 11.059 (Cu), 11.578 (Hg), 3.264 (Sn), 6.259 (Se) | 0.0109 | 18.500 |
| 22 | $Cu_2HgSnTe_4$ | 11.314 (Cu), 11.826 (Hg), 3.622 (Sn), 5.980 (Te) | 0.0102 | 16.500 |
| 23 | $Cu_2BaGeS_4$ | 11.200 (Cu), 8.103 (Ba), 3.367(Ge), 6.532 (S) | 0.0133 | 21.200 |
| 24 | $Cu_2BaGeSe_4$ | 11.199(Cu), 8.117(Ba), 3.544(Ge), 6.485 (Se) | 0.0123 | 20.500 |
| 25 | $Cu_2SrSiS_4$ | 11.168(Cu), 6.811(Sr), 3.084(Si), 6.442(S) | 0.0193 | 24.000 |
| 26 | $Cu_2SrGeS_4$ | 11.141 (Cu), 6.854 (Sr), 3.361 (Ge), 6.375 (S) | 0.0177 | 23.900 |
| 27 | $Cu_2SrGeSe_4$ | 11.138 (Cu), 6.891 (Sr), 3.541 (Ge), 6.322 (Se) | 0.0145 | 23.500 |
| 28 | $Cu_2BaSnS_4$ | 11.149 (Cu), 8.109(Ba), 3.136 (Sn), 6.614 (S) | 0.0110 | 19.900 |
| 29 | $Cu_2BaSnSe_4$ | 11.184 (Cu), 8.125 (Ba), 3.200 (Sn), 6.576 (Se) | 0.0111 | 20.100 |
| 30 | $Cu_2SrSnS_4$ | 11.176(Cu), 6.776(Sr), 3.041(Sn), 6.457(S) | 0.0159 | 23.100 |
| 31 | $Cu_2SrSnSe_4$ | 11.147 (Cu), 6.890 (Sr), 3.225 (Sn), 6.397 (Se) | 0.0145 | 23.500 |
| 32 | $Cu_2MgSiS_4$ | 11.069 (Cu), 6.973(Mg), 3.095 (Si), 6.448 (S) | 0.0224 | 19.400 |
| 33 | $Cu_2MgSiSe_4$ | 11.079 (Cu), 7.065(Mg), 3.363 (Si), 6.353 (Se) | 0.0186 | 18.200 |
| 34 | $Cu_2MgGeS_4$ | 11.054 (Cu), 6.983(Mg), 3.369 (Ge), 6.385(S) | 0.0206 | 19.400 |
| 35 | $Cu_2MgSnS_4$ | 11.076(Cu), 7.019 (Mg), 3.053 (Sn), 6.444(S) | 0.0191 | 18.100 |
| 36 | $Cu_2MgSnSe_4$ | 11.071 (Cu), 7.077 (Mg), 3.256 (Sn), 6.381 (Se) | 0.0162 | 16.900 |
| 37 | $Cu_2MgSnTe_4$ | 11.260 (Cu), 7.156 (Mg), 3.569 (Sn), 6.188 (Te) | 0.0143 | 16.100 |
| 38 | $Ag_2ZnSiS_4$ | 10.822 (Ag), 11.506(Zn), 3.133 (Si), 6.429 (S) | 0.0145 | 17.200 |
| 39 | $Ag_2ZnSnS_4$ | 10.939 (Ag), 11.604(Zn), 2.980 (Sn), 6.384 (S) | 0.0167 | 18.200 |
| 40 | $Ag_2ZnSnSe_4$ | 10.954 (Ag), 11.684(Zn), 3.192 (Sn), 6.303 (Se) | 0.0145 | 18.100 |
| 41 | $Ag_2CdGeS_4$ | 10.953 (Ag), 11.138 (Cd), 3.314 (Ge), 6.410 (S) | 0.0153 | 18.400 |
| 42 | $Ag_2CdSnS_4$ | 11.065 (Ag), 11.161 (Cd), 3.070 (Sn), 6.409 (S) | 0.0144 | 18.900 |
| 43 | $Ag_2CdSnSe_4$ | 10.973(Ag), 11.178 (Cd), 3.206(Sn), 6.417 (Se) | 0.0125 | 17.500 |
| 44 | $Ag_2CdSnTe_4$ | 11.100 (Ag), 11.278 (Cd), 3.533 (Sn), 6.247(Te) | 0.0115 | 16.800 |
| 45 | $Ag_2HgSnS_4$ | 10.950 (Ag), 11.470 (Hg), 2.98 (Sn), 6.412(S) | 0.0101 | 18.600 |
| 46 | $Ag_2HgSnSe_4$ | 10.965 (Ag), 11.546 (Hg), 3.198 (Sn), 6.331 (Se) | 0.0088 | 16.500 |
| 47 | $Ag_2BaGeS_4$ | 11.071 (Ag), 8.138 (Ba), 3.308 (Ge), 6.603(S) | 0.0120 | 20.900 |
| 48 | $Ag_2BaGeSe_4$ | 11.027 (Ag), 8.155(Ba), 3.494 (Ge), 6.574(Se) | 0.0100 | 20.100 |
| 49 | $Ag_2BaSnS_4$ | 11.035(Ag), 8.145 (Ba), 2.998 (Sn), 6.696 (S) | 0.0106 | 20.500 |
| 50 | $Ag_2BaSnSe_4$ | 11.018 (Ag), 8.157 (Ba), 3.169 (Sn), 6.659 (Se) | 0.0100 | 19.900 |
| 51 | $Ag_2SrGeS_4$ | 11.018 (Ag), 6.868(Sr), 3.318 (Ge), 6.444 (S) | 0.0144 | 23.500 |
| 52 | $Ag_2SrGeSe_4$ | 11.022 (Ag), 6.872(Sr), 3.515 (Ge), 6.392 (Se) | 0.0123 | 23.300 |
| 53 | $Ag_2SrSnS_4$ | 11.015 (Ag), 6.861 (Sr), 3.007 (Sn), 6.525 (S) | 0.0133 | 23.250 |

| | | | | |
|---|---|---|---|---|
| 54 | $Ag_2SrSnSe_4$ | 11.014(Ag), 6.866(Sr), 3.196 (Sn), 6.477 (Se) | 0.0110 | 23.700 |
| 55 | $Ag_2In_2SiS_6$ | 10.921 (Ag), 12.171 (In), 3.141(Si), 6.446(S) | 0.0159 | 17.400 |
| 56 | $Ag_2In_2GeS_6$ | 10.911 (Ag), 12.172 (In), 3.377(Ge), 6.409 (S) | 0.0149 | 17.500 |
| 57 | $Ag_2In_2SiSe_6$ | 10.951 (Ag), 12.317(In), 3.393(Si), 6.345 (Se) | 0.0137 | 17.100 |
| 58 | $Ag_2In_2GeSe_6$ | 10.940 (Ag), 12.314 (In), 3.588 (Ge), 6.317 (Se) | 0.0130 | 16.900 |
| 59 | $Ag_2SiS_3$ | 10.889(Ag), 3.042 (Si), 6.393 (S) | 0.0168 | 19.500 |
| 60 | $Ag_2GeS_3$ | 10.901 (Ag), 3.310 (Ge), 6.295 (S) | 0.0159 | 18.600 |
| 61 | $Cu_2GeS_3$ | 10.981 (Cu), 3.388 (Ge), 6.216 (S) | 0.0222 | 19.500 |
| 62 | $Cu_2GeSe_3$ | 11.017 (Cu), 3.591(Ge), 6.125(Se) | 0.0183 | 19.100 |
| 63 | $Ag_2SnSe_3$ | 10.921 (Ag), 3.496(Sn), 6.221(Se) | 0.0062 | 15.500 |
| 64 | $Ag_2SnTe_3$ | 11.089 (Ag), 3.529 (Sn), 6.097 (Te) | 0.0119 | 15.900 |
| 65 | $Ag_4P_2S_6$ | 10.888 (Ag), 4.338(P), S = 6.294 (S) | 0.0155 | 18.900 |
| 66 | $Ag_4P_2Se_6$ | 10.939 (Ag), 4.577(P), 6.182(Se) | 0.0134 | 18.700 |
| 67 | $BaZnSiSe_4$ | 8.160(Ba), 11.665 (Zn), 3.343(Si), 6.708(Se) | 0.0096 | 20.500 |
| 68 | $BaZnGeSe_4$ | 8.158 (Ba), 11.655 (Zn), 3.521 (Ge), 6.666 (Se) | 0.0088 | 20.200 |
| 69 | $BaHg_2As_2S_6$ | 8.815(Ba), 11.514 (Hg), 4.540 (As), 6.512 (S) | 0.0064 | 20.100 |
| 70 | $BaCd_2As_2S_6$ | 8.181(Ba), 11.164 (Cd), 4.559 (As), 6.729(S) | 0.0077 | 20.500 |
| 71 | $Ba_2AlSbS_5$ | 8.171 (Ba), 2.242 (Al), 4.246 (Sb), 7.033 (S) | 0.0063 | 21.300 |
| 72 | $Ba_2GaBiSe_5$ | 8.165 (Ba), 12.733 (Ga), 14.341(Bi), 6.919 (Se) | 0.0053 | 20.600 |
| 73 | $Ba_2AsGaSe_5$ | 8.171 (Ba), 4.644(As), 12.675 (Ga), 6.867 (Se) | 0.0054 | 20.900 |
| 74 | $Ba_2LaGaSe_5$ | 8.165 (Ba), 9.897(La), 12.683 (Ga), 7.018(Se) | 0.0065 | 22.900 |
| 75 | $Ba_2LuGaSe_5$ | 8.951 (Ba), 15.864(Lu), 12.737(Ga), 6.699 (Se) | 0.0061 | 20.500 |
| 76 | $Ba_2LuInSe_5$ | 8.989 (Ba), 15.902(Lu), 12.428 (In), 6.738 (Se) | 0.0118 | 20.450 |
| 77 | $Ba_4AgInS_6$ | 8.174 (Ba), 11.365 (Ag), 12.405 (In), 7.255 (S) | 0.0050 | 22.600 |
| 78 | $CsCu_5S_3$ | 8.212 (Cs), 11.012 (Cu), 6.242 (S) | 0.0181 | 20.700 |
| 79 | $CsCu_5Se_3$ | 8.287(Cs), 11.015 (Cu), 6.212(Se) | 0.0162 | 19.750 |
| 80 | $K_2Cu_2GeS_4$ | 6.254 (k), 11.091 (Cu), 3.419 (Ge), 6.472 (S) | 0.0136 | 24.500 |
| 81 | $Li_2In_2SiS_6$ | 0.669 (Li), 12.198 (In), 3.147 (Si), 6.519 (S) | 0.0167 | 17.500 |
| 82 | $Li_2In_2SiSe_6$ | 0.677 (Li), 12.336(In), 3.400 (Si), 6.428 (Se) | 0.0141 | 16.600 |
| 83 | $Li_2In_2GeS_6$ | 0.658 (Li), 12.197 (In), 3.390 (Ge), 6.483 (S) | 0.0155 | 17.400 |
| 84 | $Li_2In_2GeSe_6$ | 0.666 (Li), 12.331 (In), 3.604 (Ge), 6.399 (Se) | 0.0132 | 16.700 |
| 85 | $Lu_5GaS_9$ | 15.903 (Lu), 12.322(Ga), 6.684 (S) | 0.0102 | 31.100 |
| 86 | $SrCdGeS_4$ | 6.971 (Sr), 11.148(Cd), 3.354 (Ge), 6.631 (S) | 0.0121 | 23.900 |
| 87 | $SrCdGeSe_4$ | 6.965 (Sr), 11.203(Cd), 3.555 (Ge), 6.569 (Se) | 0.0105 | 23.500 |
| 88 | $TlInGe_2S_6$ | 12.619 (Tl), 12.171(In), 3.326 (Ge), 6.426 (S) | 0.0114 | 18.450 |
| 89 | $TlInGe_2Se_6$ | 12.649 (Tl), 12.312 (In), 3.552(Ge), 6.322 (Se) | 0.0095 | 17.900 |
| 90 | $TlGaSn_2Se_6$ | 12.711(Tl), 12.603 (Ga), 3.449 (Sn), 6.298(Se) | 0.0097 | 17.890 |
| 91 | $Tl_2PbZrS_4$ | 12.714 (Tl), 13.371 (Pb), 8.941 (Zr), 6.565(S) | 0.0075 | 18.200 |
| 92 | $Tl_2PbZrSe_4$ | 12.724(Tl), 13.427 (Pb), 9.027 (Zr), 6.524 (Se) | 0.0073 | 19.500 |
| 93 | $Tl_2PbHfS_4$ | 12.679 (Tl), 13.337 (Pb), 3.296 (Hf), 6.502 (S) | 0.0075 | 18.900 |
| 94 | $Tl_2PbHfSe_4$ | 12.681 (Tl), 13.387 (Pb), 3.388(Hf), 6.466(Se) | 0.0073 | 19.400 |
| 95 | $Tl_2CdGeSe_4$ | 12.743 (Tl), 11.166 (Cd), 3.545(Ge), 6.450 (Se) | 0.0085 | 18.600 |
| 96 | $Tl_2CdSnSe_4$ | 12.751 (Tl), 11.173(Cd), 3.236 (Sn), 6.529 (Se) | 0.0080 | 18.900 |
| 97 | $Tl_2HgGeSe_4$ | 12.649 (Tl), 11.535 (Hg), 3.485 (Ge), 6.420 (Se) | 0.0062 | 18.700 |
| 98 | $Tl_2HgSiSe_4$ | 12.660 (Tl), 11.544 (Hg), 3.344 (Si), 6.447 (Se) | 0.0067 | 18.450 |
| 99 | $Tl_2HgSnS_4$ | 12.632 (Tl), 11.498 (Hg), 2.993 (Sn), 6.561(S) | 0.0066 | 19.150 |

**Table S4** Our results for the 99 crystals: refractive index (n).

| # | Crystal | n |
|---|---|---|
| 1 | $Cu_2ZnSiS_4$ | 2.673 |
| 2 | $Cu_2ZnSiSe_4$ | 2.894 |
| 3 | $Cu_2ZnSiTe_4$ | 3.354 |
| 4 | $Cu_2ZnGeS_4$ | 2.915 |
| 5 | $Cu_2ZnGeSe_4$ | 3.741 |
| 6 | $Cu_2ZnSnS_4$ | 3.240 |
| 7 | $Cu_2ZnSnSe_4$ | 3.741 |
| 8 | $Cu_2ZnSnTe_4$ | 3.605 |
| 9 | $Cu_2CdSiS_4$ | 2.673 |
| 10 | $Cu_2CdSiSe_4$ | 2.932 |
| 11 | $Cu_2CdGeS_4$ | 3.016 |
| 12 | $Cu_2CdGeSe_4$ | 3.807 |
| 13 | $Cu_2CdGeTe_4$ | 3.821 |
| 14 | $Cu_2CdSnS_4$ | 3.391 |
| 15 | $Cu_2CdSnSe_4$ | 3.872 |
| 16 | $Cu_2CdSnTe_4$ | 3.674 |
| 17 | $Cu_2HgGeS_4$ | 3.162 |
| 18 | $Cu_2HgGeSe_4$ | 3.821 |
| 19 | $Cu_2HgGeTe_4$ | 3.847 |
| 20 | $Cu_2HgSnS_4$ | 3.566 |
| 21 | $Cu_2HgSnSe_4$ | 4.183 |
| 22 | $Cu_2HgSnTe_4$ | 3.549 |
| 23 | $Cu_2BaGeS_4$ | 2.915 |
| 24 | $Cu_2BaGeSe_4$ | 3.130 |
| 25 | $Cu_2SrSiS_4$ | 2.608 |
| 26 | $Cu_2SrGeS_4$ | 2.828 |
| 27 | $Cu_2SrGeSe_4$ | 3.049 |
| 28 | $Cu_2BaSnS_4$ | 2.881 |
| 29 | $Cu_2BaSnSe_4$ | 3.025 |
| 30 | $Cu_2SrSnS_4$ | 2.933 |
| 31 | $Cu_2SrSnSe_4$ | 3.098 |
| 32 | $Cu_2MgSiS_4$ | 2.588 |
| 33 | $Cu_2MgSiSe_4$ | 2.756 |
| 34 | $Cu_2MgGeS_4$ | 2.828 |
| 35 | $Cu_2MgSnS_4$ | 3.001 |
| 36 | $Cu_2MgSnSe_4$ | 3.354 |
| 37 | $Cu_2MgSnTe_4$ | 3.464 |
| 38 | $Ag_2ZnSiS_4$ | 2.692 |
| 39 | $Ag_2ZnSnS_4$ | 2.765 |
| 40 | $Ag_2ZnSnSe_4$ | 3.025 |
| 41 | $Ag_2CdGeS_4$ | 2.664 |
| 42 | $Ag_2CdSnS_4$ | 2.756 |
| 43 | $Ag_2CdSnSe_4$ | 2.983 |
| 44 | $Ag_2CdSnTe_4$ | 3.130 |
| 45 | $Ag_2HgSnS_4$ | 2.683 |
| 46 | $Ag_2HgSnSe_4$ | 2.983 |
| 47 | $Ag_2BaGeS_4$ | 2.262 |
| 48 | $Ag_2BaGeSe_4$ | 2.756 |
| 49 | $Ag_2BaSnS_4$ | 2.598 |
| 50 | $Ag_2BaSnSe_4$ | 2.738 |
| 51 | $Ag_2SrGeS_4$ | 2.626 |
| 52 | $Ag_2SrGeSe_4$ | 2.802 |
| 53 | $Ag_2SrSnS_4$ | 2.645 |
| 54 | $Ag_2SrSnSe_4$ | 2.766 |
| 55 | $Ag_2In_2SiS_6$ | 2.499 |
| 56 | $Ag_2In_2GeS_6$ | 2.549 |

| | | |
|---|---|---|
| 57 | $Ag_2In_2SiSe_6$ | 2.645 |
| 58 | $Ag_2In_2GeSe_6$ | 2.716 |
| 59 | $Ag_2SiS_3$ | 2.479 |
| 60 | $Ag_2GeS_3$ | 2.881 |
| 61 | $Cu_2GeS_3$ | 3.912 |
| 62 | $Cu_2GeSe_3$ | 3.982 |
| 63 | $Ag_2SnSe_3$ | 3.030 |
| 64 | $Ag_2SnTe_3$ | 3.718 |
| 65 | $Ag_4P_2S_6$ | 2.677 |
| 66 | $Ag_4P_2Se_6$ | 2.924 |
| 67 | $BaZnSiSe_4$ | 2.366 |
| 68 | $BaZnGeSe_4$ | 2.510 |
| 69 | $BaHg_2As_2S_6$ | 2.620 |
| 70 | $BaCd_2As_2S_6$ | 2.640 |
| 71 | $Ba_2AlSbS_5$ | 2.533 |
| 72 | $Ba_2GaBiSe_5$ | 2.885 |
| 73 | $Ba_2AsGaSe_5$ | 2.640 |
| 74 | $Ba_2LaGaSe_5$ | 2.706 |
| 75 | $Ba_2LuGaSe_5$ | 2.557 |
| 76 | $Ba_2LuInSe_5$ | 2.631 |
| 77 | $Ba_4AgInS_6$ | 2.506 |
| 78 | $CsCu_5S_3$ | 3.089 |
| 79 | $CsCu_5Se_3$ | 3.282 |
| 80 | $K_2Cu_2GeS_4$ | 2.549 |
| 81 | $Li_2In_2SiS_6$ | 2.330 |
| 82 | $Li_2In_2SiSe_6$ | 2.460 |
| 83 | $Li_2In_2GeS_6$ | 2.371 |
| 84 | $Li_2In_2GeSe_6$ | 2.527 |
| 85 | $Lu_5GaS_9$ | 2.542 |
| 86 | $SrCdGeS_4$ | 2.397 |
| 87 | $SrCdGeSe_4$ | 2.553 |
| 88 | $TlInGe_2S_6$ | 2.635 |
| 89 | $TlInGe_2Se_6$ | 2.787 |
| 90 | $TlGaSn_2Se_6$ | 2.569 |
| 91 | $Tl_2PbZrS_4$ | 4.375 |
| 92 | $Tl_2PbZrSe_4$ | 4.524 |
| 93 | $Tl_2PbHfS_4$ | 4.326 |
| 94 | $Tl_2PbHfSe_4$ | 4.424 |
| 95 | $Tl_2CdGeSe_4$ | 3.647 |
| 96 | $Tl_2CdSnSe_4$ | 3.535 |
| 97 | $Tl_2HgGeSe_4$ | 3.715 |
| 98 | $Tl_2HgSiSe_4$ | 3.701 |
| 99 | $Tl_2HgSnS_4$ | 3.605 |

**Table S5** The calculated elastic constants $C_{ij}$ (GPa) for the 99 crystals.

| # | Crystal | $C_{11}$ | $C_{12}$ | $C_{13}$ | $C_{21}$ | $C_{22}$ | $C_{23}$ | $C_{31}$ | $C_{32}$ | $C_{33}$ | $C_{44}$ | $C_{55}$ | $C_{66}$ |
|---|---|---|---|---|---|---|---|---|---|---|---|---|---|
| 1 | $Cu_2ZnSiS_4$ | 124.976 | 57.391 | 44.792 | 57.391 | 122.374 | 43.791 | 44.792 | 43.791 | 158.825 | 31.529 | 30.567 | 34.298 |
| 2 | $Cu_2ZnSiSe_4$ | 96.229 | 45.824 | 34.392 | 45.824 | 103.007 | 37.524 | 34.392 | 37.524 | 128.882 | 25.042 | 23.982 | 27.049 |
| 3 | $Cu_2ZnSiTe_4$ | 63.565 | 35.569 | 37.890 | 35.569 | 72.856 | 42.541 | 37.890 | 42.541 | 73.652 | 32.672 | 32.688 | 30.949 |
| 4 | $Cu_2ZnGeS_4$ | 120.211 | 52.512 | 40.167 | 52.512 | 116.010 | 37.896 | 40.166 | 37.896 | 149.271 | 29.284 | 28.284 | 32.728 |
| 5 | $Cu_2ZnGeSe_4$ | 77.649 | 47.234 | 44.566 | 47.234 | 86.378 | 48.803 | 44.566 | 48.803 | 75.521 | 35.650 | 35.570 | 33.268 |
| 6 | $Cu_2ZnSnS_4$ | 72.691 | 43.560 | 55.953 | 43.560 | 85.985 | 62.501 | 55.953 | 62.501 | 110.213 | 41.257 | 41.460 | 40.641 |
| 7 | $Cu_2ZnSnSe_4$ | 58.336 | 32.462 | 43.225 | 32.462 | 64.581 | 46.351 | 43.225 | 46.351 | 86.307 | 32.969 | 33.471 | 32.705 |
| 8 | $Cu_2ZnSnTe_4$ | 58.309 | 35.196 | 35.468 | 35.196 | 63.549 | 38.069 | 35.468 | 38.069 | 64.511 | 27.304 | 27.251 | 27.084 |
| 9 | $Cu_2CdSiS_4$ | 115.978 | 57.955 | 44.042 | 57.955 | 113.375 | 42.610 | 44.042 | 42.610 | 134.174 | 25.553 | 26.324 | 28.012 |
| 10 | $Cu_2CdSiSe_4$ | 92.815 | 45.688 | 34.498 | 45.688 | 87.940 | 31.211 | 34.498 | 31.211 | 106.086 | 20.252 | 21.125 | 22.874 |
| 11 | $Cu_2CdGeS_4$ | 107.377 | 53.835 | 45.122 | 53.835 | 107.520 | 45.241 | 45.122 | 45.241 | 137.512 | 23.802 | 23.980 | 26.313 |
| 12 | $Cu_2CdGeSe_4$ | 79.622 | 49.112 | 48.528 | 49.112 | 78.109 | 47.718 | 48.528 | 47.718 | 72.839 | 32.070 | 31.906 | 33.251 |
| 13 | $Cu_2CdGeTe_4$ | 61.859 | 33.689 | 37.224 | 33.689 | 56.851 | 34.725 | 37.224 | 34.725 | 61.191 | 25.808 | 26.211 | 26.277 |
| 14 | $Cu_2CdSnS_4$ | 81.601 | 49.905 | 56.101 | 49.905 | 78.827 | 54.768 | 56.101 | 54.768 | 89.189 | 35.515 | 35.381 | 34.104 |
| 15 | $Cu_2CdSnSe_4$ | 53.107 | 28.512 | 29.042 | 28.512 | 53.405 | 29.165 | 29.042 | 29.165 | 53.578 | 28.665 | 28.535 | 28.883 |
| 16 | $Cu_2CdSnTe_4$ | 53.240 | 31.697 | 41.200 | 31.697 | 55.045 | 42.130 | 41.200 | 42.130 | 74.027 | 24.182 | 24.100 | 23.996 |
| 17 | $Cu_2HgGeS_4$ | 91.607 | 41.997 | 34.980 | 41.997 | 62.280 | 20.251 | 34.980 | 20.251 | 44.574 | 38.184 | 38.072 | 41.502 |
| 18 | $Cu_2HgGeSe_4$ | 76.607 | 42.868 | 46.248 | 42.868 | 66.328 | 41.050 | 46.248 | 41.050 | 67.276 | 31.611 | 31.175 | 30.375 |
| 19 | $Cu_2HgGeTe_4$ | 59.697 | 35.159 | 33.704 | 35.159 | 61.822 | 34.719 | 33.704 | 34.719 | 54.490 | 26.612 | 26.509 | 27.334 |
| 20 | $Cu_2HgSnS_4$ | 92.442 | 65.655 | 64.542 | 65.655 | 95.905 | 66.146 | 64.542 | 66.146 | 94.226 | 33.419 | 33.155 | 35.502 |
| 21 | $Cu_2HgSnSe_4$ | 69.685 | 46.054 | 46.316 | 46.054 | 72.405 | 47.754 | 46.316 | 47.754 | 70.463 | 28.198 | 28.222 | 28.303 |
| 22 | $Cu_2HgSnTe_4$ | 56.820 | 34.403 | 34.799 | 34.403 | 56.934 | 34.881 | 34.799 | 34.881 | 56.881 | 25.068 | 24.708 | 24.928 |
| 23 | $Cu_2BaGeS_4$ | 91.526 | 57.293 | 35.981 | 57.293 | 85.189 | 33.136 | 35.981 | 33.136 | 45.259 | 14.519 | 14.432 | 16.261 |
| 24 | $Cu_2BaGeSe_4$ | 74.751 | 27.232 | 29.783 | 27.232 | 74.851 | 29.275 | 29.783 | 29.275 | 76.172 | 23.457 | 23.792 | 23.722 |
| 25 | $Cu_2SrSiS_4$ | 106.158 | 36.917 | 44.395 | 36.917 | 100.984 | 41.916 | 44.395 | 41.916 | 117.708 | 31.362 | 31.478 | 33.361 |
| 26 | $Cu_2SrGeS_4$ | 121.086 | 52.218 | 52.595 | 52.218 | 102.803 | 43.683 | 52.595 | 43.683 | 102.665 | 28.609 | 29.105 | 30.070 |
| 27 | $Cu_2SrGeSe_4$ | 86.694 | 28.535 | 29.741 | 28.535 | 57.957 | 13.409 | 29.741 | 13.409 | 83.557 | 17.348 | 23.242 | 19.489 |
| 28 | $Cu_2BaSnS_4$ | 54.238 | 27.629 | 24.297 | 27.629 | 33.311 | 13.575 | 24.297 | 13.575 | 27.860 | 10.552 | 9.874 | 3.574 |
| 29 | $Cu_2BaSnSe_4$ | 68.021 | 23.982 | 27.424 | 23.982 | 54.231 | 15.902 | 27.424 | 15.902 | 71.806 | 15.819 | 21.226 | 18.588 |
| 30 | $Cu_2SrSnS_4$ | 81.661 | 37.439 | 34.976 | 37.439 | 83.164 | 36.293 | 34.976 | 36.293 | 79.998 | 23.533 | 23.361 | 22.766 |
| 31 | $Cu_2SrSnSe_4$ | 69.817 | 26.140 | 19.786 | 26.140 | 55.530 | 10.355 | 19.786 | 10.355 | 72.686 | 15.669 | 20.656 | 17.804 |
| 32 | $Cu_2MgSiS_4$ | 118.587 | 53.850 | 50.725 | 53.850 | 102.415 | 40.535 | 50.725 | 40.535 | 135.986 | 25.646 | 26.055 | 28.408 |
| 33 | $Cu_2MgSiSe_4$ | 89.5117 | 40.327 | 37.097 | 40.327 | 83.675 | 33.952 | 37.097 | 33.9518 | 111.539 | 20.349 | 20.702 | 22.255 |
| 34 | $Cu_2MgGeS_4$ | 126.815 | 66.822 | 46.391 | 66.822 | 118.181 | 43.109 | 46.391 | 43.109 | 118.821 | 24.575 | 23.829 | 26.440 |
| 35 | $Cu_2MgSnS_4$ | 96.3197 | 62.230 | 55.544 | 62.230 | 88.115 | 51.446 | 55.544 | 51.446 | 75.628 | 36.222 | 36.388 | 35.120 |
| 36 | $Cu_2MgSnSe_4$ | 70.736 | 44.451 | 43.001 | 44.451 | 68.456 | 41.848 | 43.001 | 41.848 | 64.961 | 29.271 | 29.221 | 28.145 |
| 37 | $Cu_2MgSnTe_4$ | 60.0377 | 37.087 | 34.139 | 37.087 | 57.992 | 33.112 | 34.139 | 33.1118 | 51.7118 | 23.712 | 23.550 | 21.842 |
| 38 | $Ag_2ZnSiS_4$ | 53.457 | -22.437 | 42.503 | -22.437 | -2.748 | -10.656 | 42.503 | -10.656 | 76.139 | -2.695 | 25.667 | -2.784 |
| 39 | $Ag_2ZnSnS_4$ | 71.801 | 48.005 | 47.971 | 48.005 | 71.685 | 47.955 | 47.971 | 47.955 | 67.4917 | 27.074 | 26.925 | 28.778 |
| 40 | $Ag_2ZnSnSe_4$ | 61.230 | 40.581 | 40.641 | 40.581 | 61.196 | 40.623 | 40.641 | 40.623 | 57.131 | 22.924 | 23.187 | 23.730 |
| 41 | $Ag_2CdGeS_4$ | 76.828 | 42.406 | 38.791 | 42.406 | 71.311 | 36.552 | 38.791 | 36.552 | 92.942 | 16.185 | 14.349 | 16.290 |
| 42 | $Ag_2CdSnS_4$ | 63.466 | 44.086 | 45.333 | 44.086 | 63.341 | 45.326 | 45.333 | 45.326 | 62.837 | 22.994 | 23.163 | 23.059 |
| 43 | $Ag_2CdSnSe_4$ | 55.160 | 37.990 | 39.102 | 37.990 | 55.158 | 39.100 | 39.102 | 39.100 | 53.765 | 19.968 | 19.834 | 19.925 |
| 44 | $Ag_2CdSnTe_4$ | 47.132 | 31.145 | 32.097 | 31.145 | 47.116 | 32.107 | 32.097 | 32.107 | 45.540 | 17.551 | 17.517 | 16.093 |
| 45 | $Ag_2HgSnS_4$ | 68.560 | 40.264 | 34.727 | 40.264 | 69.749 | 36.083 | 34.727 | 36.083 | 85.812 | 13.058 | 12.766 | 14.130 |
| 46 | $Ag_2HgSnSe_4$ | 59.111 | 35.892 | 31.231 | 35.892 | 58.706 | 29.974 | 31.231 | 29.974 | 73.069 | 11.029 | 10.617 | 11.716 |
| 47 | $Ag_2BaGeS_4$ | 77.374 | 52.474 | 35.288 | 52.474 | 76.402 | 35.335 | 35.288 | 35.335 | 45.096 | 18.817 | 18.758 | 24.899 |
| 48 | $Ag_2BaGeSe_4$ | 76.896 | 31.395 | 24.057 | 31.395 | 34.235 | 19.477 | 24.057 | 19.477 | 39.674 | 12.256 | 13.807 | 16.255 |
| 49 | $Ag_2BaSnS_4$ | 36.370 | 19.794 | 28.966 | 19.794 | 43.647 | 23.611 | 28.966 | 23.611 | 79.775 | 15.707 | 15.190 | 12.780 |
| 50 | $Ag_2BaSnSe_4$ | 68.014 | 26.482 | 20.622 | 26.482 | 30.774 | 17.588 | 20.622 | 17.588 | 36.255 | 11.128 | 12.724 | 13.802 |
| 51 | $Ag_2SrGeS_4$ | 43.133 | 28.024 | 38.966 | 28.024 | 49.684 | 32.831 | 38.966 | 32.831 | 97.287 | 18.336 | 14.875 | 16.842 |
| 52 | $Ag_2SrGeSe_4$ | 80.985 | 33.469 | 26.381 | 33.469 | 34.559 | 23.170 | 26.381 | 23.170 | 42.890 | 12.774 | 14.141 | 13.827 |
| 53 | $Ag_2SrSnS_4$ | 31.386 | 36.637 | 28.514 | 36.637 | 120.002 | 40.945 | 28.514 | 40.945 | 85.624 | 13.888 | 11.926 | 10.520 |
| 54 | $Ag_2SrSnSe_4$ | 73.253 | 26.703 | 22.009 | 26.703 | 27.094 | 20.446 | 22.009 | 20.446 | 40.819 | 9.307 | 11.774 | 11.225 |
| 55 | $Ag_2In_2SiS_6$ | 62.397 | 24.651 | 18.747 | 24.651 | 48.765 | 21.433 | 18.747 | 21.433 | 54.904 | 16.189 | 18.491 | 15.286 |
| 56 | $Ag_2In_2GeS_6$ | 59.840 | 22.764 | 16.832 | 22.764 | 46.533 | 20.049 | 16.832 | 20.049 | 51.170 | 16.026 | 17.930 | 15.674 |
| 57 | $Ag_2In_2SiSe_6$ | 51.461 | 20.407 | 14.099 | 20.407 | 42.726 | 17.660 | 14.099 | 17.660 | 47.593 | 14.958 | 15.841 | 13.838 |
| 58 | $Ag_2In_2GeSe_6$ | 48.802 | 19.024 | 12.829 | 19.024 | 40.715 | 16.820 | 12.829 | 16.820 | 45.953 | 14.302 | 15.031 | 14.160 |
| 59 | $Ag_2SiS_3$ | 56.251 | 34.466 | 27.971 | 34.466 | 58.916 | 28.570 | 27.971 | 28.570 | 46.196 | 15.302 | 14.926 | 15.758 |
| 60 | $Ag_2GeS_3$ | 71.166 | 41.876 | 35.486 | 41.876 | 77.900 | 38.918 | 35.486 | 38.918 | 86.252 | 14.643 | 15.354 | 16.457 |

| | | | | | | | | | | | | | |
|---|---|---|---|---|---|---|---|---|---|---|---|---|---|
| 61 | $Cu_2GeS_3$ | 134.693 | 56.017 | 49.563 | 56.017 | 138.913 | 53.530 | 49.563 | 53.530 | 137.221 | 27.360 | 27.744 | 38.041 |
| 62 | $Cu_2GeSe_3$ | 90.258 | 22.124 | 33.831 | 22.124 | 91.791 | 45.794 | 33.831 | 45.794 | 70.491 | 35.640 | 36.984 | 17.619 |
| 63 | $Ag_2SnSe_3$ | 46.999 | 12.497 | -1.610 | 12.497 | 44.1259 | 0.256 | -1.610 | 0.256 | -1.121 | -0.159 | -0.120 | 4.707 |
| 64 | $Ag_2SnTe_3$ | 56.492 | 26.361 | 25.011 | 26.361 | 58.182 | 26.233 | 25.011 | 26.233 | 59.115 | 10.149 | 10.030 | 14.159 |
| 65 | $Ag_4P_2S_6$ | 37.452 | 23.542 | 13.658 | 23.542 | 51.270 | 23.162 | 13.658 | 23.162 | 48.369 | 11.005 | 8.009 | 13.303 |
| 66 | $Ag_4P_2Se_6$ | 31.154 | 18.140 | 15.169 | 18.140 | 46.173 | 20.972 | 15.169 | 20.972 | 62.375 | 11.283 | 8.404 | 13.372 |
| 67 | $BaZnSiSe_4$ | 40.965 | 16.652 | 14.171 | 16.652 | 40.530 | 14.486 | 14.171 | 14.486 | 64.844 | 15.783 | 11.062 | 11.318 |
| 68 | $BaZnGeSe_4$ | 39.497 | 16.506 | 13.042 | 16.506 | 39.480 | 13.947 | 13.042 | 13.947 | 61.662 | 14.608 | 9.338 | 10.987 |
| 69 | $BaHg_2As_2S_6$ | 46.118 | 17.648 | 25.198 | 17.648 | 32.077 | 19.779 | 25.198 | 19.779 | 47.645 | 8.514 | 10.991 | 15.820 |
| 70 | $BaCd_2As_2S_6$ | 46.680 | 16.588 | 28.578 | 16.588 | 34.653 | 20.911 | 28.578 | 20.911 | 54.494 | 11.812 | 13.559 | 13.439 |
| 71 | $Ba_2AlSbS_5$ | 56.807 | 13.313 | 12.537 | 13.313 | 57.296 | 21.717 | 12.537 | 21.717 | 41.783 | 23.407 | 15.138 | 12.243 |
| 72 | $Ba_2GaBiSe_5$ | 54.028 | 13.957 | 12.762 | 13.957 | 50.574 | 23.053 | 12.762 | 23.053 | 34.942 | 21.601 | 13.082 | 12.918 |
| 73 | $Ba_2AsGaSe_5$ | 47.541 | 9.792 | 12.274 | 9.792 | 44.902 | 17.968 | 12.274 | 17.968 | 38.743 | 20.964 | 12.079 | 10.432 |
| 74 | $Ba_2LaGaSe_5$ | 56.987 | 25.786 | 17.675 | 25.786 | 82.040 | 37.402 | 17.675 | 37.402 | 53.490 | 28.969 | 12.802 | 21.871 |
| 75 | $Ba_2LuGaSe_5$ | 56.099 | 21.670 | 20.891 | 21.670 | 67.588 | 17.551 | 20.891 | 17.551 | 38.863 | 19.413 | 16.909 | 23.118 |
| 76 | $Ba_2LuInSe_5$ | 79.547 | 20.711 | 25.759 | 20.711 | 46.703 | 26.135 | 25.759 | 26.135 | 54.520 | 17.584 | 25.982 | 21.249 |
| 77 | $Ba_4AgInS_6$ | 70.360 | 26.779 | 20.979 | 26.779 | 59.713 | 28.105 | 20.979 | 28.105 | 62.069 | 22.670 | 19.739 | 25.820 |
| 78 | $CsCu_5S_3$ | 89.317 | 30.515 | 30.104 | 30.515 | 93.144 | 22.175 | 30.104 | 22.175 | 44.843 | 9.642 | 10.110 | 20.477 |
| 79 | $CsCu_5Se_3$ | 50.747 | 23.266 | 18.869 | 23.266 | 76.543 | 13.073 | 18.869 | 13.073 | 44.015 | 5.641 | 9.501 | 12.384 |
| 80 | $K_2Cu_2GeS_4$ | 46.915 | 18.919 | 16.287 | 18.919 | 45.211 | 22.245 | 16.287 | 22.245 | 41.702 | 25.412 | 14.767 | 18.222 |
| 81 | $Li_2In_2SiS_6$ | 54.789 | 18.628 | 15.121 | 18.628 | 38.523 | 12.765 | 15.121 | 12.765 | 56.461 | 15.127 | 18.488 | 17.284 |
| 82 | $Li_2In_2SiSe_6$ | 44.302 | 16.361 | 11.831 | 16.361 | 31.895 | 10.733 | 11.831 | 10.733 | 47.676 | 13.175 | 15.257 | 14.784 |
| 83 | $Li_2In_2GeS_6$ | 51.774 | 17.779 | 14.309 | 17.779 | 37.755 | 12.434 | 14.309 | 12.434 | 54.835 | 14.871 | 17.995 | 16.915 |
| 84 | $Li_2In_2GeSe_6$ | 41.449 | 15.851 | 11.212 | 15.851 | 31.826 | 10.783 | 11.212 | 10.783 | 46.087 | 12.523 | 14.725 | 14.161 |
| 85 | $Lu_5GaS_9$ | 94.631 | 38.181 | 28.394 | 38.181 | 87.606 | 29.276 | 28.394 | 29.276 | 88.562 | 29.456 | 27.999 | 39.844 |
| 86 | $SrCdGeS_4$ | 50.802 | 18.578 | 22.073 | 18.579 | 59.205 | 19.702 | 22.073 | 19.702 | 83.01 | 22.488 | 15.569 | 12.785 |
| 87 | $SrCdGeSe_4$ | 44.558 | 15.534 | 19.281 | 15.534 | 48.720 | 17.101 | 19.281 | 17.101 | 67.284 | 20.065 | 12.742 | 9.143 |
| 88 | $TlInGe_2S_6$ | 26.336 | 9.936 | 7.368 | 9.936 | 27.018 | 7.252 | 7.368 | 7.252 | 24.577 | 10.493 | 10.558 | 8.967 |
| 89 | $TlInGe_2Se_6$ | 20.923 | 7.654 | 6.508 | 7.654 | 20.517 | 6.521 | 6.508 | 6.521 | 20.370 | 6.840 | 6.476 | 7.142 |
| 90 | $TlGaSn_2Se_6$ | 21.987 | 5.729 | 6.283 | 5.729 | 22.677 | 6.326 | 6.283 | 6.326 | 21.722 | 7.413 | 6.075 | 8.384 |
| 91 | $Tl_2PbZrS_4$ | 71.592 | 20.608 | 30.530 | 20.608 | 77.784 | 30.792 | 30.530 | 30.792 | 65.220 | 31.490 | 28.886 | 20.175 |
| 92 | $Tl_2PbZrSe_4$ | 62.568 | 18.418 | 26.385 | 18.418 | 69.103 | 25.800 | 26.385 | 25.800 | 54.044 | 29.239 | 28.127 | 18.590 |
| 93 | $Tl_2PbHfS_4$ | 73.009 | 21.428 | 30.886 | 21.428 | 79.663 | 31.599 | 30.886 | 31.599 | 66.162 | 31.080 | 30.297 | 19.067 |
| 94 | $Tl_2PbHfSe_4$ | 62.910 | 18.813 | 27.567 | 18.813 | 69.648 | 26.378 | 27.567 | 26.378 | 56.452 | 29.631 | 27.679 | 18.247 |
| 95 | $Tl_2CdGeSe_4$ | 35.066 | 16.506 | 13.086 | 16.506 | 35.053 | 13.110 | 13.086 | 13.110 | 65.206 | 7.475 | 7.586 | 7.795 |
| 96 | $Tl_2CdSnSe_4$ | 34.887 | 15.674 | 12.567 | 15.674 | 34.889 | 12.598 | 12.567 | 12.598 | 62.725 | 8.659 | 8.659 | 9.516 |
| 97 | $Tl_2HgGeSe_4$ | 33.585 | 17.241 | 12.772 | 17.241 | 33.632 | 12.799 | 12.772 | 12.799 | 61.386 | 6.186 | 6.156 | 8.609 |
| 98 | $Tl_2HgSiSe_4$ | 34.860 | 17.898 | 13.350 | 17.898 | 34.888 | 13.357 | 13.350 | 13.357 | 65.017 | 6.202 | 6.498 | 8.021 |
| 99 | $Tl_2HgSnS_4$ | 39.745 | 15.593 | 12.873 | 15.593 | 39.185 | 12.504 | 12.873 | 12.504 | 73.254 | 6.998 | 6.857 | 8.873 |

**Table S6** Calculated Young modulus (E), Bulk modulus (K), Shear modulus (G), Poisson's ratio (η), and Pugh's modulus ratio (G/K) for the 99 crystals.

| # | Crystal | E (GPa) | K (GPa) | G (GPa) | η | G/K |
|---|---|---|---|---|---|---|
| 1 | $Cu_2ZnSiS_4$ | 93.126 | 77.250 | 35.843 | 0.299 | 0.464 |
| 2 | $Cu_2ZnSiSe_4$ | 74.351 | 62.316 | 28.571 | 0.301 | 0.458 |
| 3 | $Cu_2ZnSiTe_4$ | 61.878 | 48.641 | 24.022 | 0.288 | 0.494 |
| 4 | $Cu_2ZnGeS_4$ | 88.466 | 71.463 | 34.191 | 0.293 | 0.478 |
| 5 | $Cu_2ZnGeSe_4$ | 66.938 | 56.607 | 25.688 | 0.302 | 0.454 |
| 6 | $Cu_2ZnSnS_4$ | 74.818 | 60.896 | 28.882 | 0.295 | 0.474 |
| 7 | $Cu_2ZnSnSe_4$ | 60.584 | 48.061 | 23.484 | 0.290 | 0.488 |
| 8 | $Cu_2ZnSnTe_4$ | 52.559 | 44.463 | 20.169 | 0.303 | 0.454 |
| 9 | $Cu_2CdSiS_4$ | 75.925 | 64.650 | 29.106 | 0.304 | 0.450 |
| 10 | $Cu_2CdSiSe_4$ | 63.029 | 56.323 | 23.993 | 0.314 | 0.426 |
| 11 | $Cu_2CdGeS_4$ | 74.031 | 70.103 | 27.957 | 0.324 | 0.399 |
| 12 | $Cu_2CdGeSe_4$ | 61.468 | 57.525 | 23.250 | 0.322 | 0.404 |
| 13 | $Cu_2CdGeTe_4$ | 50.368 | 42.912 | 19.307 | 0.304 | 0.449 |
| 14 | $Cu_2CdSnS_4$ | 65.538 | 62.530 | 24.725 | 0.325 | 0.395 |
| 15 | $Cu_2CdSnSe_4$ | 50.871 | 34.987 | 20.225 | 0.258 | 0.578 |
| 16 | $Cu_2CdSnTe_4$ | 46.157 | 42.952 | 17.472 | 0.321 | 0.407 |
| 17 | $Cu_2HgGeS_4$ | 64.026 | 36.019 | 26.595 | 0.204 | 0.738 |
| 18 | $Cu_2HgGeSe_4$ | 57.736 | 50.952 | 22.018 | 0.311 | 0.432 |
| 19 | $Cu_2HgGeTe_4$ | 50.546 | 42.245 | 19.432 | 0.301 | 0.459 |
| 20 | $Cu_2HgSnS_4$ | 64.868 | 72.857 | 23.996 | 0.351 | 0.329 |
| 21 | $Cu_2HgSnSe_4$ | 53.615 | 54.369 | 20.071 | 0.335 | 0.369 |
| 22 | $Cu_2HgSnTe_4$ | 47.229 | 41.868 | 17.999 | 0.312 | 0.429 |
| 23 | $Cu_2BaGeS_4$ | 40.178 | 46.784 | 14.805 | 0.357 | 0.316 |
| 24 | $Cu_2BaGeSe_4$ | 59.645 | 43.776 | 23.428 | 0.273 | 0.535 |
| 25 | $Cu_2SrSiS_4$ | 83.363 | 63.148 | 32.564 | 0.280 | 0.516 |
| 26 | $Cu_2SrGeS_4$ | 76.908 | 68.357 | 29.299 | 0.313 | 0.429 |
| 27 | $Cu_2SrGeSe_4$ | 55.519 | 39.820 | 21.899 | 0.268 | 0.549 |
| 28 | $Cu_2BaSnS_4$ | 12.787 | 17.056 | 4.650 | 0.375 | 0.273 |
| 29 | $Cu_2BaSnSe_4$ | 49.115 | 35.858 | 19.311 | 0.272 | 0.538 |
| 30 | $Cu_2SrSnS_4$ | 60.025 | 51.313 | 22.997 | 0.305 | 0.448 |
| 31 | $Cu_2SrSnSe_4$ | 49.689 | 33.991 | 19.775 | 0.256 | 0.582 |
| 32 | $Cu_2MgSiS_4$ | 78.047 | 71.424 | 29.611 | 0.317 | 0.414 |
| 33 | $Cu_2MgSiSe_4$ | 62.347 | 55.851 | 23.725 | 0.314 | 0.425 |
| 34 | $Cu_2MgGeS_4$ | 74.524 | 72.668 | 28.036 | 0.329 | 0.386 |
| 35 | $Cu_2MgSnS_4$ | 67.158 | 65.208 | 25.278 | 0.328 | 0.387 |
| 36 | $Cu_2MgSnSe_4$ | 54.339 | 50.579 | 20.568 | 0.320 | 0.406 |
| 37 | $Cu_2MgSnTe_4$ | 44.901 | 41.570 | 17.008 | 0.320 | 0.409 |
| 38 | $Ag_2ZnSiS_4$ | 34.698 | 21.450 | 14.100 | 0.230 | 0.657 |
| 39 | $Ag_2ZnSnS_4$ | 51.529 | 54.941 | 19.175 | 0.352 | 0.349 |
| 40 | $Ag_2ZnSnSe_4$ | 43.804 | 46.639 | 16.303 | 0.343 | 0.349 |
| 41 | $Ag_2CdGeS_4$ | 46.640 | 52.740 | 17.241 | 0.353 | 0.327 |
| 42 | $Ag_2CdSnS_4$ | 43.192 | 50.553 | 15.908 | 0.358 | 0.315 |
| 43 | $Ag_2CdSnSe_4$ | 37.396 | 43.665 | 13.776 | 0.357 | 0.315 |
| 44 | $Ag_2CdSnTe_4$ | 32.876 | 36.491 | 32.876 | 0.350 | 0.901 |
| 45 | $Ag_2HgSnS_4$ | 41.254 | 49.446 | 15.156 | 0.360 | 0.306 |
| 46 | $Ag_2HgSnSe_4$ | 34.389 | 42.686 | 12.590 | 0.365 | 0.295 |
| 47 | $Ag_2BaGeS_4$ | 44.575 | 46.035 | 16.650 | 0.339 | 0.362 |
| 48 | $Ag_2BaGeSe_4$ | 33.695 | 30.678 | 12.793 | 0.317 | 0.417 |
| 49 | $Ag_2BaSnS_4$ | 36.533 | 31.614 | 13.972 | 0.307 | 0.442 |
| 50 | $Ag_2BaSnSe_4$ | 30.550 | 27.316 | 11.628 | 0.314 | 0.426 |
| 51 | $Ag_2SrGeS_4$ | 40.220 | 40.102 | 15.088 | 0.333 | 0.376 |
| 52 | $Ag_2SrGeSe_4$ | 32.853 | 33.213 | 12.303 | 0.335 | 0.370 |
| 53 | $Ag_2SrSnS_4$ | 37.847 | 39.987 | 14.098 | 0.342 | 0.352 |
| 54 | $Ag_2SrSnSe_4$ | 27.609 | 28.254 | 10.324 | 0.337 | 0.365 |

| | | | | | | |
|---|---|---|---|---|---|---|
| 55 | $Ag_2In_2SiS_6$ | 42.267 | 32.698 | 16.452 | 0.284 | 0.503 |
| 56 | $Ag_2In_2GeS_6$ | 41.125 | 30.522 | 16.122 | 0.275 | 0.528 |
| 57 | $Ag_2In_2SiSe_6$ | 37.318 | 27.264 | 14.670 | 0.272 | 0.538 |
| 58 | $Ag_2In_2GeSe_6$ | 36.026 | 25.737 | 14.220 | 0.266 | 0.552 |
| 59 | $Ag_2SiS_3$ | 36.151 | 37.137 | 13.512 | 0.338 | 0.364 |
| 60 | $Ag_2GeS_3$ | 45.946 | 51.853 | 16.988 | 0.352 | 0.327 |
| 61 | $Cu_2GeS_3$ | 86.215 | 78.050 | 32.759 | 0.316 | 0.419 |
| 62 | $Cu_2GeSe_3$ | 66.695 | 49.809 | 26.117 | 0.277 | 0.524 |
| 63 | $Ag_2SnSe_3$ | 7.983 | 6.334 | 3.094 | 0.290 | 0.488 |
| 64 | $Ag_2SnTe_3$ | 32.831 | 36.275 | 12.167 | 0.349 | 0.335 |
| 65 | $Ag_4P_2S_6$ | 28.793 | 26.883 | 10.894 | 0.321 | 0.405 |
| 66 | $Ag_4P_2Se_6$ | 30.814 | 26.386 | 11.803 | 0.305 | 0.447 |
| 67 | $BaZnSiSe_4$ | 35.352 | 25.914 | 13.889 | 0.272 | 0.536 |
| 68 | $BaZnGeSe_4$ | 33.095 | 24.950 | 12.939 | 0.279 | 0.518 |
| 69 | $BaHg_2As_2S_6$ | 29.009 | 27.051 | 10.978 | 0.312 | 0.406 |
| 70 | $BaCd_2As_2S_6$ | 32.183 | 28.554 | 12.263 | 0.312 | 0.429 |
| 71 | $Ba_2AlSbS_5$ | 41.785 | 27.607 | 16.744 | 0.247 | 0.606 |
| 72 | $Ba_2GaBiSe_5$ | 37.221 | 26.119 | 14.741 | 0.262 | 0.564 |
| 73 | $Ba_2AsGaSe_5$ | 35.469 | 23.427 | 14.214 | 0.247 | 0.607 |
| 74 | $Ba_2LaGaSe_5$ | 49.132 | 37.689 | 19.151 | 0.282 | 0.508 |
| 75 | $Ba_2LuGaSe_5$ | 44.828 | 30.228 | 17.890 | 0.252 | 0.592 |
| 76 | $Ba_2LuInSe_5$ | 49.157 | 35.308 | 19.384 | 0.268 | 0.549 |
| 77 | $Ba_4AgInS_6$ | 53.439 | 38.170 | 21.094 | 0.266 | 0.553 |
| 78 | $CsCu_5S_3$ | 42.324 | 40.648 | 15.954 | 0.326 | 0.392 |
| 79 | $CsCu_5Se_3$ | 33.026 | 30.929 | 12.490 | 0.322 | 0.404 |
| 80 | $K_2Cu_2GeS_4$ | 37.130 | 25.715 | 14.741 | 0.259 | 0.573 |
| 81 | $Li_2In_2SiS_6$ | 41.436 | 26.432 | 16.725 | 0.238 | 0.633 |
| 82 | $Li_2In_2SiSe_6$ | 34.625 | 22.032 | 13.983 | 0.238 | 0.635 |
| 83 | $Li_2In_2GeS_6$ | 40.268 | 25.409 | 16.291 | 0.235 | 0.641 |
| 84 | $Li_2In_2GeSe_6$ | 33.268 | 21.337 | 13.413 | 0.240 | 0.629 |
| 85 | $Lu_5GaS_9$ | 75.751 | 49.729 | 30.394 | 0.246 | 0.611 |
| 86 | $SrCdGeS_4$ | 46.681 | 34.140 | 18.347 | 0.272 | 0.537 |
| 87 | $SrCdGeSe_4$ | 38.147 | 28.854 | 14.905 | 0.279 | 0.517 |
| 88 | $TlInGe_2S_6$ | 21.375 | 13.930 | 8.589 | 0.244 | 0.617 |
| 89 | $TlInGe_2Se_6$ | 14.097 | 10.891 | 5.488 | 0.284 | 0.504 |
| 90 | $TlGaSn_2Se_6$ | 16.406 | 11.207 | 6.531 | 0.256 | 0.583 |
| 91 | $Tl_2PbZrS_4$ | 59.137 | 41.681 | 23.401 | 0.263 | 0.561 |
| 92 | $Tl_2PbZrSe_4$ | 52.858 | 35.969 | 21.057 | 0.255 | 0.585 |
| 93 | $Tl_2PbHfS_4$ | 59.568 | 42.615 | 23.507 | 0.267 | 0.552 |
| 94 | $Tl_2PbHfSe_4$ | 53.300 | 36.853 | 21.168 | 0.259 | 0.574 |
| 95 | $Tl_2CdGeSe_4$ | 26.123 | 23.841 | 9.915 | 0.317 | 0.416 |
| 96 | $Tl_2CdSnSe_4$ | 28.141 | 23.231 | 10.839 | 0.298 | 0.466 |
| 97 | $Tl_2HgGeSe_4$ | 23.976 | 23.190 | 9.0294 | 0.328 | 0.389 |
| 98 | $Tl_2HgSiSe_4$ | 24.498 | 24.184 | 9.202 | 0.331 | 0.380 |
| 99 | $Tl_2HgSnS_4$ | 28.160 | 25.140 | 10.721 | 0.313 | 0.426 |

## 4. The Supplementary Figures

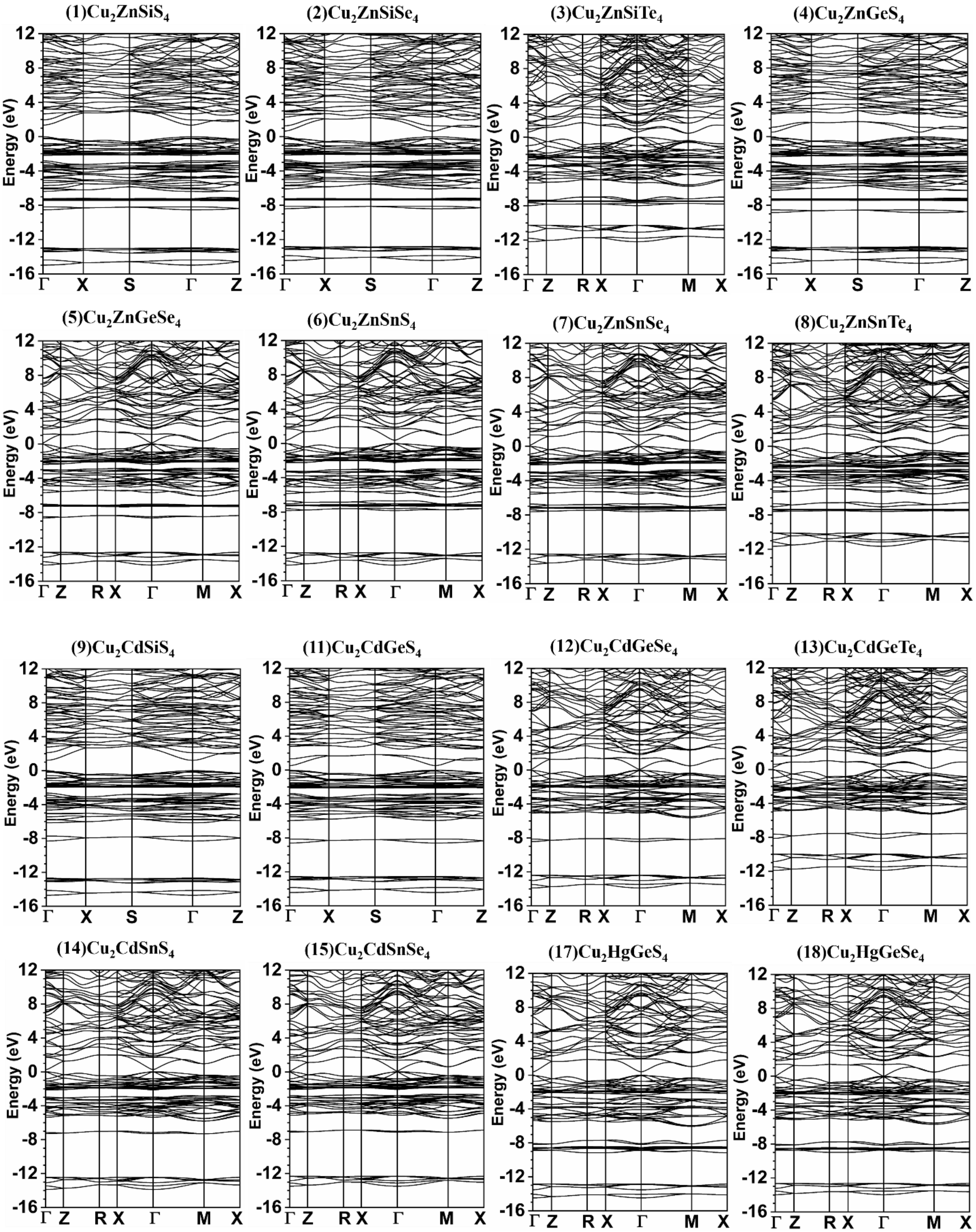

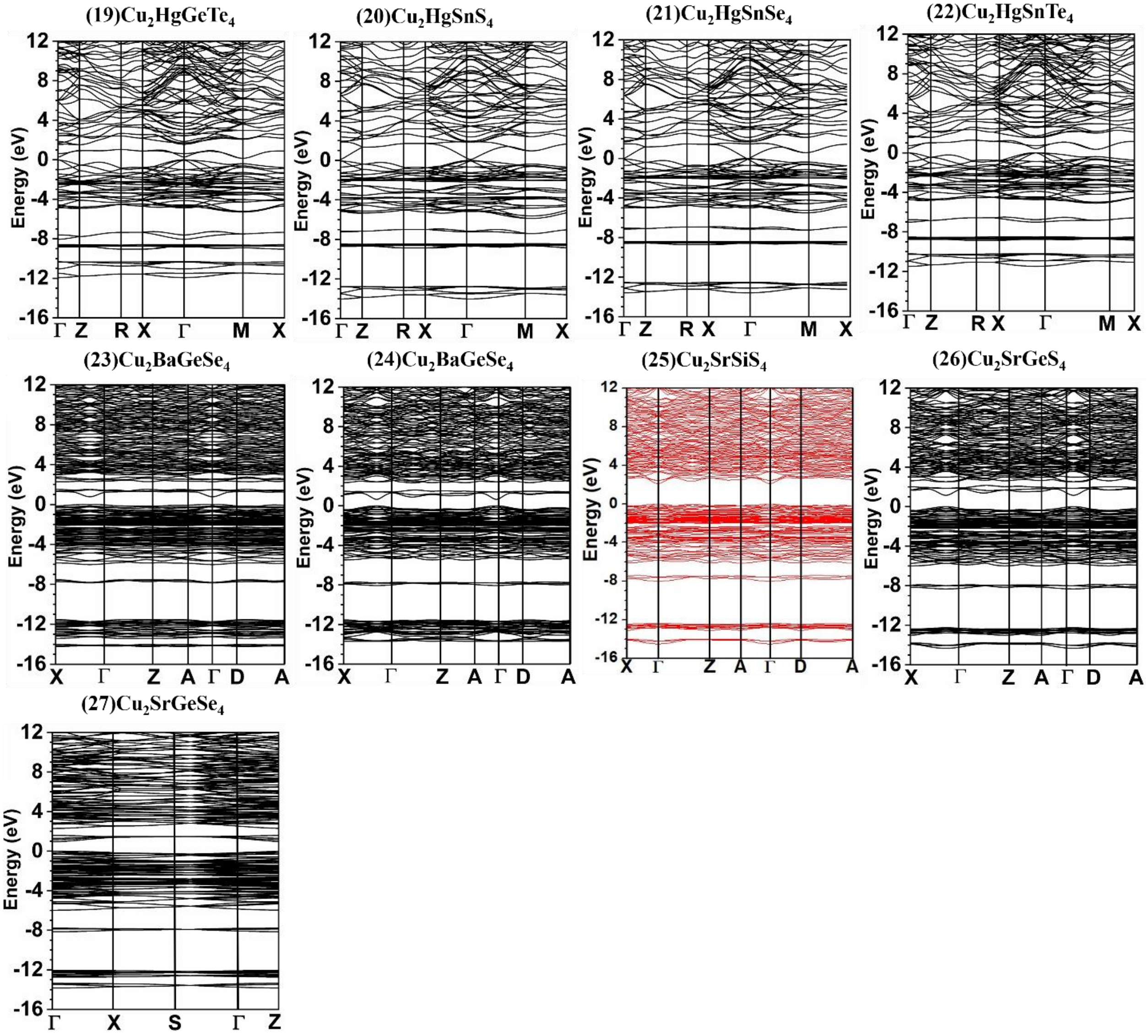
(19)Cu2HgGeTe4
(20)Cu2HgSnS4
(21)Cu2HgSnSe4
(22)Cu2HgSnTe4
(23)Cu2BaGeSe4
(24)Cu2BaGeSe4
(25)Cu2SrSiS4
(26)Cu2SrGeS4
(27)Cu2SrGeSe4
Energy (eV)
12
8
4
0
-4
-8
-12
-16
Γ Z R X Γ M X
X Γ Z A Γ D A
Γ X S Γ Z

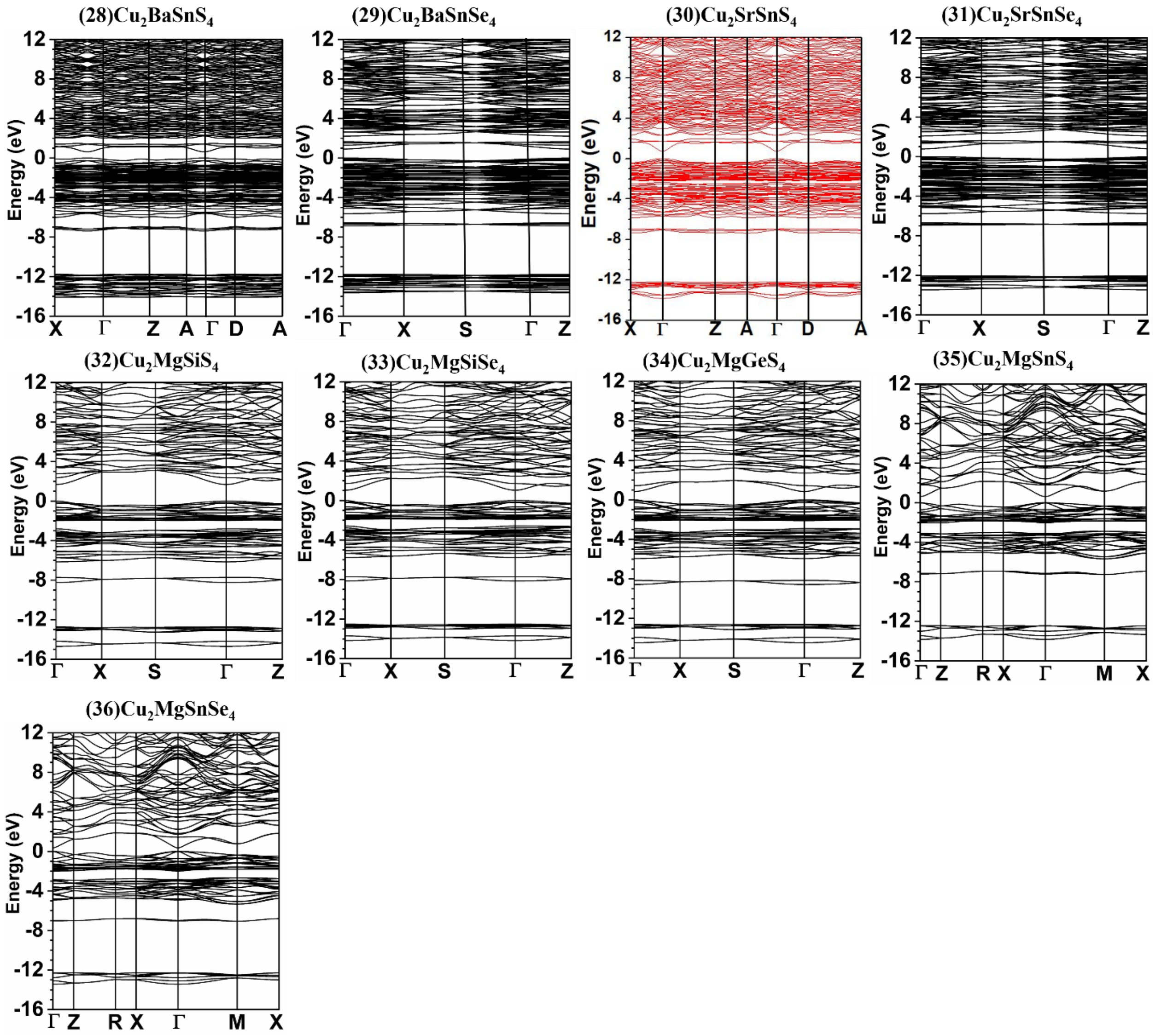
(28)$Cu_2BaSnS_4$
(29)$Cu_2BaSnSe_4$
(30)$Cu_2SrSnS_4$
(31)$Cu_2SrSnSe_4$
(32)$Cu_2MgSiS_4$
(33)$Cu_2MgSiSe_4$
(34)$Cu_2MgGeS_4$
(35)$Cu_2MgSnS_4$
(36)$Cu_2MgSnSe_4$
Energy (eV)
12
8
4
0
-4
-8
-12
-16
X Γ Z A Γ D A
Γ X S Γ Z
Γ Z R X Γ M X

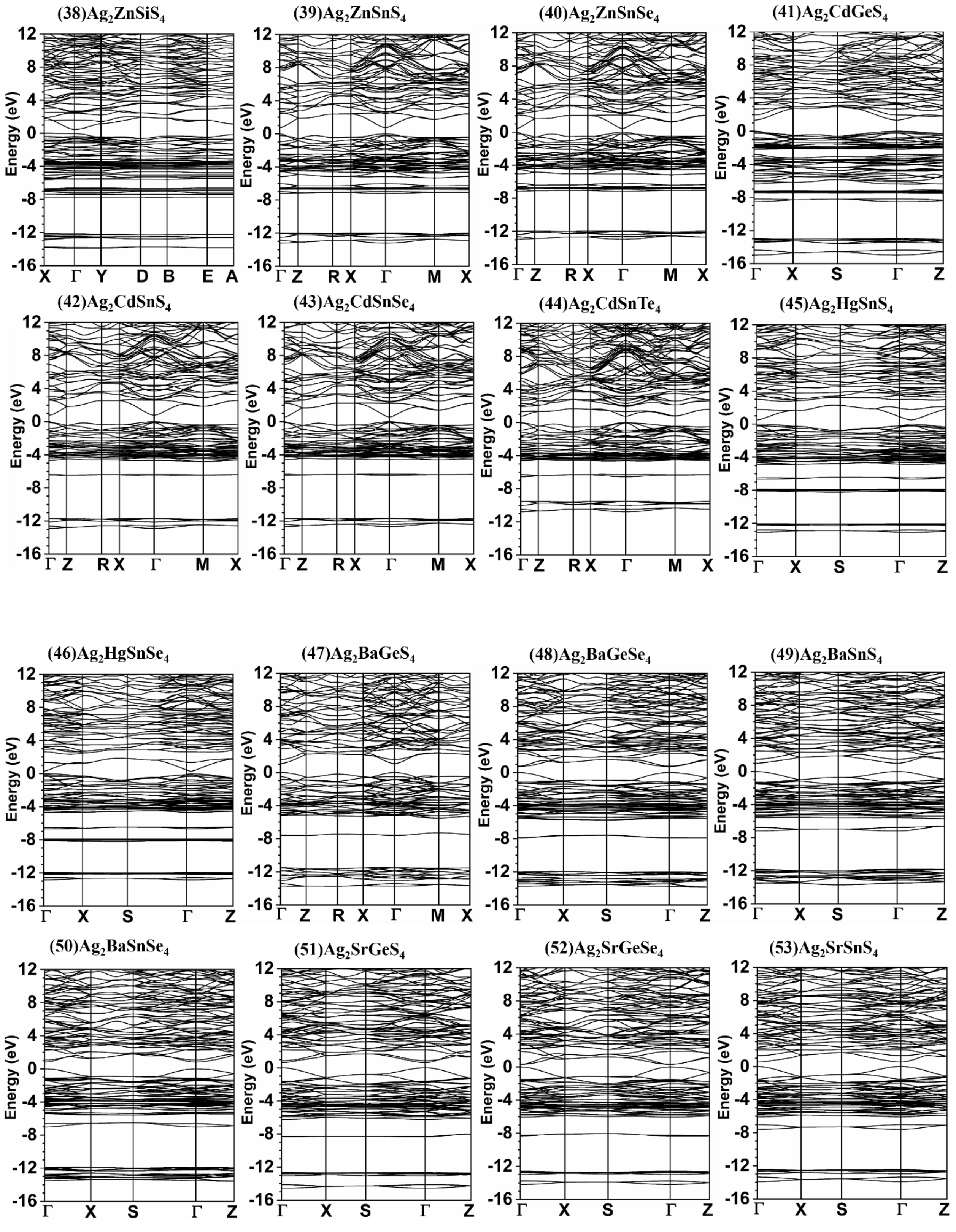
(38)$Ag_2ZnSiS_4$
(39)$Ag_2ZnSnS_4$
(40)$Ag_2ZnSnSe_4$
(41)$Ag_2CdGeS_4$
(42)$Ag_2CdSnS_4$
(43)$Ag_2CdSnSe_4$
(44)$Ag_2CdSnTe_4$
(45)$Ag_2HgSnS_4$
(46)$Ag_2HgSnSe_4$
(47)$Ag_2BaGeS_4$
(48)$Ag_2BaGeSe_4$
(49)$Ag_2BaSnS_4$
(50)$Ag_2BaSnSe_4$
(51)$Ag_2SrGeS_4$
(52)$Ag_2SrGeSe_4$
(53)$Ag_2SrSnS_4$
Energy (eV)
X Γ Y D B E A
Γ Z R X Γ M X
Γ X S Γ Z

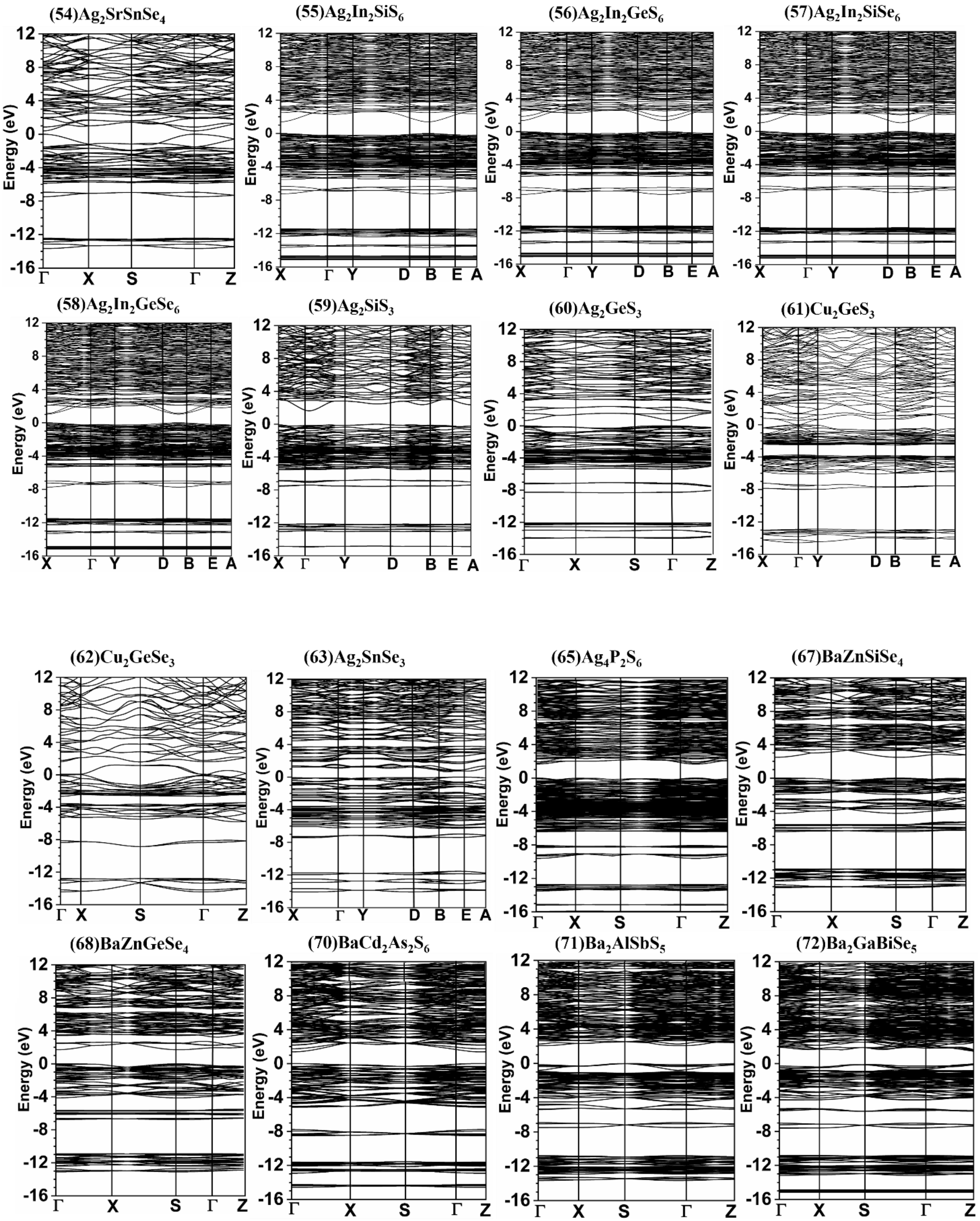
(54)$Ag_2SrSnSe_4$
(55)$Ag_2In_2SiS_6$
(56)$Ag_2In_2GeS_6$
(57)$Ag_2In_2SiSe_6$
(58)$Ag_2In_2GeSe_6$
(59)$Ag_2SiS_3$
(60)$Ag_2GeS_3$
(61)$Cu_2GeS_3$
(62)$Cu_2GeSe_3$
(63)$Ag_2SnSe_3$
(65)$Ag_4P_2S_6$
(67)$BaZnSiSe_4$
(68)$BaZnGeSe_4$
(70)$BaCd_2As_2S_6$
(71)$Ba_2AlSbS_5$
(72)$Ba_2GaBiSe_5$
Energy (eV)

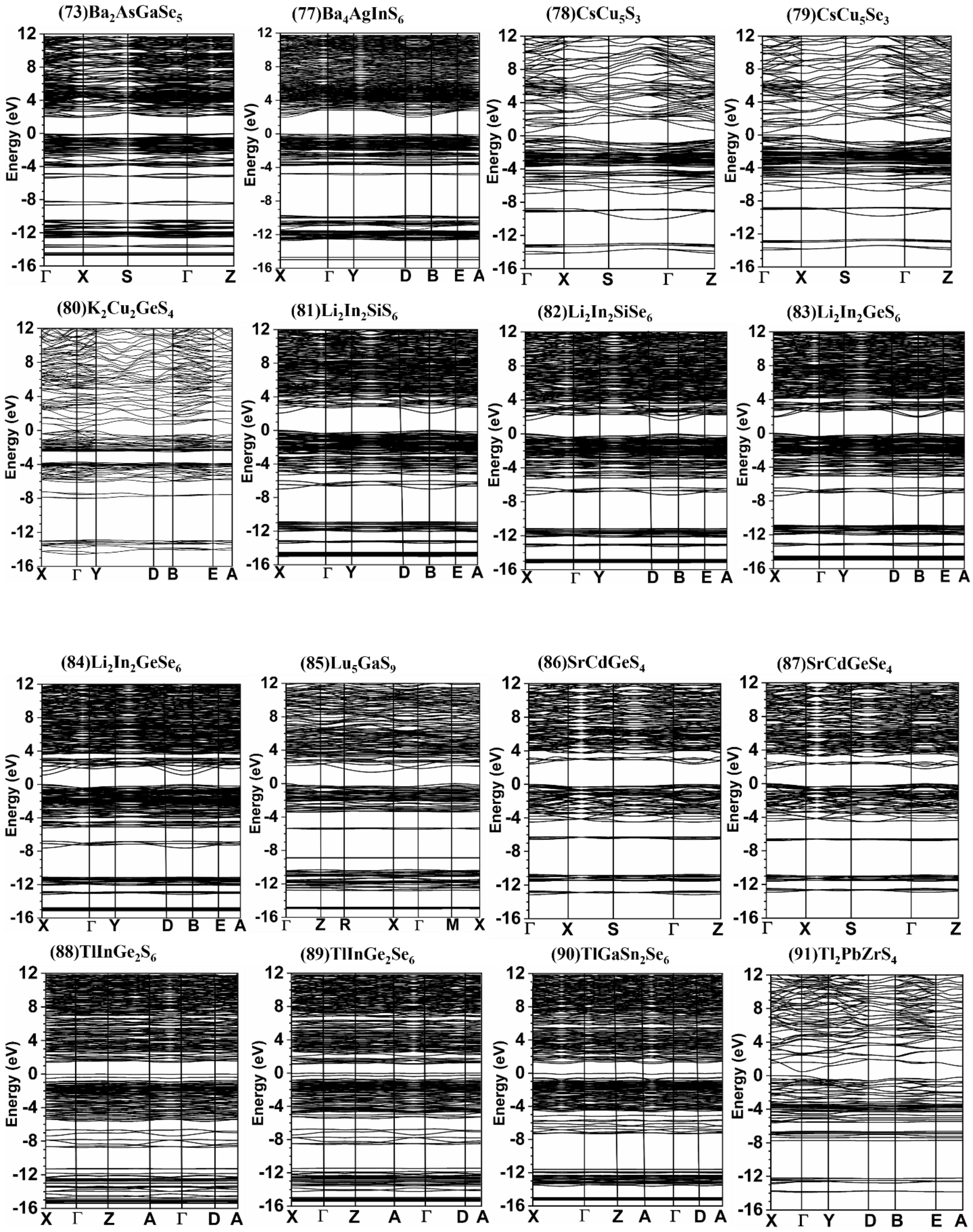

(73)$Ba_2AsGaSe_5$
(77)$Ba_4AgInS_6$
(78)$CsCu_5S_3$
(79)$CsCu_5Se_3$
(80)$K_2Cu_2GeS_4$
(81)$Li_2In_2SiS_6$
(82)$Li_2In_2SiSe_6$
(83)$Li_2In_2GeS_6$
(84)$Li_2In_2GeSe_6$
(85)$Lu_5GaS_9$
(86)$SrCdGeS_4$
(87)$SrCdGeSe_4$
(88)$TlInGe_2S_6$
(89)$TlInGe_2Se_6$
(90)$TlGaSn_2Se_6$
(91)$Tl_2PbZrS_4$
Energy (eV)

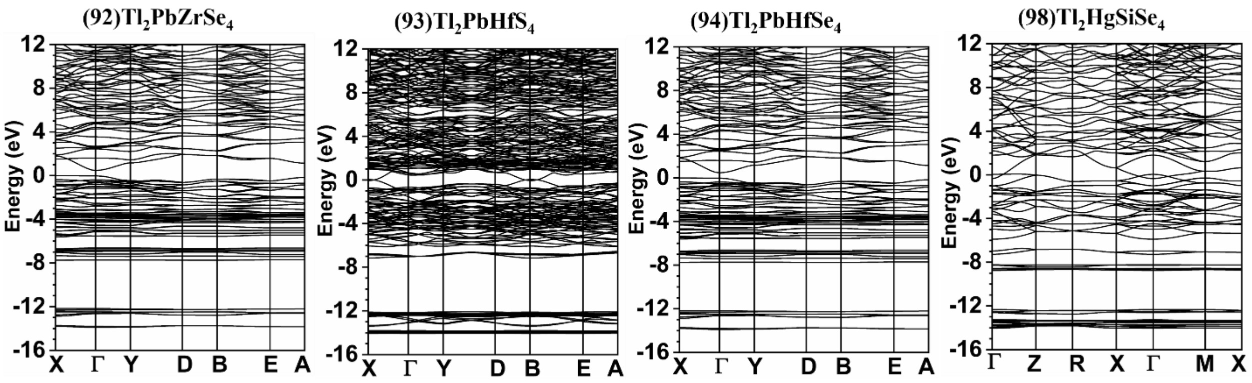


**Fig.S1. Band structure for chalcogenide crystals.**

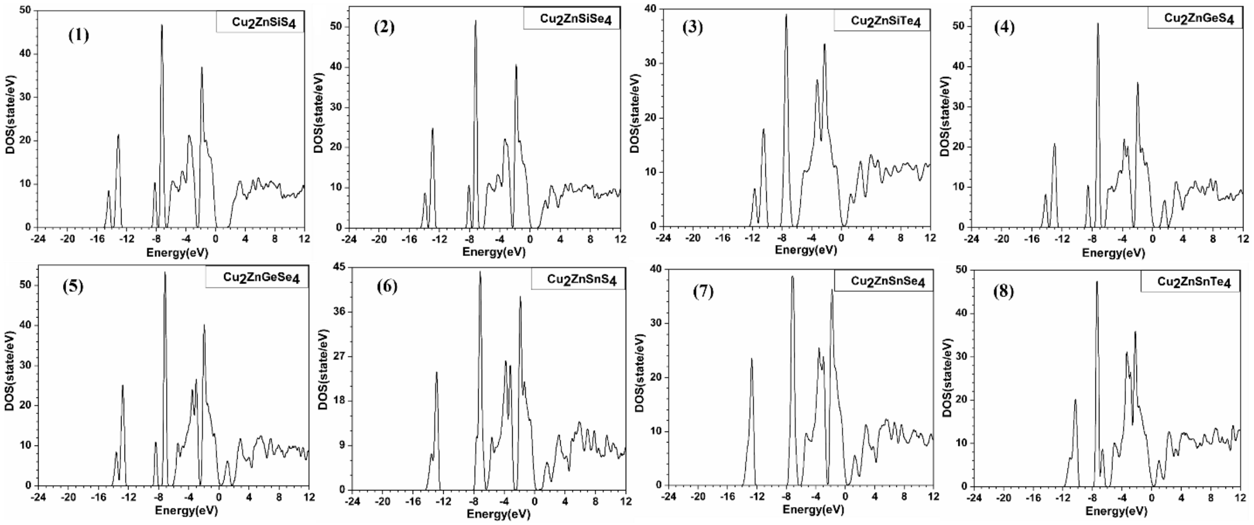

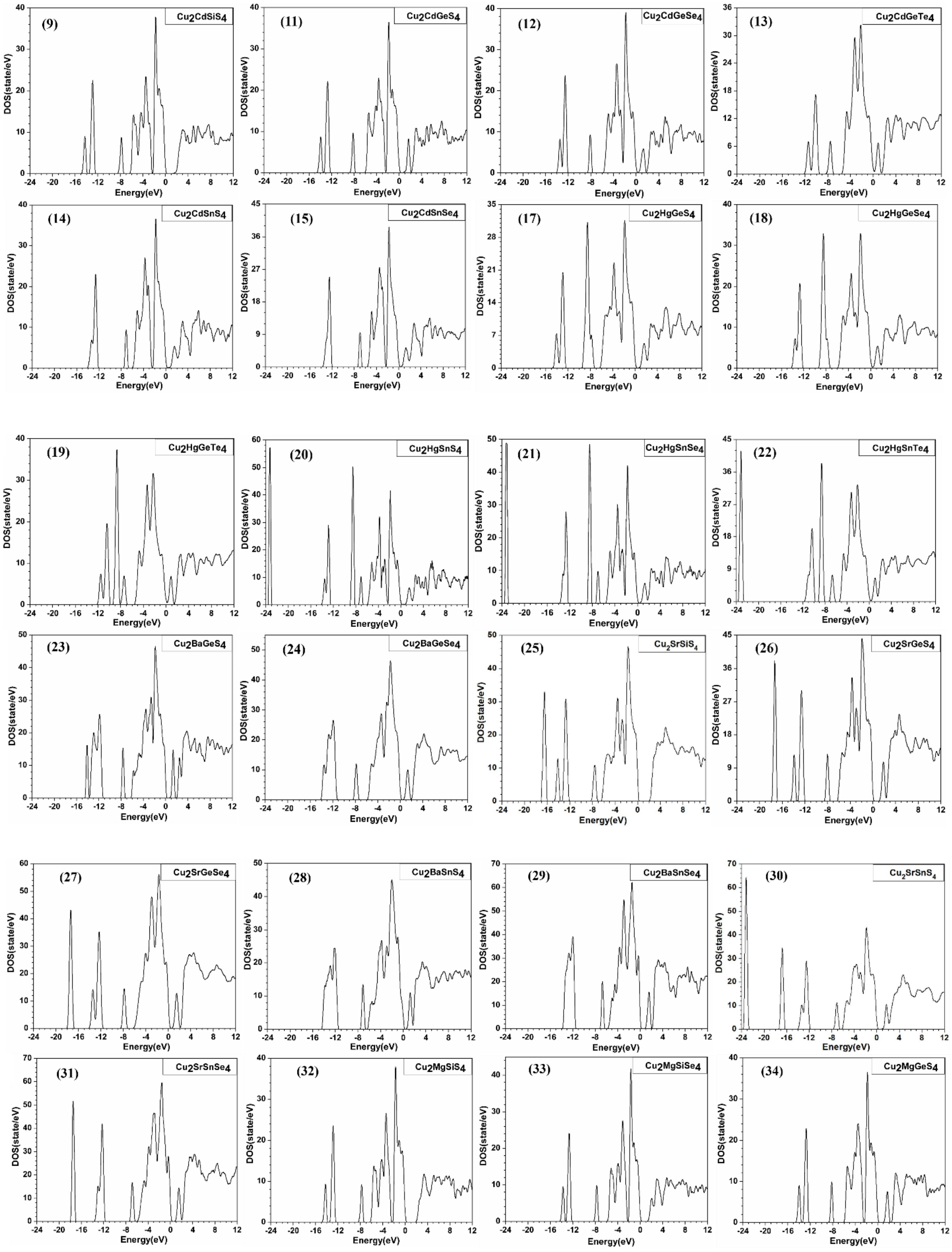
(9) Cu2CdSiS4
(11) Cu2CdGeS4
(12) Cu2CdGeSe4
(13) Cu2CdGeTe4
(14) Cu2CdSnS4
(15) Cu2CdSnSe4
(17) Cu2HgGeS4
(18) Cu2HgGeSe4
(19) Cu2HgGeTe4
(20) Cu2HgSnS4
(21) Cu2HgSnSe4
(22) Cu2HgSnTe4
(23) Cu2BaGeS4
(24) Cu2BaGeSe4
(25) Cu2SrSiS4
(26) Cu2SrGeS4
(27) Cu2SrGeSe4
(28) Cu2BaSnS4
(29) Cu2BaSnSe4
(30) Cu2SrSnS4
(31) Cu2SrSnSe4
(32) Cu2MgSiS4
(33) Cu2MgSiSe4
(34) Cu2MgGeS4
DOS(state/eV)
Energy(eV)

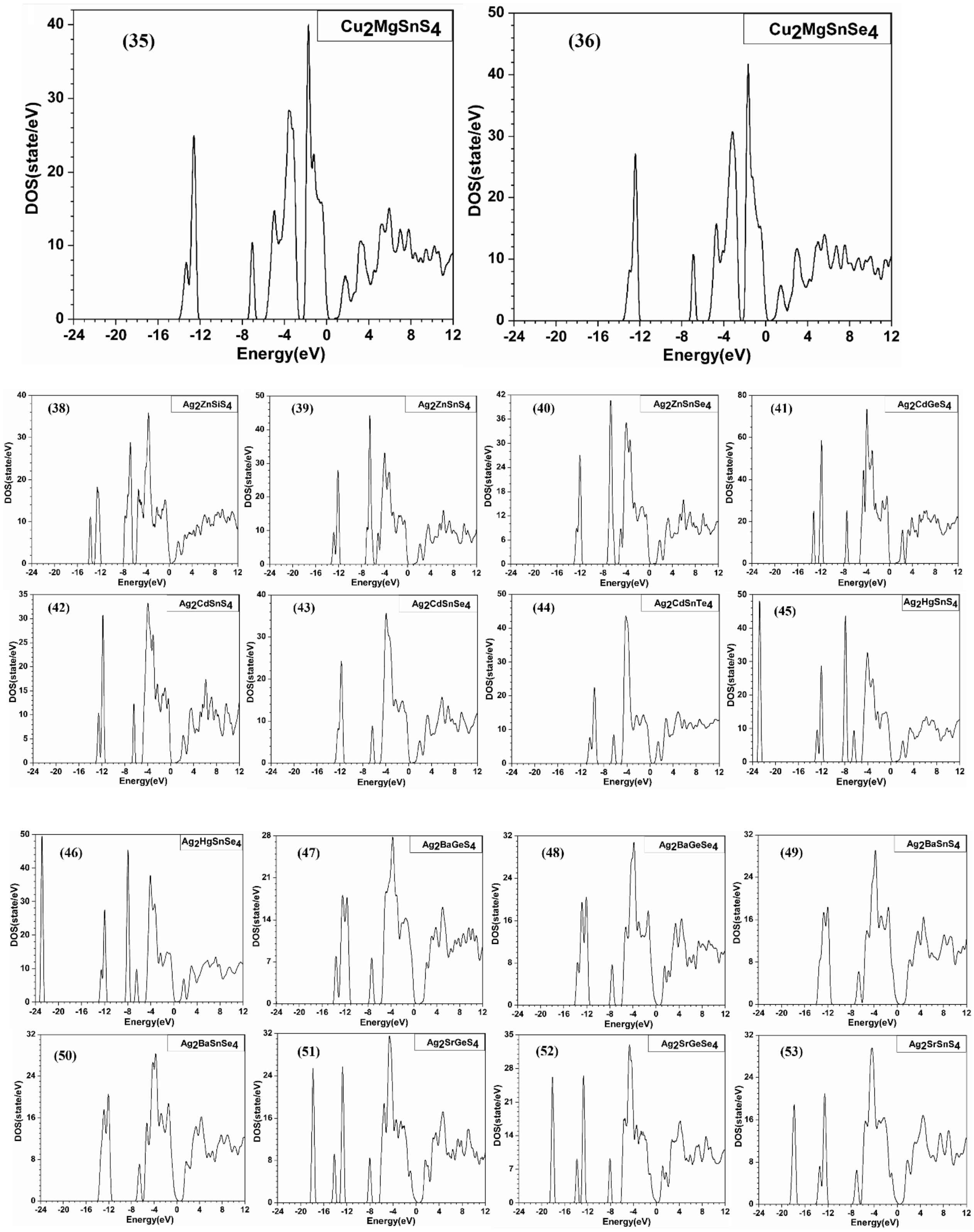
(35)
Cu2MgSnS4
(36)
Cu2MgSnSe4
(38)
Ag2ZnSiS4
(39)
Ag2ZnSnS4
(40)
Ag2ZnSnSe4
(41)
Ag2CdGeS4
(42)
Ag2CdSnS4
(43)
Ag2CdSnSe4
(44)
Ag2CdSnTe4
(45)
Ag2HgSnS4
(46)
Ag2HgSnSe4
(47)
Ag2BaGeS4
(48)
Ag2BaGeSe4
(49)
Ag2BaSnS4
(50)
Ag2BaSnSe4
(51)
Ag2SrGeS4
(52)
Ag2SrGeSe4
(53)
Ag2SrSnS4
DOS(state/eV)
Energy(eV)

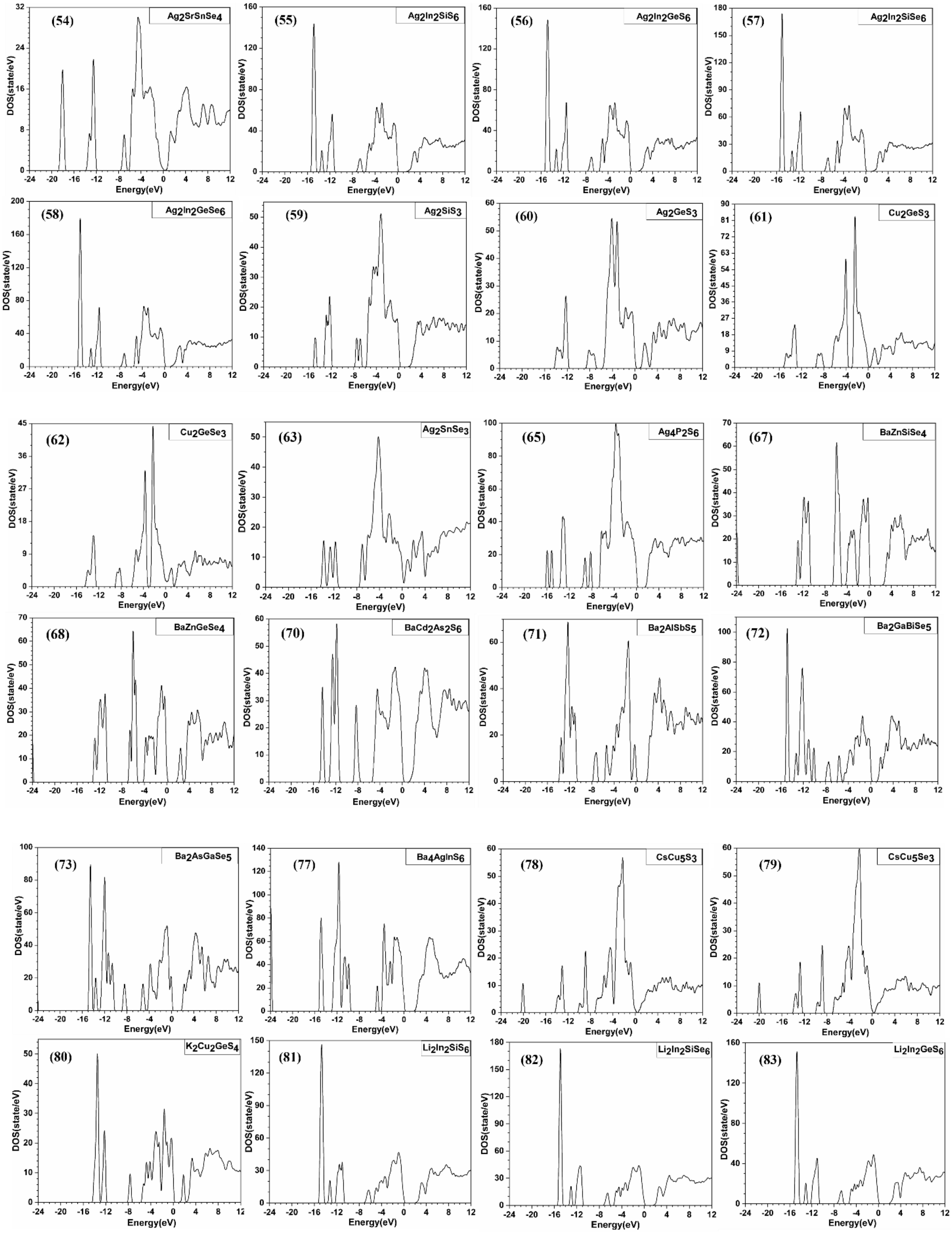
(54) Ag2SrSnSe4
(55) Ag2In2SiS6
(56) Ag2In2GeS6
(57) Ag2In2SiSe6
(58) Ag2In2GeSe6
(59) Ag2SiS3
(60) Ag2GeS3
(61) Cu2GeS3
(62) Cu2GeSe3
(63) Ag2SnSe3
(65) Ag4P2S6
(67) BaZnSiSe4
(68) BaZnGeSe4
(70) BaCd2As2S6
(71) Ba2AlSbS5
(72) Ba2GaBiSe5
(73) Ba2AsGaSe5
(77) Ba4AgInS6
(78) CsCu5S3
(79) CsCu5Se3
(80) K2Cu2GeS4
(81) Li2In2SiS6
(82) Li2In2SiSe6
(83) Li2In2GeS6
DOS(state/eV)
Energy(eV)

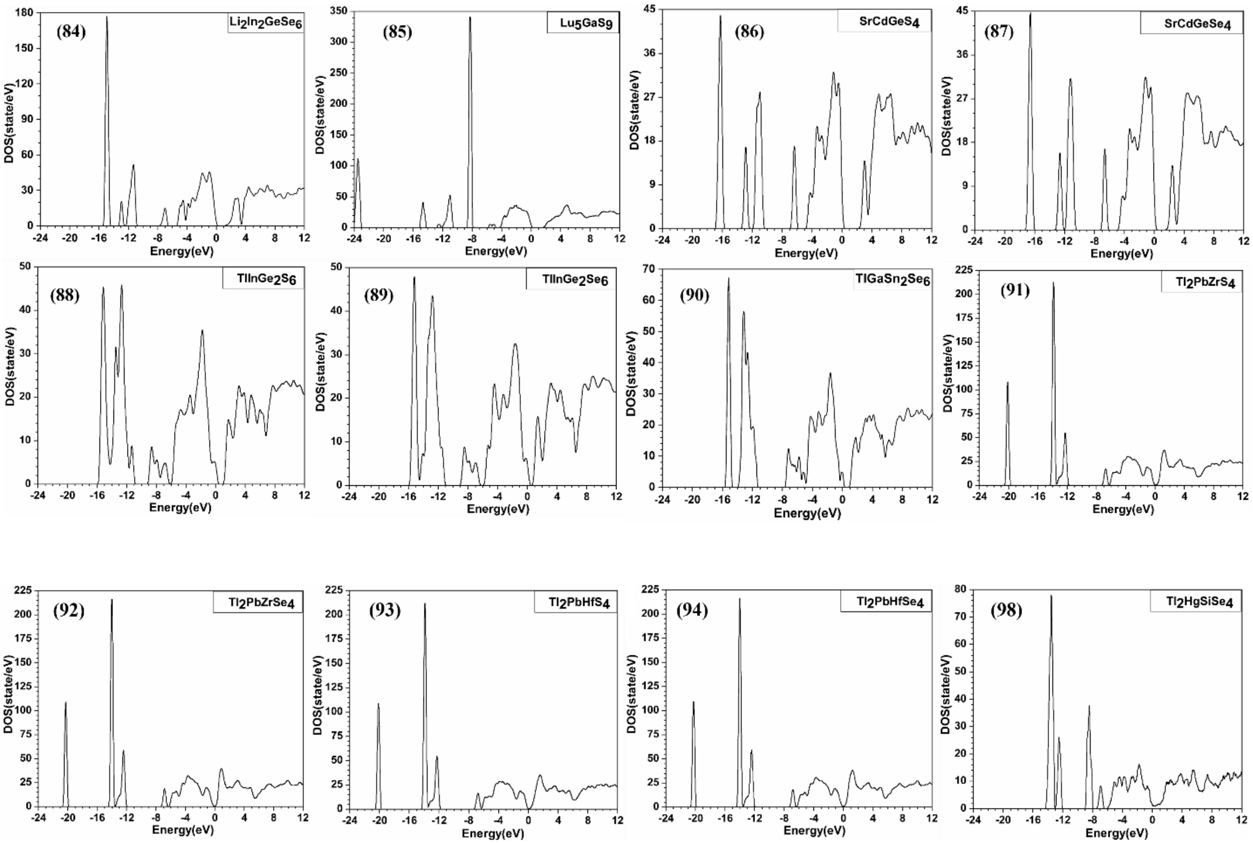


**Fig. S2. TDOS for the chalcogenide crystals.**

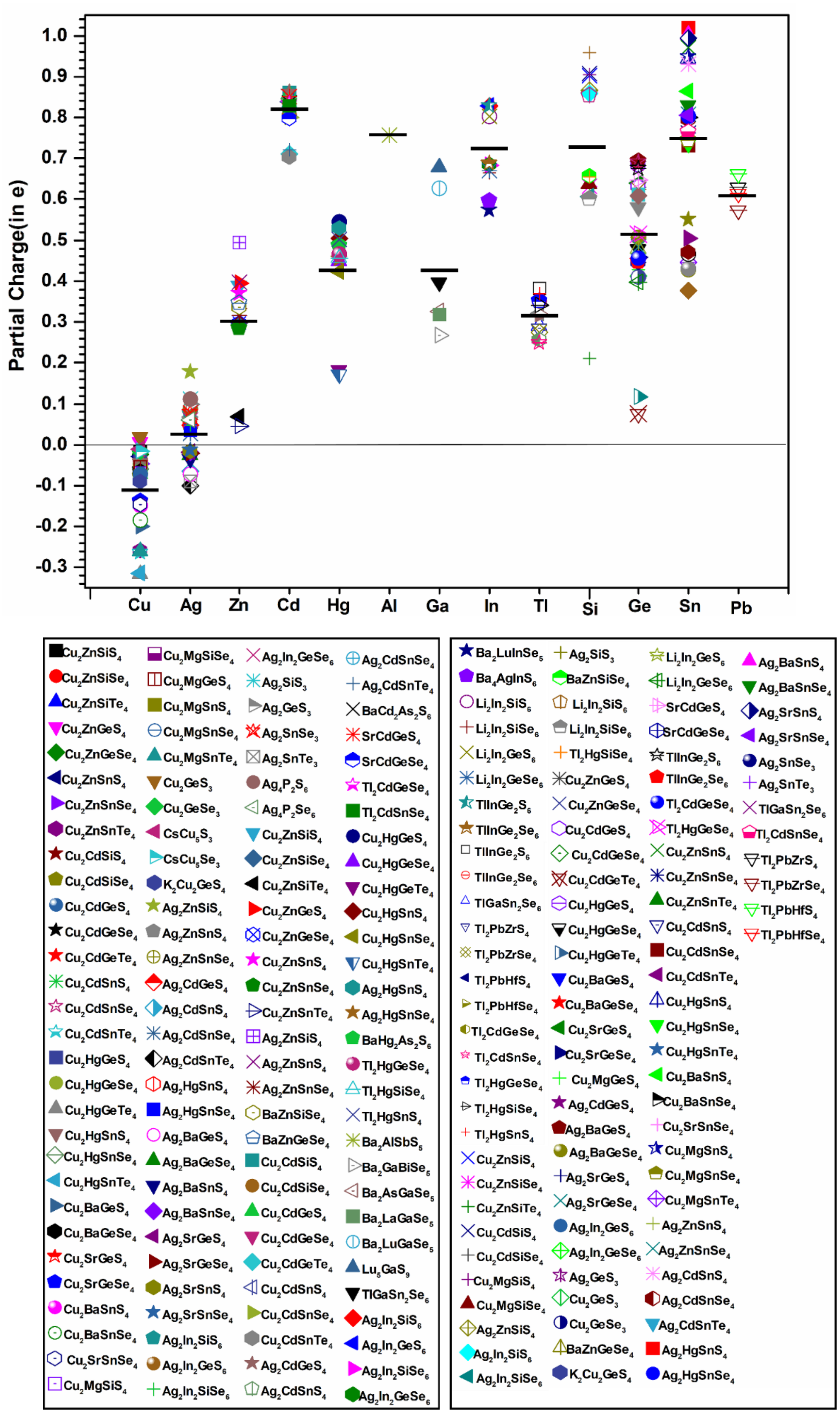


**Fig. S3. Distribution of partial charge of the elements from Cu to Pb.**

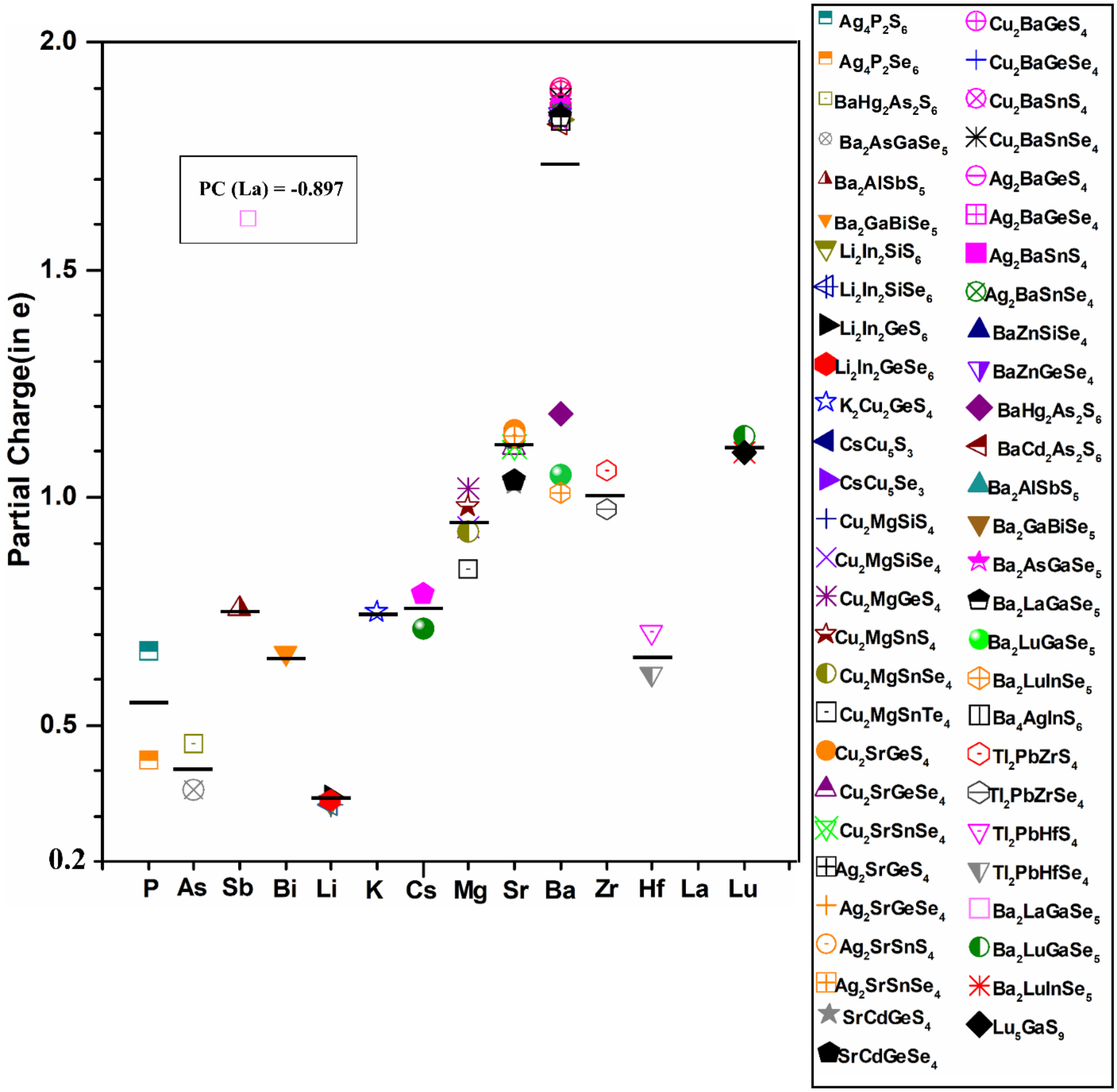


**Fig. S4. Distribution of partial charge of the elements from P to Lu.**

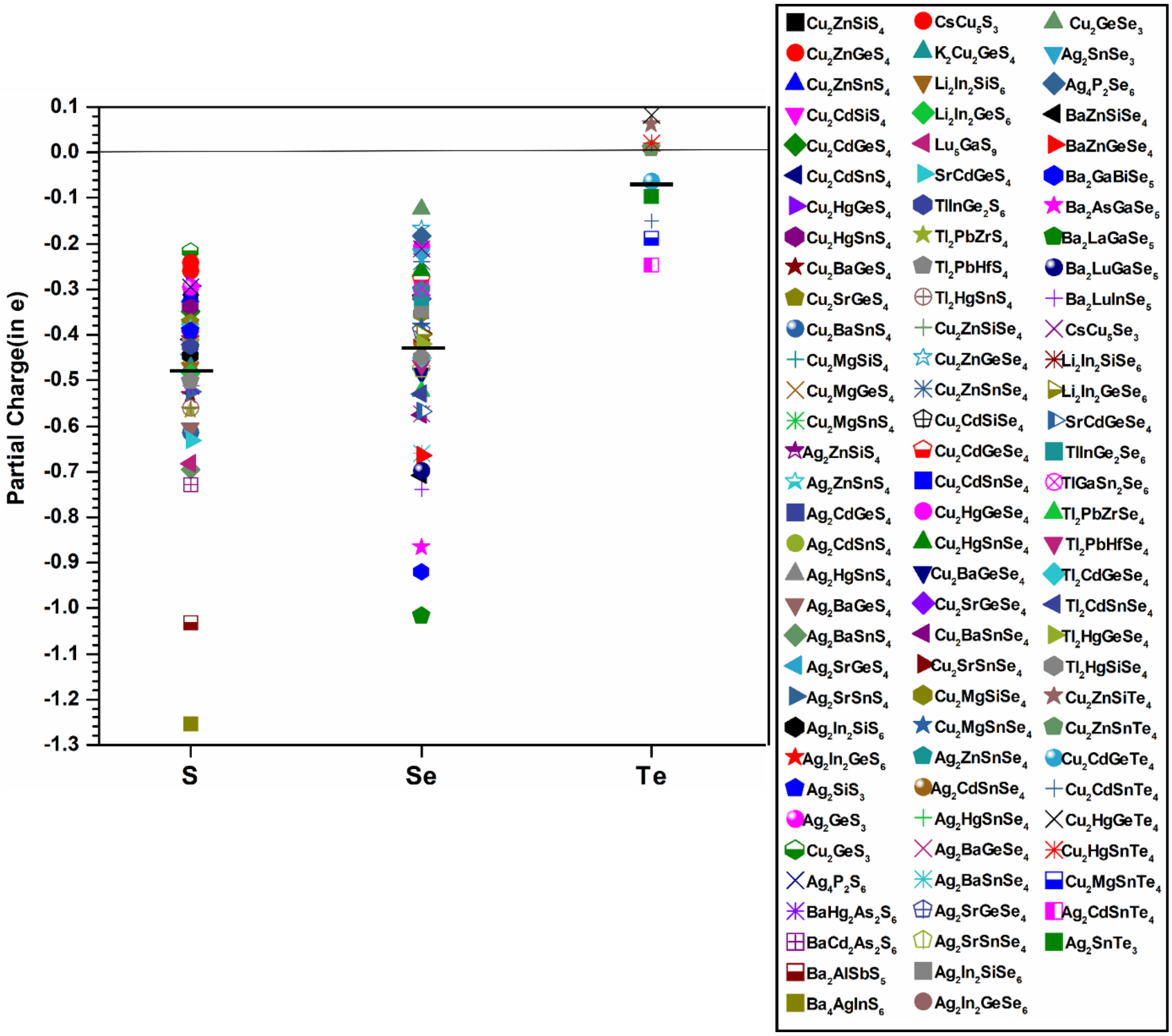


**Fig. S5. Distribution of partial charge of the chalcogene elements(S, Se, Te).**

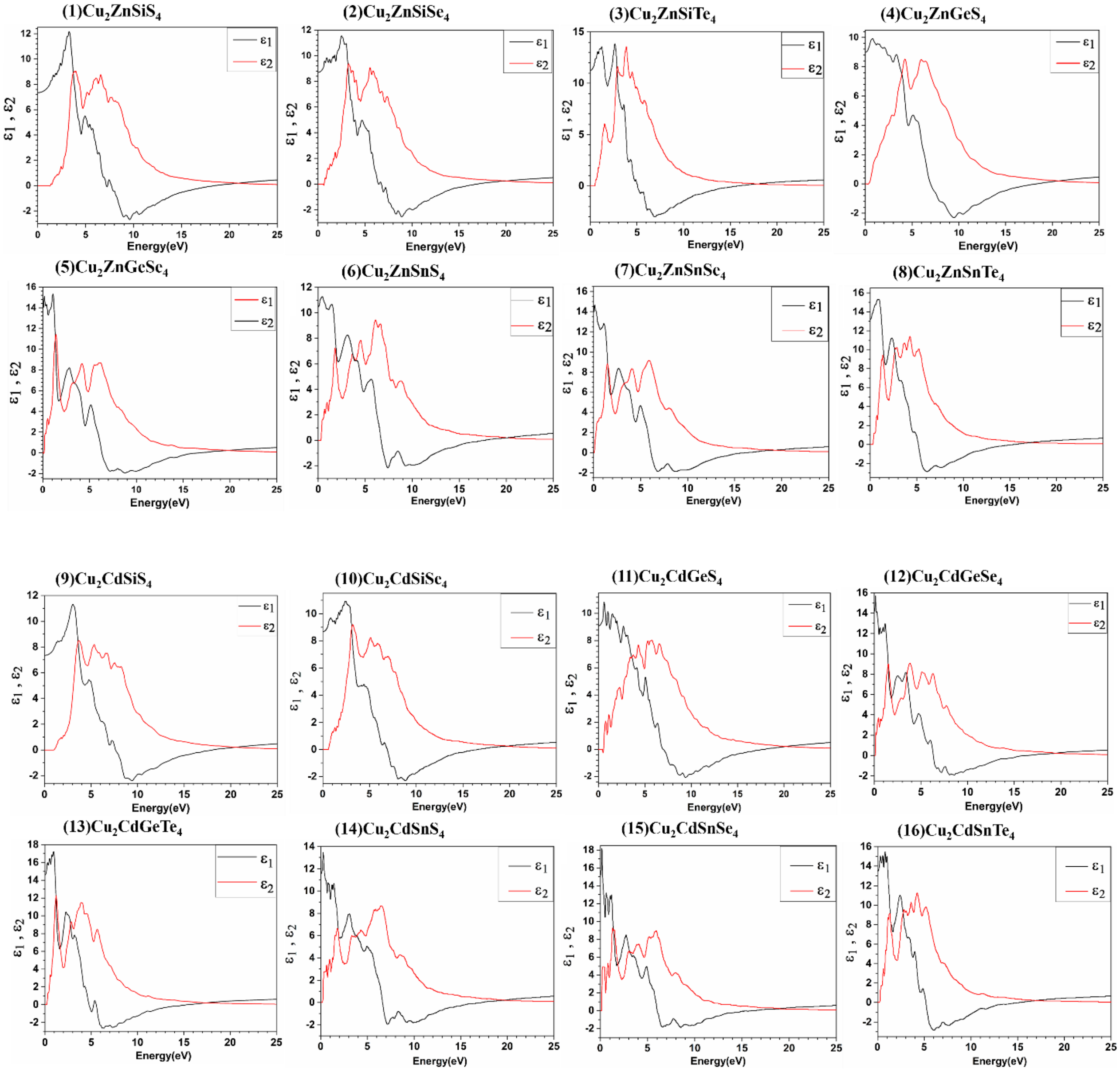

(1)$Cu_2ZnSiS_4$
(2)$Cu_2ZnSiSe_4$
(3)$Cu_2ZnSiTe_4$
(4)$Cu_2ZnGeS_4$
(5)$Cu_2ZnGeSe_4$
(6)$Cu_2ZnSnS_4$
(7)$Cu_2ZnSnSe_4$
(8)$Cu_2ZnSnTe_4$
(9)$Cu_2CdSiS_4$
(10)$Cu_2CdSiSe_4$
(11)$Cu_2CdGeS_4$
(12)$Cu_2CdGeSe_4$
(13)$Cu_2CdGeTe_4$
(14)$Cu_2CdSnS_4$
(15)$Cu_2CdSnSe_4$
(16)$Cu_2CdSnTe_4$
$\varepsilon_1$ , $\varepsilon_2$
Energy(eV)

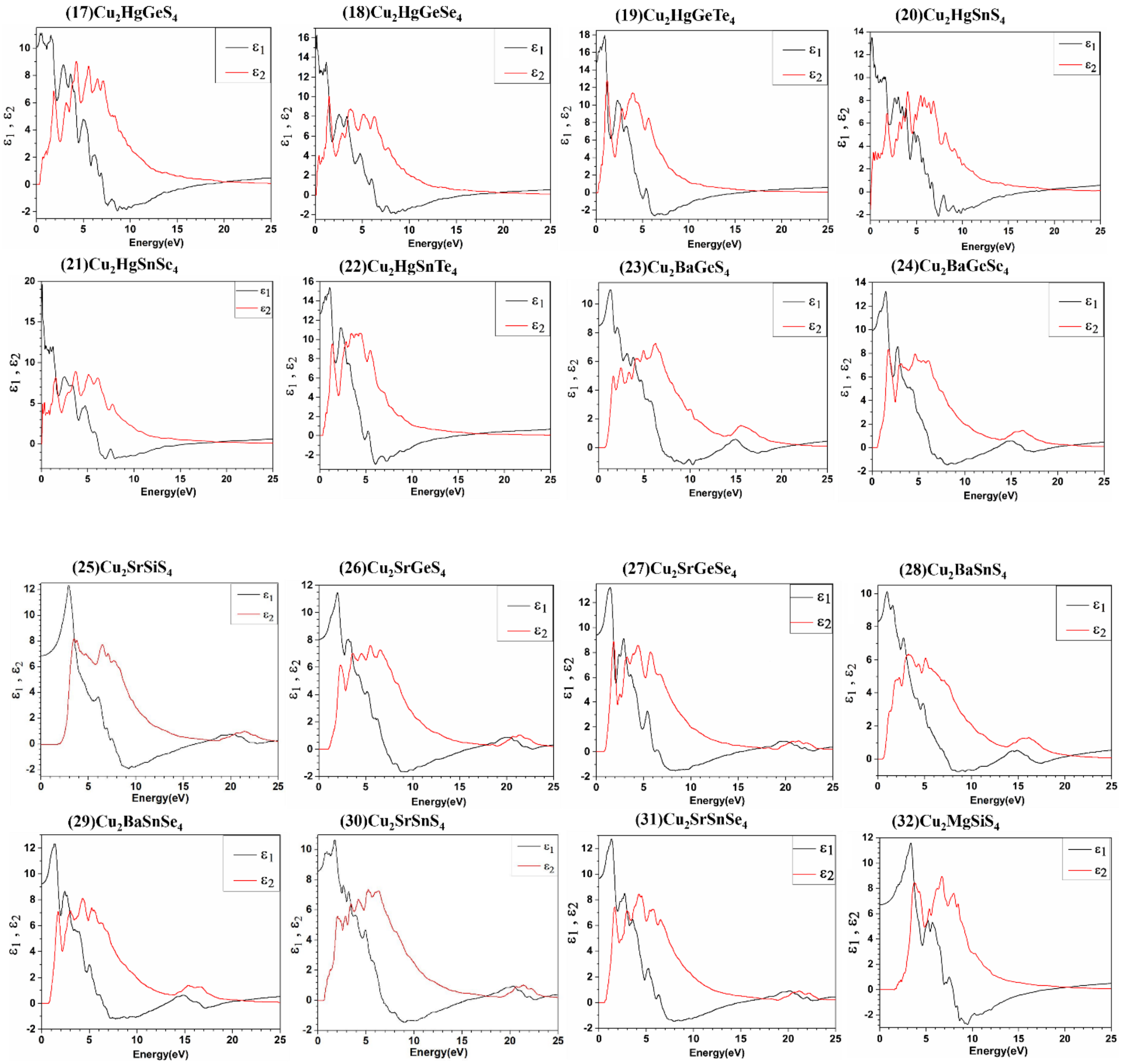
(17)$Cu_2HgGeS_4$
(18)$Cu_2HgGeSe_4$
(19)$Cu_2HgGeTe_4$
(20)$Cu_2HgSnS_4$
(21)$Cu_2HgSnSe_4$
(22)$Cu_2HgSnTe_4$
(23)$Cu_2BaGeS_4$
(24)$Cu_2BaGeSe_4$
(25)$Cu_2SrSiS_4$
(26)$Cu_2SrGeS_4$
(27)$Cu_2SrGeSe_4$
(28)$Cu_2BaSnS_4$
(29)$Cu_2BaSnSe_4$
(30)$Cu_2SrSnS_4$
(31)$Cu_2SrSnSe_4$
(32)$Cu_2MgSiS_4$
$\varepsilon_1$ , $\varepsilon_2$
Energy(eV)

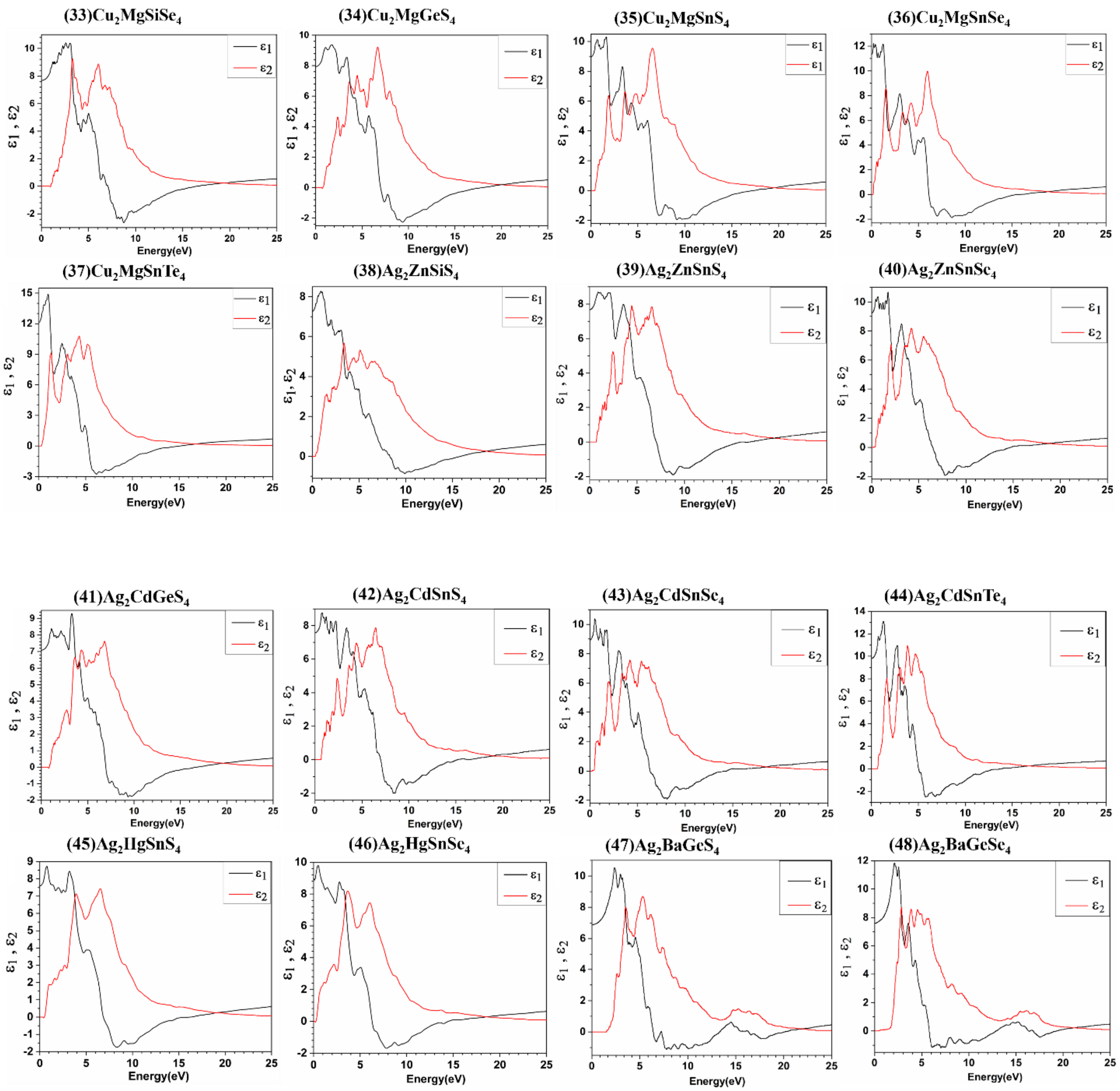
(33)$Cu_2MgSiSe_4$
(34)$Cu_2MgGeS_4$
(35)$Cu_2MgSnS_4$
(36)$Cu_2MgSnSe_4$
(37)$Cu_2MgSnTe_4$
(38)$Ag_2ZnSiS_4$
(39)$Ag_2ZnSnS_4$
(40)$Ag_2ZnSnSe_4$
(41)$Ag_2CdGeS_4$
(42)$Ag_2CdSnS_4$
(43)$Ag_2CdSnSe_4$
(44)$Ag_2CdSnTe_4$
(45)$Ag_2HgSnS_4$
(46)$Ag_2HgSnSe_4$
(47)$Ag_2BaGeS_4$
(48)$Ag_2BaGeSe_4$
$\varepsilon_1$
$\varepsilon_2$
$\varepsilon_1$ , $\varepsilon_2$
Energy(eV)

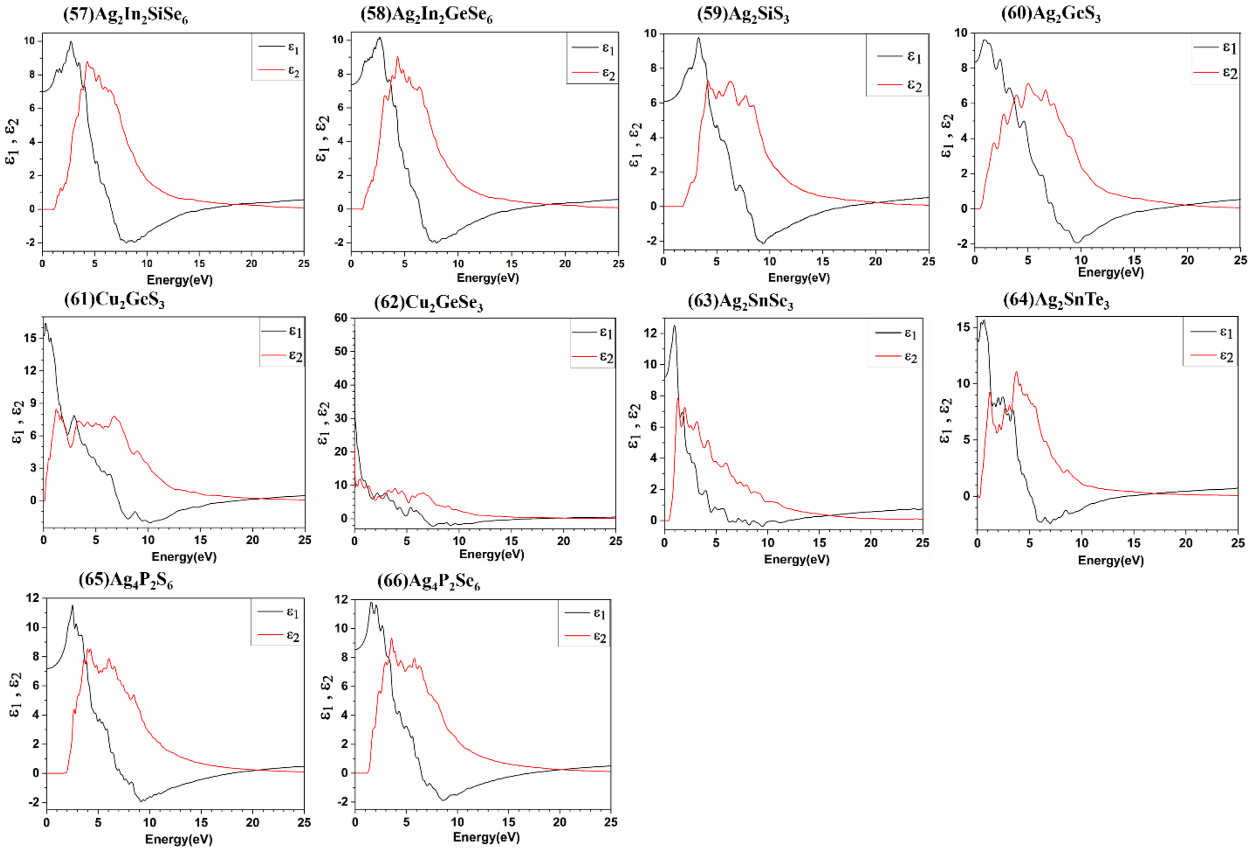
(57)$Ag_2In_2SiSe_6$
(58)$Ag_2In_2GeSe_6$
(59)$Ag_2SiS_3$
(60)$Ag_2GeS_3$
(61)$Cu_2GeS_3$
(62)$Cu_2GeSe_3$
(63)$Ag_2SnSe_3$
(64)$Ag_2SnTe_3$
(65)$Ag_4P_2S_6$
(66)$Ag_4P_2Se_6$
$\varepsilon_1$ , $\varepsilon_2$
$\varepsilon_1$
$\varepsilon_2$
Energy(eV)

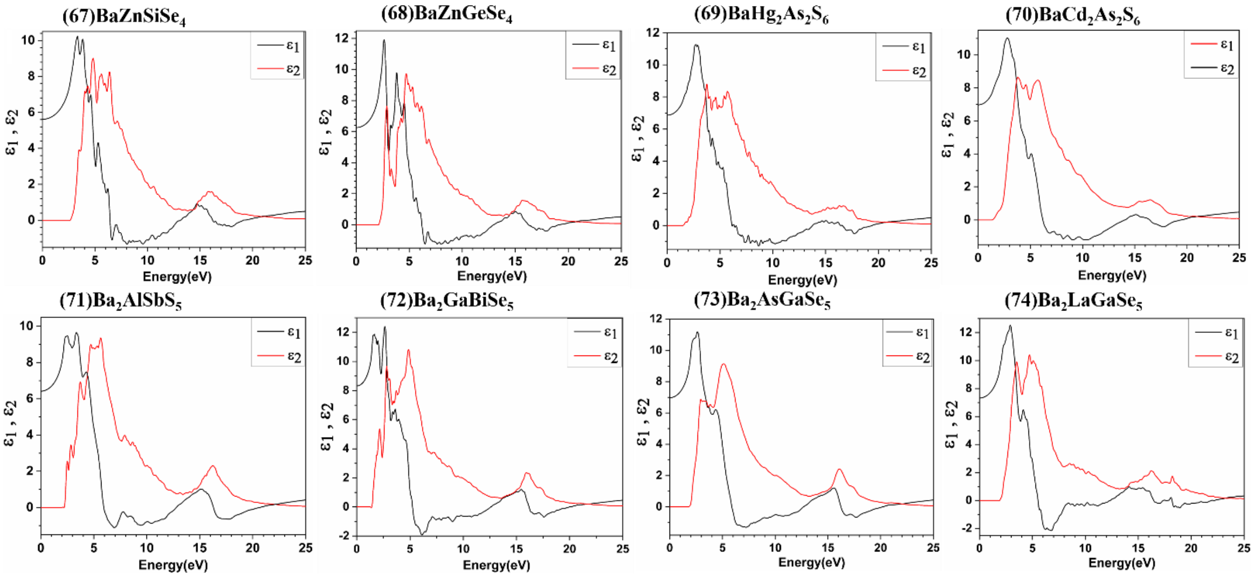
(67)$BaZnSiSe_4$
(68)$BaZnGeSe_4$
(69)$BaHg_2As_2S_6$
(70)$BaCd_2As_2S_6$
(71)$Ba_2AlSbS_5$
(72)$Ba_2GaBiSe_5$
(73)$Ba_2AsGaSe_5$
(74)$Ba_2LaGaSe_5$
$\varepsilon_1$ , $\varepsilon_2$
$\varepsilon_1$
$\varepsilon_2$
Energy(eV)

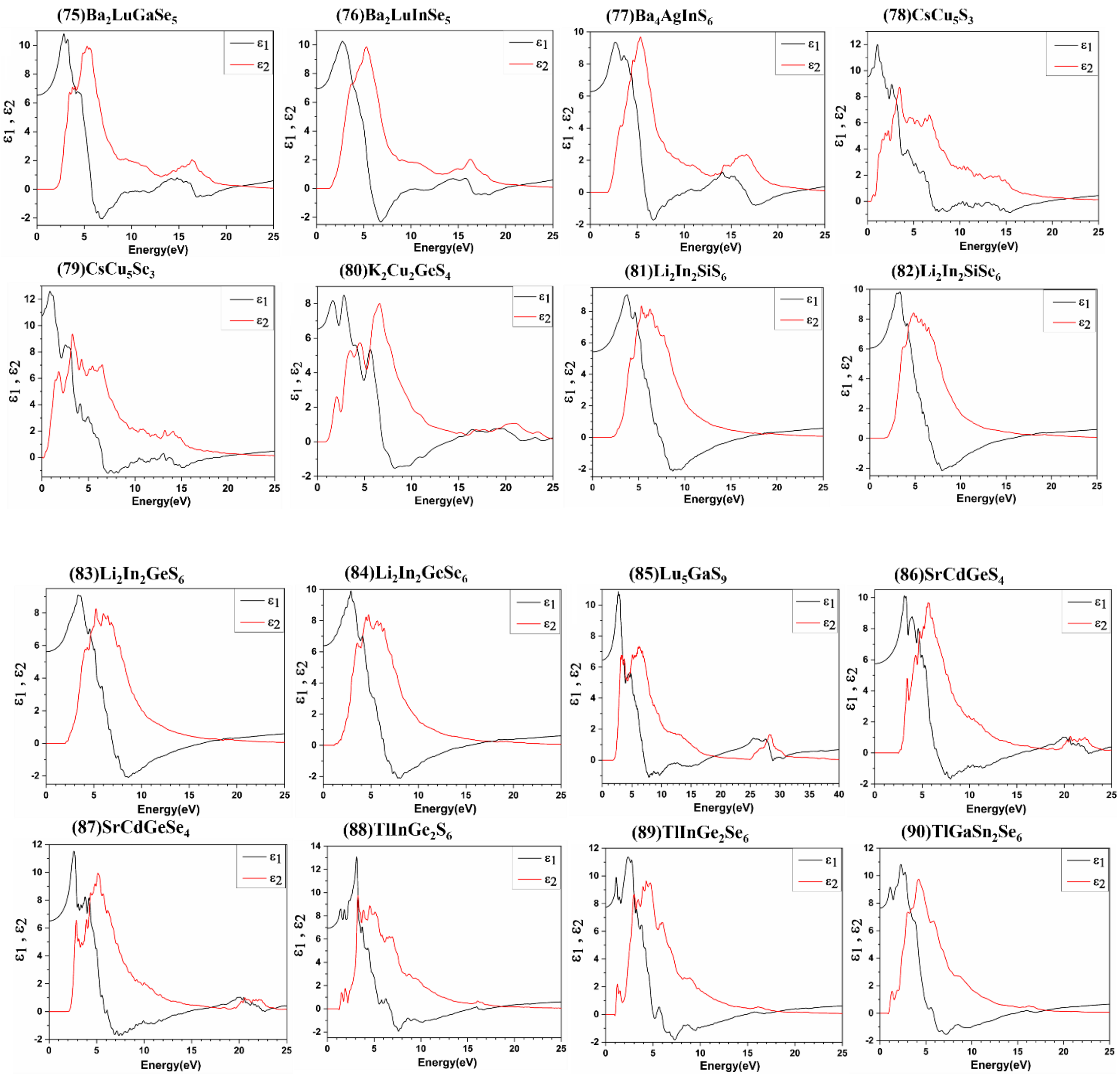
(75)Ba2LuGaSe5
(76)Ba2LuInSe5
(77)Ba4AgInS6
(78)CsCu5S3
(79)CsCu5Se3
(80)K2Cu2GeS4
(81)Li2In2SiS6
(82)Li2In2SiSe6
(83)Li2In2GeS6
(84)Li2In2GeSe6
(85)Lu5GaS9
(86)SrCdGeS4
(87)SrCdGeSe4
(88)TlInGe2S6
(89)TlInGe2Se6
(90)TlGaSn2Se6
ε1
ε2
ε1 , ε2
Energy(eV)

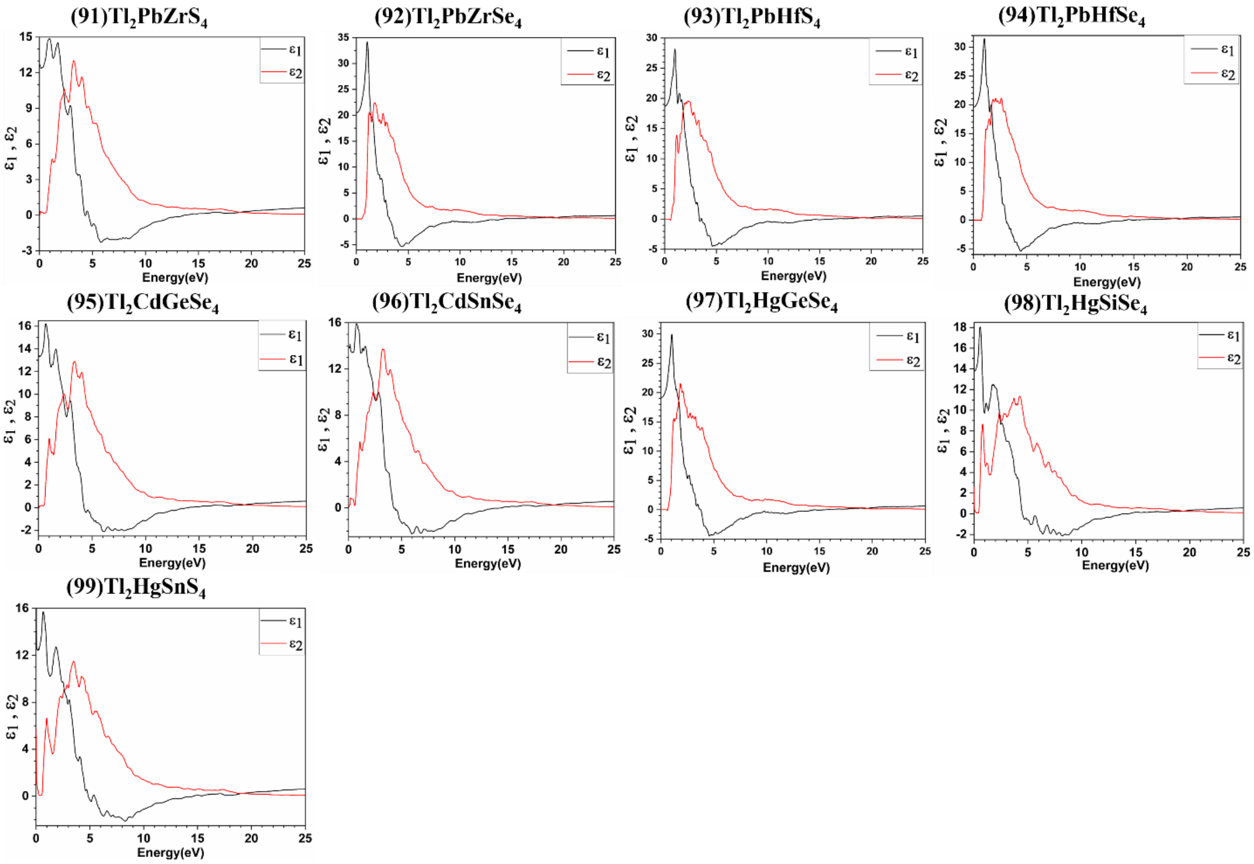


**Fig. S6. Optical dielectric function for the chalcogenide crystals. The black curve for the real part ($\varepsilon_1$), the red curve for the imaginary part ($\varepsilon_2$).**

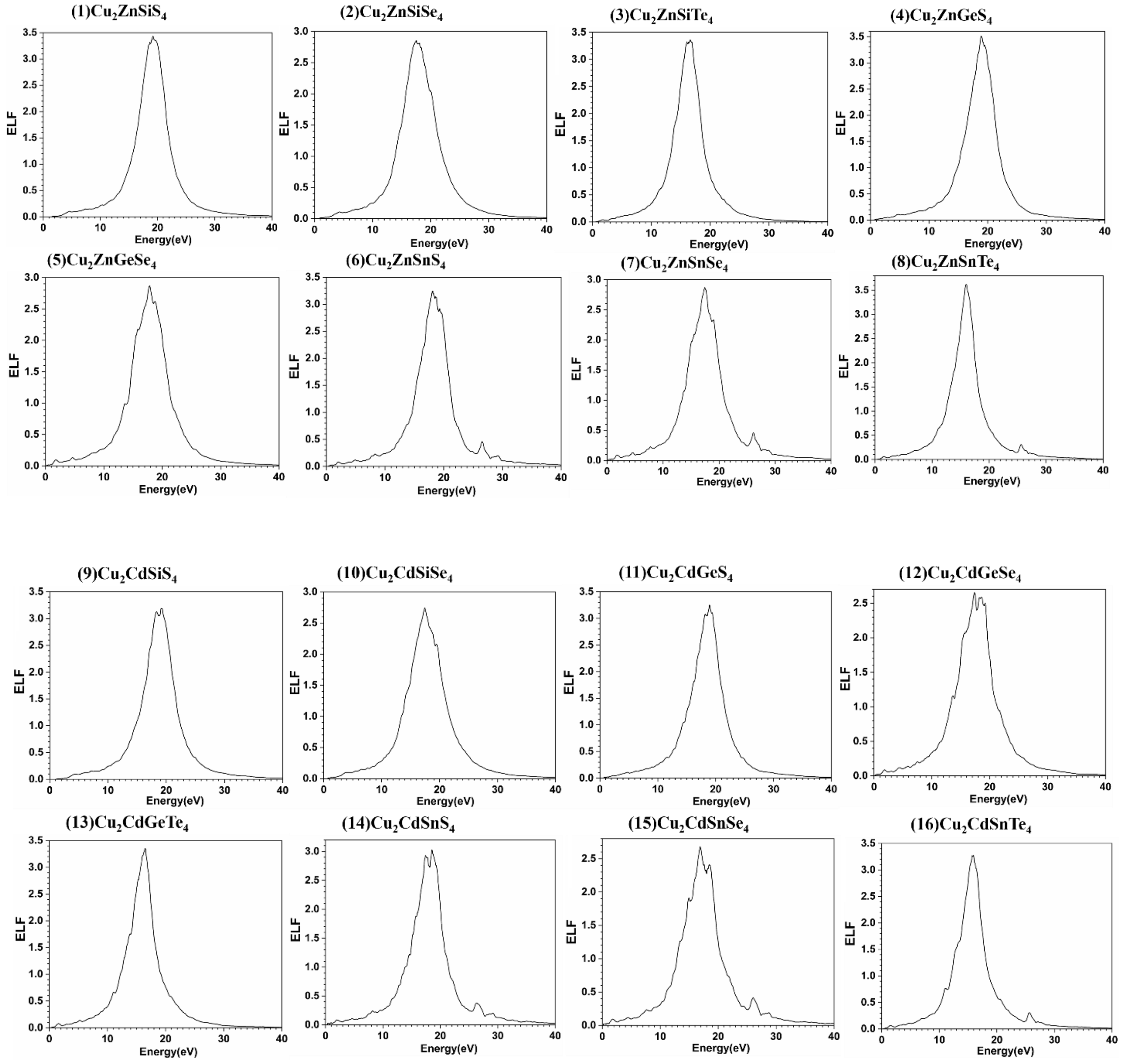
(1)Cu2ZnSiS4
(2)Cu2ZnSiSe4
(3)Cu2ZnSiTe4
(4)Cu2ZnGeS4
(5)Cu2ZnGeSe4
(6)Cu2ZnSnS4
(7)Cu2ZnSnSe4
(8)Cu2ZnSnTe4
(9)Cu2CdSiS4
(10)Cu2CdSiSe4
(11)Cu2CdGeS4
(12)Cu2CdGeSe4
(13)Cu2CdGeTe4
(14)Cu2CdSnS4
(15)Cu2CdSnSe4
(16)Cu2CdSnTe4
ELF
Energy(eV)

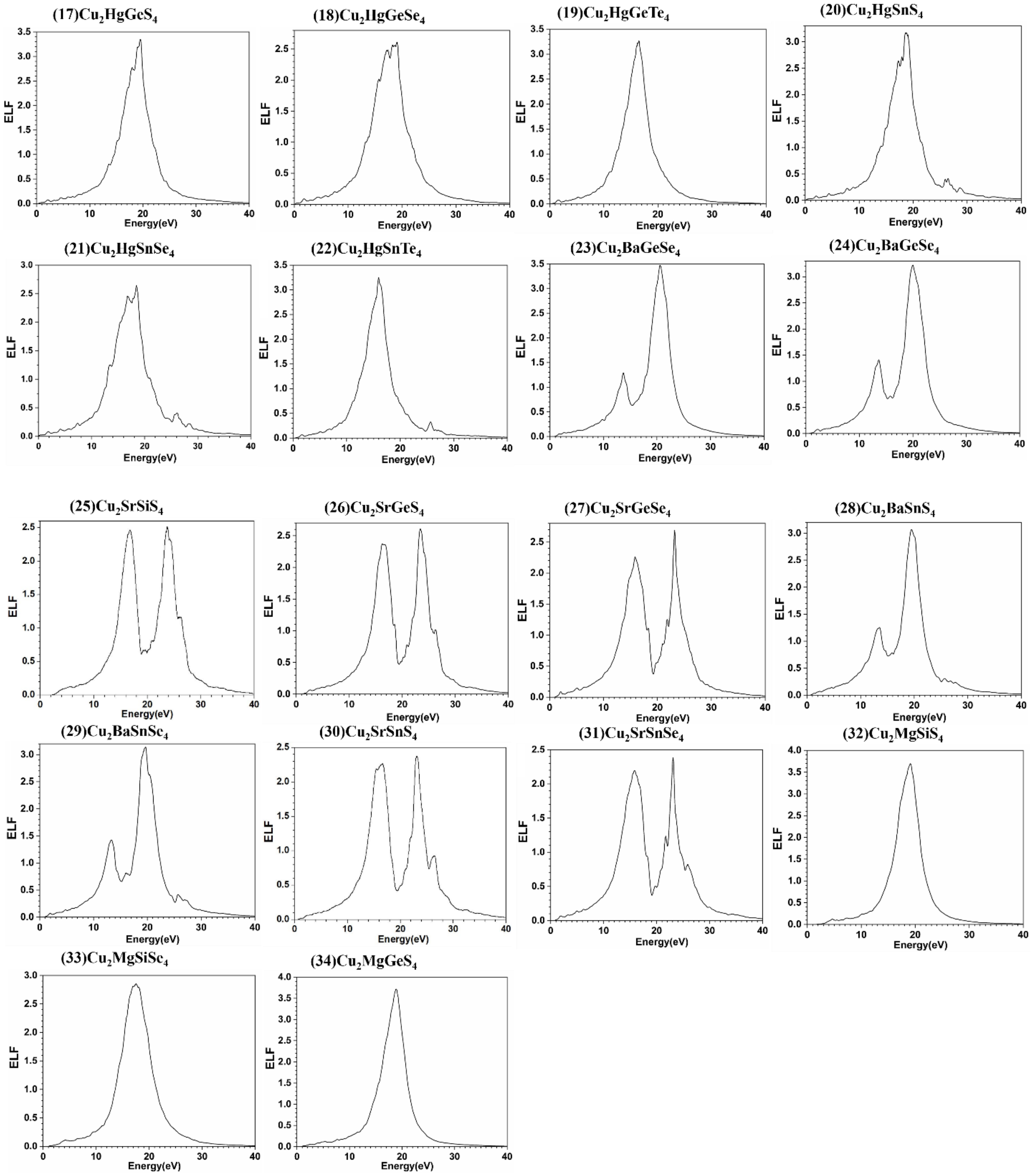
(17)Cu2HgGeS4
(18)Cu2HgGeSe4
(19)Cu2HgGeTe4
(20)Cu2HgSnS4
(21)Cu2HgSnSe4
(22)Cu2HgSnTe4
(23)Cu2BaGeSe4
(24)Cu2BaGeSe4
(25)Cu2SrSiS4
(26)Cu2SrGeS4
(27)Cu2SrGeSe4
(28)Cu2BaSnS4
(29)Cu2BaSnSe4
(30)Cu2SrSnS4
(31)Cu2SrSnSe4
(32)Cu2MgSiS4
(33)Cu2MgSiSe4
(34)Cu2MgGeS4
ELF
Energy(eV)

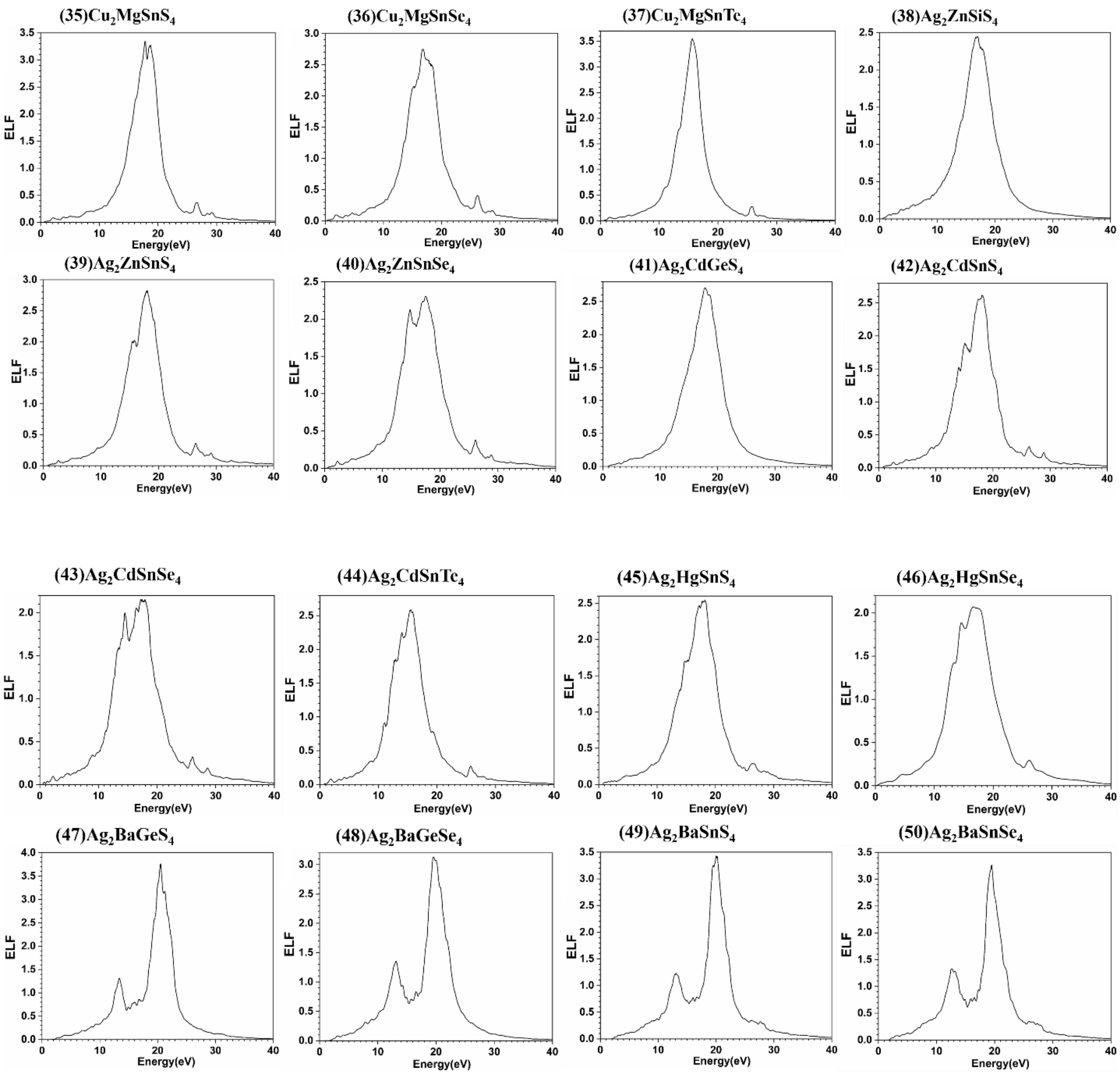
(35)$Cu_2MgSnS_4$
(36)$Cu_2MgSnSe_4$
(37)$Cu_2MgSnTe_4$
(38)$Ag_2ZnSiS_4$
(39)$Ag_2ZnSnS_4$
(40)$Ag_2ZnSnSe_4$
(41)$Ag_2CdGeS_4$
(42)$Ag_2CdSnS_4$
(43)$Ag_2CdSnSe_4$
(44)$Ag_2CdSnTe_4$
(45)$Ag_2HgSnS_4$
(46)$Ag_2HgSnSe_4$
(47)$Ag_2BaGeS_4$
(48)$Ag_2BaGeSe_4$
(49)$Ag_2BaSnS_4$
(50)$Ag_2BaSnSe_4$
ELF
Energy(eV)

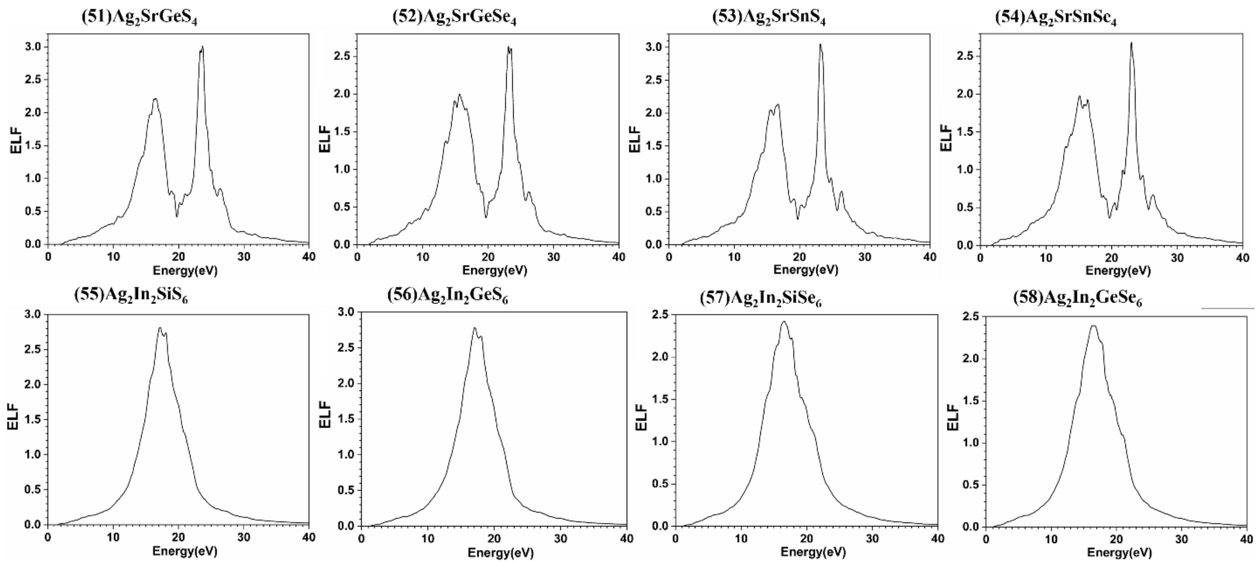
(51)$Ag_2SrGeS_4$
(52)$Ag_2SrGeSe_4$
(53)$Ag_2SrSnS_4$
(54)$Ag_2SrSnSe_4$
(55)$Ag_2In_2SiS_6$
(56)$Ag_2In_2GeS_6$
(57)$Ag_2In_2SiSe_6$
(58)$Ag_2In_2GeSe_6$
ELF
Energy(eV)

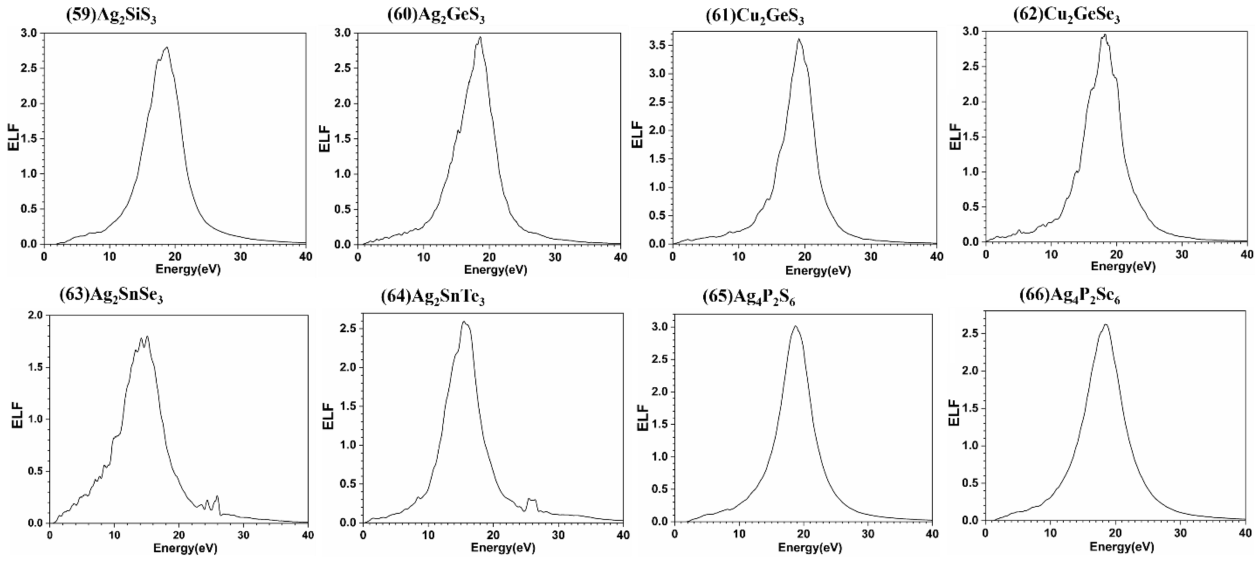
(59)$Ag_2SiS_3$
(60)$Ag_2GeS_3$
(61)$Cu_2GeS_3$
(62)$Cu_2GeSe_3$
(63)$Ag_2SnSe_3$
(64)$Ag_2SnTe_3$
(65)$Ag_4P_2S_6$
(66)$Ag_4P_2Se_6$
ELF
Energy(eV)

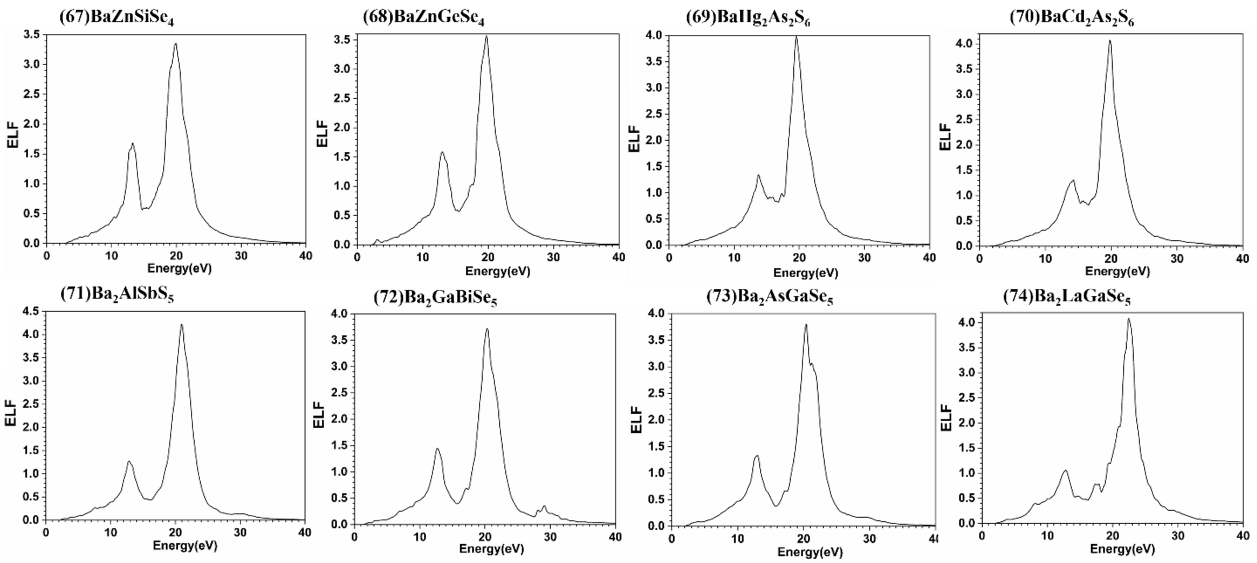
(67)BaZnSiSe4
(68)BaZnGeSe4
(69)BaHg2As2S6
(70)BaCd2As2S6
(71)Ba2AlSbS5
(72)Ba2GaBiSe5
(73)Ba2AsGaSe5
(74)Ba2LaGaSe5
ELF
Energy(eV)

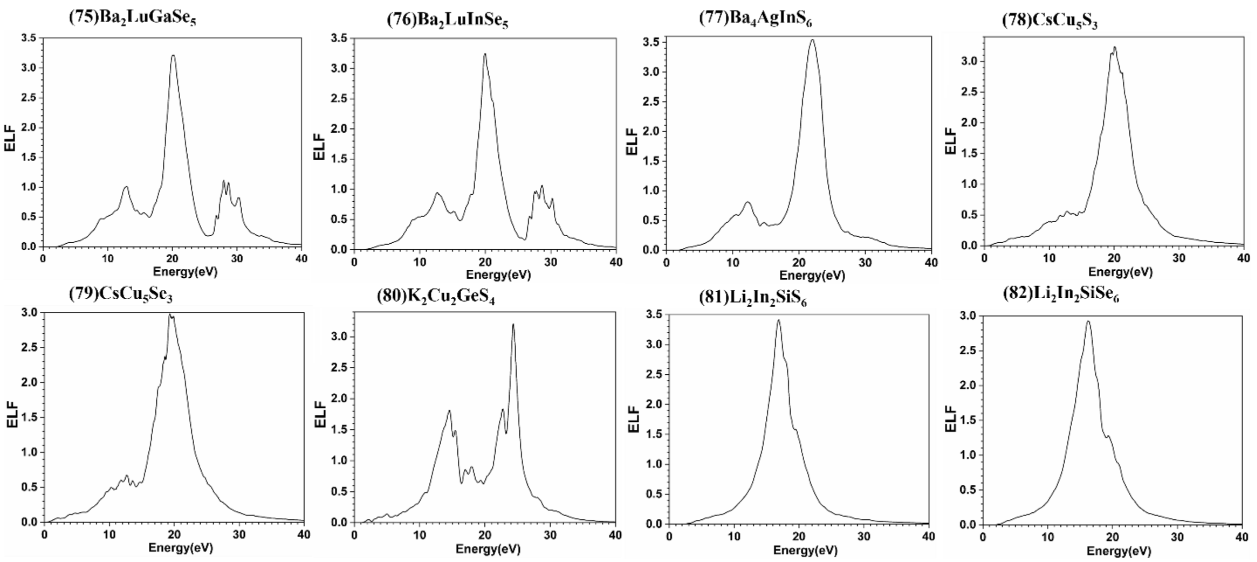
(75)Ba2LuGaSe5
(76)Ba2LuInSe5
(77)Ba4AgInS6
(78)CsCu5S3
(79)CsCu5Se3
(80)K2Cu2GeS4
(81)Li2In2SiS6
(82)Li2In2SiSe6
ELF
Energy(eV)

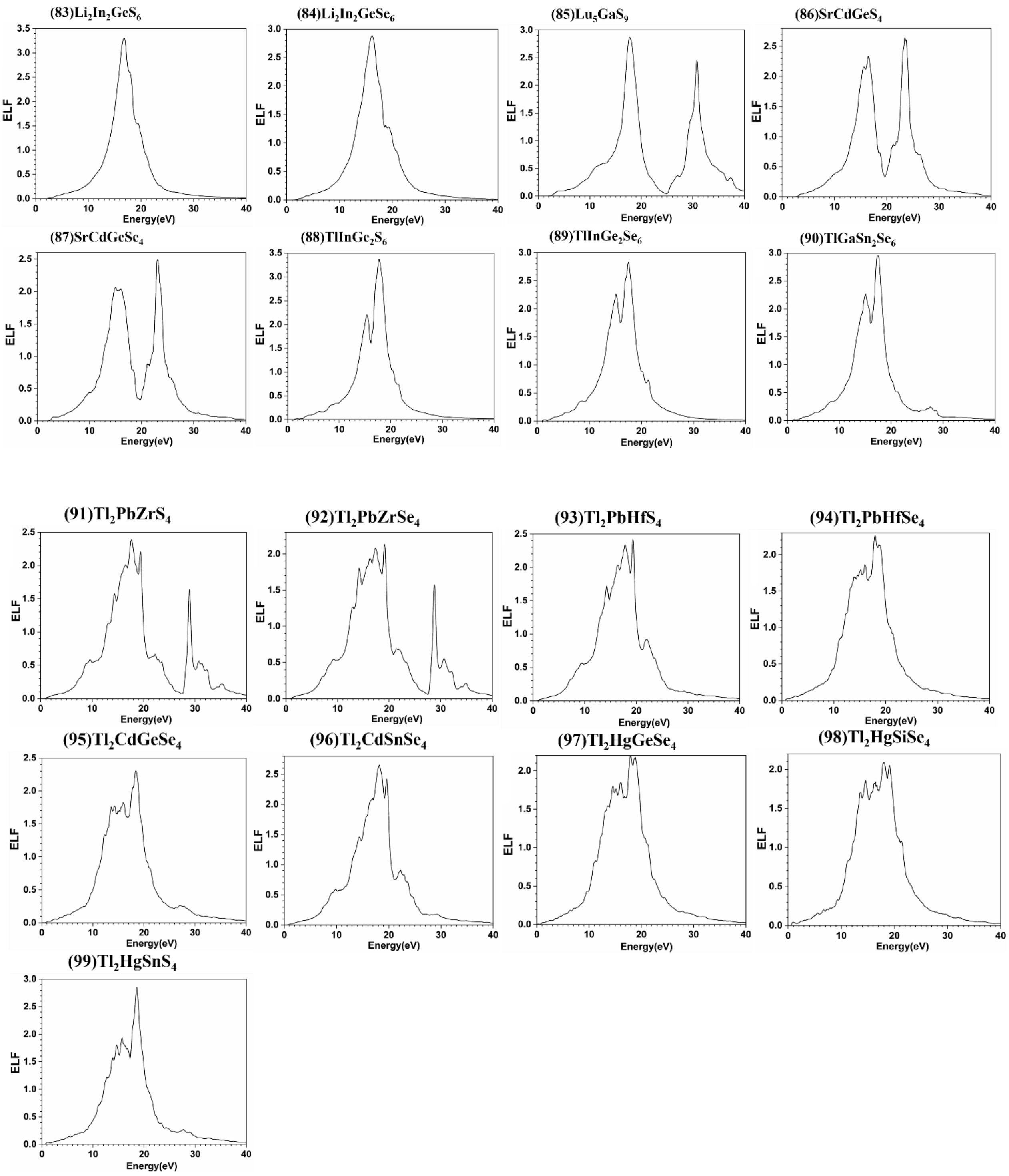


**Fig. S7. The Optical Loss function for the chalcogenide crystals**

## 5. References:


[1] W.-Y. Ching, P. Rulis, Electronic Structure Methods for Complex Materials: The orthogonalized linear combination of atomic orbitals, Oxford University Press, 2012.

[2] H. Yao, L. Ouyang, W.Y. Ching, Ab initio calculation of elastic constants of ceramic crystals, Journal of the American Ceramic Society, 90 (2007) 3194-3204.

[3] A. Reuss, Berechnung der fließgrenze von mischkristallen auf grund der plastizitätsbedingung für einkristalle, ZAMM-Journal of Applied Mathematics and Mechanics/Zeitschrift für Angewandte Mathematik und Mechanik, 9 (1929) 49-58.

[4] R. Hill, The elastic behaviour of a crystalline aggregate, Proceedings of the Physical Society. Section A, 65 (1952) 349.

[5] Y. Gao, Y. Dong, Structural, mechanical and thermal properties of Cu2ZnSiS4 with four structures from the first-principle calculations, International Journal of Modern Physics B, 33 (2019) 1950067.

[6] A. Litvinchuk, V. Dzhagan, V. Yukhymchuk, M.Y. Valakh, O. Parasyuk, L. Piskach, X. Wang, A. Jacobson, D. Zahn, Crystal structure and vibrational properties of Cu2ZnSiSe4 quaternary semiconductor, physica status solidi (b), 253 (2016) 1808-1815.

[7] S. Levcenko, A. Nateprov, V. Kravtsov, M. Guc, A. Pérez-Rodríguez, V. Izquierdo-Roca, X. Fontané, E. Arushanov, Structural study and Raman scattering analysis of Cu 2 ZnSiTe 4 bulk crystals, Optics express, 22 (2014) A1936-A1943.

[8] M. Guc, A. Litvinchuk, S. Levcenko, V. Izquierdo-Roca, X. Fontané, M.Y. Valakh, E. Arushanov, A. Pérez-Rodríguez, Optical phonons in the wurtzstannite Cu 2 ZnGeS 4 semiconductor: Polarized Raman spectroscopy and first-principle calculations, Physical Review B, 89 (2014) 205205.

[9] O. Khyzhun, V. Bekenev, V. Ocheretova, A. Fedorchuk, O. Parasyuk, Electronic structure of Cu2ZnGeSe4 single crystal: Ab initio FP-LAPW calculations and X-ray spectroscopy measurements, Physica B: Condensed Matter, 461 (2015) 75-84.

[10] L. Kong, J.X. Deng, First-Principles Study on Electronic and Optical Properties of Kesterite and Stannite Cu2ZnSnS4 Photovoltaic Absorbers, in: Materials Science Forum, Trans Tech Publ, 2015, pp. 80-88.

[11] W. Gong, T. Tabata, K. Takei, M. Morihama, T. Maeda, T. Wada, Crystallographic and optical properties of (Cu, Ag) 2ZnSnS4 and (Cu, Ag) 2ZnSnSe4 solid solutions, physica status solidi (c), 12 (2015) 700-703.

[12] N. Ullah, G. Murtaza, M. Iqbal, A. Mahmood, R. Khenata, Computational study of Cu 2 ZnSn (X 1− x Te x) 4 (X= S, Se) for optoelectronic applications, International Journal of Modern Physics B, 30 (2016) 1650137.

[13] G. Chapuis, A. Niggli, The crystal structure of thenormal tetrahedral'compound Cu2CdSiS4, Acta Crystallographica Section B: Structural Crystallography and Crystal Chemistry, 28 (1972) 1626-1628.

[14] H. Matsushita, T. Ichikawa, A. Katsui, Structural, thermodynamical and optical properties of Cu 2-II-IV-VI 4 quaternary compounds, Journal of materials science, 40 (2005) 2003-2005.

[15] E. Parthé, K. Yvon, R. Deitch, The crystal structure of Cu2CdGeS4 and other quaternary normal tetrahedral structure compounds, Acta Crystallographica Section B: Structural Crystallography and Crystal Chemistry, 25 (1969) 1164-1174.

[16] M. Brik, O. Parasyuk, G. Myronchuk, I. Kityk, Specific features of band structure and optical anisotropy of Cu2CdGeSe4 quaternary compounds, Materials Chemistry and Physics, 147 (2014) 155-161.

[17] H. Haeuseler, F. Ohrendorf, M. Himmrich, Zur Kenntnis quaternärer Telluride Cu2MM′ Te4 mit Tetraederstrukturen/Quaternary Tellurides Cu2MM′ Te4 with Tetrahedral Structures, Zeitschrift für Naturforschung B, 46 (1991) 1049-1052.

[18] S. Hussain, G. Murtaza, S.H. Khan, A. Khan, M.A. Ali, M. Faizan, A. Mahmood, R. Khenata, First principles study of structural, optoelectronic and thermoelectric properties of Cu2CdSnX4 (X= S, Se, Te) chalcogenides, Materials Research Bulletin, 79 (2016) 73-83.
[19] T.V. Vu, A. Lavrentyev, B. Gabrelian, V. Tkach, K.D. Pham, O. Marchuk, O. Parasyuk, O. Khyzhun, First-principles DFT computation and X-ray spectroscopy study of the electronic band structure and optical constants of Cu2HgGeS4, Solid State Sciences, (2020) 106287.
[20] B. Gabrelian, A. Lavrentyev, T.V. Vu, V. Tkach, O. Marchuk, K. Kalmykova, L. Ananchenko, O. Parasyuk, O. Khyzhun, Quaternary Cu2HgGeSe4 selenide: Its electronic and optical properties as elucidated from TB-mBJ band-structure calculations and XPS and XES measurements, Chemical Physics, (2020) 110821.
[21] B. Gabrelian, A. Lavrentyev, T.V. Vu, K. Kalmykova, L. Ananchenko, V. Tkach, O. Parasyuk, O. Khyzhun, Valence-band electronic structure and main optical properties of Cu2HgGeTe4: Theoretical simulation within a DFT framework and experimental XPS study, Materials Today Communications, 23 (2020) 100828.
[22] T.V. Vu, A. Lavrentyev, B. Gabrelian, H.D. Tong, V. Tkach, O. Parasyuk, O. Khyzhun, A theoretical and experimental study of the valence-band electronic structure and optical constants of quaternary copper mercury tin sulfide, Cu2HgSnS4, a potential material for optoelectronics and solar cells, Optical Materials, 96 (2019) 109296.
[23] T.V. Vu, A. Lavrentyev, B. Gabrelian, H.D. Tong, V. Tkach, O. Parasyuk, O. Khyzhun, Simulation within a DFT framework and experimental study of the valence-band electronic structure and optical properties of quaternary selenide Cu2HgSnSe4, Optik, 202 (2020) 163709.
[24] J. Navrátil, V. Kucek, T. Plecháček, E. Černošková, F. Laufek, Č. Drašar, P. Knotek, Thermoelectric Properties of Cu 2 HgSnSe 4-Cu 2 HgSnTe 4 Solid Solution, Journal of electronic materials, 43 (2014) 3719-3725.
[25] T. Zhu, W.P. Huhn, G.C. Wessler, D. Shin, B. Saparov, D.B. Mitzi, V. Blum, I2–II–IV–VI4 (I= Cu, Ag; II= Sr, Ba; IV= Ge, Sn; VI= S, Se): Chalcogenides for Thin-Film Photovoltaics, Chemistry of Materials, 29 (2017) 7868-7879.
[26] M. Tampier, D. Johrendt, Kristallstrukturen und chemische Bindung von AM2GeSe4 (A= Sr, Ba; M= Cu, Ag), Zeitschrift für anorganische und allgemeine Chemie, 627 (2001) 312-320.
[27] J.-P. Sun, G.C. McKeown Wessler, T. Wang, T. Zhu, V. Blum, D.B. Mitzi, Structural tolerance factor approach to defect-resistant I2-II-IV-X4 semiconductor design, Chemistry of Materials, 32 (2020) 1636-1649.
[28] S.P. Ramkumar, Structural, Vibrational, and Electronic Properties of Trigonal Cu2SrSnS4 Photovoltaic Absorber from First-Principles Calculations, Material Science Research India, 17 (2020) 07-12.
[29] A. Bedjaoui, A. Bouhemadou, S. Aloumi, R. Khenata, S. Bin-Omran, Y. Al-Douri, F.S. Saoud, S. Bensalem, Structural, elastic, electronic and optical properties of the novel quaternary diamond-like semiconductors Cu2MgSiS4 and Cu2MgGeS4, Solid State Sciences, 70 (2017) 21-35.
[30] B.-W. Liu, M.-J. Zhang, Z.-Y. Zhao, H.-Y. Zeng, F.-K. Zheng, G.-C. Guo, J.-S. Huang, Synthesis, structure, and optical properties of the quaternary diamond-like compounds I2–II–IV–VI4 (I= Cu; II= Mg; IV= Si, Ge; VI= S, Se), Journal of Solid State Chemistry, 204 (2013) 251-256.
[31] B. Bekki, K. Amara, M. El Keurti, First-principles study of the new potential photovoltaic absorber: Cu2MgSnS4 compound, Chinese Physics B, 26 (2017) 076201.
[32] B. Bekki, K. Amara, N. Marbouh, F. Khelfaoui, Y. Benallou, M. Elkeurti, A. Bentayeb, Theoretical study of structural, elastic and thermodynamic properties of Cu2MgSnX4 (X= S, Se and Te) quaternary compounds, Computational Condensed Matter, 18 (2019) e00339.
[33] C.D. Brunetta, B. Karuppannan, K.A. Rosmus, J.A. Aitken, The crystal and electronic band structure of the diamond-like semiconductor Ag2ZnSiS4, Journal of alloys and compounds, 516 (2012) 65-72.

[34] P. Mangelis, A. Aziz, I. da Silva, R. Grau-Crespo, P. Vaqueiro, A.V. Powell, Understanding the origin of disorder in kesterite-type chalcogenides A 2 ZnBQ 4 (A= Cu, Ag; B= Sn, Ge; Q= S, Se): The influence of inter-layer interactions, Physical Chemistry Chemical Physics, 21 (2019) 19311-19317.
[35] C.D. Brunetta, Structure determination by X-ray diffraction methods and physicochemical characterization of quaternary diamond-like semiconductors, (2013).
[36] S. Saidi, S. Zriouel, L. Drissi, M. Maaroufi, First principles study of electronic and optical properties of Ag2CdSnS4 chalcogenides for photovoltaic applications, Computational Materials Science, 152 (2018) 291-299.
[37] S. Saidi, S. Zriouel, L. Drissi, M. Maaroufi, A DFT study of electro-optical properties of kesterite Ag2CdSnX4 for photovoltaic applications, Physica E: Low-dimensional Systems and Nanostructures, 103 (2018) 171-179.
[38] O. Parasyuk, S. Chykhrij, V. Bozhko, L. Piskach, M. Bogdanyuk, I. Olekseyuk, L. Bulatetska, V. Pekhnyo, Phase diagram of the Ag2S–HgS–SnS2 system and single crystal preparation, crystal structure and properties of Ag2HgSnS4, Journal of alloys and compounds, 399 (2005) 32-37.
[39] T.V. Vu, A. Lavrentyev, B. Gabrelian, O. Parasyuk, V. Ocheretova, O. Khyzhun, Electronic structure and optical properties of Ag2HgSnSe4: First-principles DFT calculations and X-ray spectroscopy studies, Journal of Alloys and Compounds, 732 (2018) 372-384.
[40] A. Reshak, O. Khyzhun, I. Kityk, A. Fedorchuk, H. Kamarudin, S. Auluck, O. Parasyuk, Electronic structure of quaternary chalcogenide Ag2In2Ge (Si) S6 single crystals and the influence of replacing Ge by Si: experimental X-ray photoelectron spectroscopy and X-ray diffraction studies and theoretical calculations, Science of Advanced Materials, 5 (2013) 316-327.
[41] G. Myronchuk, O. Zamuruyeva, O. Parasyuk, I. Kityk, P. Czaja, M. Piasecki, The effect of composition on photoconductivity and nonlinear optical properties in the acentric Ag2In2AB6 (A= Si, Ge, B= S, Se) crystals, Optik, 179 (2019) 948-956.
[42] M.Y. Rudysh, P. Shchepanskyi, A. Fedorchuk, M. Brik, C.-G. Ma, G. Myronchuk, M. Piasecki, First-principles analysis of physical properties anisotropy for the Ag2SiS3 chalcogenide semiconductor, Journal of Alloys and Compounds, 826 (2020) 154232.
[43] A.H. Reshak, M. Jamal, DFT calculation for elastic constants of orthorhombic structure within WIEN2K code: A new package (ortho-elastic), Journal of alloys and compounds, 543 (2012) 147-151.
[44] L.M. de Chalbaud, G.D. De Delgado, J. Delgado, A. Mora, V. Sagredo, Synthesis and single-crystal structural study of Cu2GeS3, Materials research bulletin, 32 (1997) 1371-1376.
[45] G. Marcano, C. Rincón, G. Marín, G. Delgado, A. Mora, J.L. Herrera-Perez, J. Mendoza-Alvarez, P. Rodríguez, Raman scattering and X-ray diffraction study in Cu2GeSe3, Solid state communications, 146 (2008) 65-68.
[46] R. Ávila-Godoy, D. Acosta-Najarro, G. Camargo-E, C. Magaña-Zavala, L. Nieves, S. Paredes-Dugarte, B. Hidalgo-Prada, Structural Characterization of the Layered Compound Ag2SnSe3 from Scanning and Transmission Electron Microscopy and Synchrotron Powder Diffraction, Acta Microscopica, 27 (2018) 76-82.
[47] G.-G.P. Braulio, Synthesis, thermal analysis and structural characterization of the ternary compound Ag.
[48] P. Toffoli, P. Khodadad, N. Rodier, Structure d'une deuxième variété d'hexathiodiphosphate (IV) d'argent, Ag4P2S6. Comparaison des structures des deux variétés, Acta Crystallographica Section C: Crystal Structure Communications, 39 (1983) 1485-1488.
[49] P. Toffoli, P. Khodadad, N. Rodier, Structure cristalline de l'hexasélénohypodiphosphate d'argent, Ag4P2Se6, Acta Crystallographica Section B: Structural Crystallography and Crystal Chemistry, 34 (1978) 1779-1781.
[50] W. Yin, A.K. Iyer, C. Li, J. Yao, A. Mar, Noncentrosymmetric chalcogenides BaZnSiSe4 and BaZnGeSe4 featuring one-dimensional structures, Journal of Alloys and Compounds, 708 (2017) 414-421.

[51] Y. Guo, F. Liang, M. Zhou, Z. Lin, J. Yao, Y. Wu, BaM2As2S6 (M= Cd, Hg): Synthesis, crystal structure, optical and electronic properties, Journal of Alloys and Compounds, 762 (2018) 143-148.
[52] X. Wu, X. Gu, H. Pan, Y. Hu, K. Wu, Synthesis, crystal structures, optical properties and theoretical calculations of two metal chalcogenides Ba2AlSbS5 and Ba2GaBiSe5, Crystals, 8 (2018) 165.
[53] C. Li, X. Li, H. Huang, J. Yao, Y. Wu, Ba2AsGaSe5: A New Quaternary Selenide with the Novel [AsGaSe5] 4–Cluster and Interesting Photocatalytic Properties, Inorganic chemistry, 54 (2015) 9785-9789.
[54] W. Yin, D. Zhang, M. Zhou, A.K. Iyer, J.-H. Pöhls, J. Yao, A. Mar, Quaternary rare-earth selenides Ba2REGaSe5 and Ba2REInSe5, Journal of Solid State Chemistry, 265 (2018) 167-175.
[55] X.W. Lei, M. Yang, S.Q. Xia, X.C. Liu, M.Y. Pan, X. Li, X.T. Tao, Synthesis, Structure and Bonding, Optical Properties of Ba4MTrQ6 (M= Cu, Ag; Tr= Ga, In; Q= S, Se), Chemistry–An Asian Journal, 9 (2014) 1123-1131.
[56] Z. Xia, H. Fang, X. Zhang, M.S. Molokeev, R. Gautier, Q. Yan, S.-H. Wei, K.R. Poeppelmeier, CsCu5Se3: A copper-rich ternary chalcogenide semiconductor with nearly direct band gap for photovoltaic application, Chemistry of Materials, 30 (2018) 1121-1126.
[57] B. Sun, J. He, X. Zhang, K. Bu, C. Zheng, F. Huang, Synthesis, crystal structure and optical properties of K2Cu2GeS4, Journal of Alloys and Compounds, 725 (2017) 557-562.
[58] K.M. Wong, W. Khan, M. Shoaib, U. Shah, S.H. Khan, G. Murtaza, Ab Initio Investigation of the Structural, Electronic and Optical Properties of the Li 2 In 2 XY 6 (X= Si, Ge; Y= S, Se) Compounds, Journal of Electronic Materials, 47 (2018) 566-576.
[59] H. Lin, J.-N. Shen, W.-W. Zhu, Y. Liu, X.-T. Wu, Q.-L. Zhu, L.-M. Wu, Two new phases in the ternary RE–Ga–S systems with the unique interlinkage of GaS 4 building units: synthesis, structure, and properties, Dalton Transactions, 46 (2017) 13731-13738.
[60] Y. Dou, Y. Chen, Z. Li, A.K. Iyer, B. Kang, W. Yin, J. Yao, A. Mar, SrCdGeS4 and SrCdGeSe4: promising infrared nonlinear optical materials with congruent-melting behavior, Crystal Growth & Design, 19 (2019) 1206-1214.
[61] T.V. Vu, A. Lavrentyev, B. Gabrelian, O. Parasyuk, O. Khyzhun, TlInGe 2 S 6, A Prospective Nonlinear Optical Material: First-Principles DFT Calculations of the Electronic Structure and Optical Properties, Journal of Electronic Materials, 47 (2018) 5525-5536.
[62] T.V. Vu, A. Lavrentyev, B. Gabrelian, O. Parasyuk, O. Khyzhun, First-principles DFT calculations of the electronic structure and optical properties of TlInGe2Se6, a prospective NLO material, Materials Chemistry and Physics, 219 (2018) 162-174.
[63] T.V. Vu, A. Lavrentyev, B. Gabrelian, H.D. Tong, O. Parasyuk, O. Khyzhun, Electronic band structure and basic optical constants of TlGaSn2Se6, a promising NLO semiconductor: First-principles calculations under DFT framework, Optik, 181 (2019) 673-685.
[64] C.R. Sankar, A. Assoud, H. Kleinke, New Layered-Type Quaternary Chalcogenides, Tl2Pb MQ 4 (M= Zr, Hf; Q= S, Se): Structure, Electronic Structure, and Electrical Transport Properties, Inorganic Chemistry, 52 (2013) 13869-13874.
[65] A. Selezen, I. Olekseyuk, G. Myronchuk, O. Smitiukh, L. Piskach, Synthesis and structure of the new semiconductor compounds Tl2BIIDIVX4 (BII–Cd, Hg; DIV–Si, Ge; X–Se, Te) and isothermal sections of the Tl2Se–CdSe-Ge (Sn) Se2 systems at 570 K, Journal of Solid State Chemistry, (2020) 121422.
[66] M.Y. Mozolyuk, L. Piskach, A. Fedorchuk, I. Olekseyuk, O. Parasyuk, The Tl2Se–HgSe–GeSe2 system and the crystal structure of Tl2HgGeSe4, Chemistry of metals and alloys, (2013) 55-62.
[67] M.Y. Mozolyuk, L. Piskach, A. Fedorchuk, I. Olekseyuk, O. Parasyuk, Physico-chemical interaction in the Tl2Se–HgSe–DIVSe2 systems (DIV–Si, Sn), Materials Research Bulletin, 47 (2012) 3830-3834.
[68] A. Lavrentyev, B. Gabrelian, T.V. Vu, L. Ananchenko, G. Myronchuk, O. Parasyuk, V. Tkach, K. Kopylova, O. Khyzhun, Electronic and optical properties of quaternary sulfide Tl2HgSnS4, a promising optoelectronic semiconductor: A combined experimental and theoretical study, Optical Materials, 92 (2019) 294-302.

[69] X. Zhang, D. Chen, K. Deng, R. Lu, Band engineering of wurtzite-derived semiconductors Cu2ZnSiS4 and Cu2ZnSiSe4, Journal of Alloys and Compounds, 656 (2016) 196-199.
[70] X. Zhang, D. Rao, R. Lu, K. Deng, D. Chen, First-principles study on electronic and optical properties of Cu2ZnSiV I4 (VI= S, Se, and Te) quaternary semiconductors, AIP Advances, 5 (2015) 057111.
[71] N. Kodan, S. Auluck, B. Mehta, A DFT study of the electronic and optical properties of a photovoltaic absorber material Cu2ZnGeS4 using GGA and mBJ exchange correlation potentials, Journal of Alloys and Compounds, 675 (2016) 236-243.
[72] D. Chen, N. Ravindra, Electronic and optical properties of Cu2ZnGeX4 (X= S, Se and Te) quaternary semiconductors, Journal of alloys and compounds, 579 (2013) 468-472.
[73] G.K. Gupta, R. Chaurasiya, A. Dixit, Theoretical studies on structural, electronic and optical properties of kesterite and stannite Cu2ZnGe (S/Se) 4 solar cell absorbers, Computational Condensed Matter, 19 (2019) e00334.
[74] C.-R. Li, Y.-F. Li, B. Yao, G. Yang, Z.-H. Ding, R. Deng, L. Liu, Electronic and optical properties of kesterite Cu2ZnSnS4 under in-plane biaxial strains: First-principles calculations, Physics Letters A, 377 (2013) 2398-2402.
[75] H.-R. Liu, S. Chen, Y.-T. Zhai, H.-J. Xiang, X.-G. Gong, S.-H. Wei, First-principles study on the effective masses of zinc-blend-derived C u 2 Z n− IV− VI 4 (IV= Sn, Ge, Si and VI= S, Se), Journal of Applied Physics, 112 (2012) 093717.
[76] S. Ullah, H.U. Din, G. Murtaza, T. Ouahrani, R. Khenata, S.B. Omran, Structural, electronic and optical properties of AgXY2 (X= Al, Ga, In and Y= S, Se, Te), Journal of alloys and compounds, 617 (2014) 575-583.
[77] A. Lavrentyev, B. Gabrelian, V. Vu, P. Shkumat, V. Ocheretova, O. Parasyuk, O. Khyzhun, Electronic structure and optical properties of Cu2CdGeS4: DFT calculations and X-ray spectroscopy measurements, Optical Materials, 47 (2015) 435-444.
[78] H. Ahmoum, M. Su'ait, G. Li, S. Chopra, M. Boughrara, Q. Wang, M. Kerouad, D. Rai, Electronic and thermoelectric properties of chalcopyrite compounds Cu 2 (XY) S 4 (X= Zn, Cd and Y= Sn, Pb): first-principles study, Indian Journal of Physics, (2020) 1-7.
[79] F. Liu, J. Zheng, M. Huang, L. He, W. Ao, F. Pan, J. Li, Enhanced thermoelectric performance of Cu 2 CdSnSe 4 by Mn doping: experimental and first principles studies, Scientific reports, 4 (2014) 1-7.
[80] N. Sarmadian, R. Saniz, B. Partoens, D. Lamoen, First-principles study of the optoelectronic properties and photovoltaic absorber layer efficiency of Cu-based chalcogenides, Journal of Applied Physics, 120 (2016) 085707.
[81] D. Li, X. Zhang, Z. Zhu, H. Zhang, F. Ling, Ab initio calculations of structural, electronic, and optical properties of Cu2HgSnSe4, Solid state sciences, 14 (2012) 890-893.
[82] S. Azam, M. Irfan, Z. Abbas, S.A. Khan, I.V. Kityk, T. Kanwal, M. Sohail, S. Muhammad, A.G. Al-Sehemi, Effect of S and Se replacement on electronic and thermoelectric features of BaCu2GeQ4 (Q= S, Se) chalcogenide crystals, Journal of Alloys and Compounds, 790 (2019) 666-674.
[83] Y. Yang, K. Wu, X. Wu, B. Zhang, L. Gao, A new family of quaternary thiosilicates SrA 2 SiS 4 (A= Li, Na, Cu) as promising infrared nonlinear optical crystals, Journal of Materials Chemistry C, 8 (2020) 1762-1767.
[84] F. Hong, W. Lin, W. Meng, Y. Yan, Trigonal Cu 2-II-Sn-VI 4 (II= Ba, Sr and VI= S, Se) quaternary compounds for earth-abundant photovoltaics, Physical Chemistry Chemical Physics, 18 (2016) 4828-4834.
[85] S. Sharma, P. Kumar, Quaternary semiconductors Cu2MgSnS4 and Cu2MgSnSe4 as potential thermoelectric materials, Journal of Physics Communications, 1 (2017) 045014.
[86] M. Rasukkannu, D. Velauthapillai, P. Vajeeston, Hybrid Density Functional Study of Au2Cs2I6, Ag2GeBaS4, Ag2ZnSnS4, and AgCuPO4 for the Intermediate Band Solar Cells, Energies, 11 (2018) 3457.

[87] S. Nakamura, T. Maeda, T. Tabata, T. Wada, First-principles study of indium-free photovoltaic compounds Ag 2 ZnSnSe 4 and Cu 2 ZnSnSe 4, in: 2011 37th IEEE Photovoltaic Specialists Conference, IEEE, 2011, pp. 002771-002774.
[88] L. Wei, W. Fan, Y. Li, X. Zhao, L. Yang, Effect of cation ordering on the electronic and lattice dynamic properties of Ag2CdGeS4 polytypes: First-principle calculation, Journal of Solid State Chemistry, 201 (2013) 48-55.
[89] L. Dan, Z. Xing-Hong, Theoretical studies on the structural, electronic, and optical properties of Ag2HgSnSe4, Chinese Physics B, 20 (2011) 126102.
[90] H. Shao, X. Tan, T. Hu, G.-Q. Liu, J. Jiang, H. Jiang, First-principles study on the lattice dynamics and thermodynamic properties of Cu2GeSe3, EPL (Europhysics Letters), 109 (2015) 47004.
[91] M. Ali, M.A. Hossain, M. Rayhan, M. Hossain, M. Uddin, M. Roknuzzaman, K. Ostrikov, A. Islam, S. Naqib, First-principles study of elastic, electronic, optical and thermoelectric properties of newly synthesized K2Cu2GeS4 chalcogenide, Journal of Alloys and Compounds, 781 (2019) 37-46.
[92] S. Azam, S.A. Khan, J. Minar, W. Khan, H.U. Din, R. Khenata, G. Murtaza, S. Bin-Omran, S. Goumri-Said, Coulomb interaction and spin-orbit coupling calculations of thermoelectric properties of the quaternary chalcogenides Tl2PbXY4 (X= Zr, Hf and Y= S, Se), Semiconductor Science and Technology, 30 (2015) 105018.
